# Development of a BCL-xL and BCL-2 dual degrader with improved anti-leukemic activity,

**DOI:** 10.1038/s41467-021-27210-x

**Published:** 2021-11-25

**Authors:** Dongwen Lv, Pratik Pal, Xingui Liu, Yannan Jia, Dinesh Thummuri, Peiyi Zhang, Wanyi Hu, Jing Pei, Qi Zhang, Shuo Zhou, Sajid Khan, Xuan Zhang, Nan Hua, Qingping Yang, Sebastian Arango, Weizhou Zhang, Digant Nayak, Shaun K. Olsen, Susan T. Weintraub, Robert Hromas, Marina Konopleva, Yaxia Yuan, Guangrong Zheng, Daohong Zhou

**Affiliations:** 1 Department of Pharmacodynamics, College of Pharmacy, University of Florida, Gainesville, FL USA; 2 Department of Medicinal Chemistry, College of Pharmacy, University of Florida, Gainesville, FL USA; 3 Department of Leukemia, University of Texas M.D. Anderson Cancer Center, Houston, TX USA; 4 Department of Pathology, Immunology and Laboratory Medicine, College of Medicine, University of Florida, Gainesville, FL USA; 5 Department of Biochemistry & Structure Biology, Long School of Medicine, University of Texas Health Science Center at San Antonio, San Antonio, TX USA; 6 Mays Cancer Center, the Long School of Medicine, University of Texas Health Science Center at San Antonio, San Antonio, TX USA

**Keywords:** Proteolysis, Molecular modelling, Leukaemia, Structure-based drug design

## Abstract

PROteolysis-TArgeting Chimeras (PROTACs) have emerged as an innovative drug development platform. However, most PROTACs have been generated empirically because many determinants of PROTAC specificity and activity remain elusive. Through computational modelling of the entire NEDD8-VHL Cullin RING E3 ubiquitin ligase (CRL^VHL^)/PROTAC/BCL-xL/UbcH5B(E2)-Ub/RBX1 complex, we find that this complex can only ubiquitinate the lysines in a defined band region on BCL-xL. Using this approach to guide our development of a series of ABT263-derived and VHL-recruiting PROTACs, we generate a potent BCL-xL and BCL-2 (BCL-xL/2) dual degrader with significantly improved antitumor activity against BCL-xL/2-dependent leukemia cells. Our results provide experimental evidence that the accessibility of lysines on a target protein plays an important role in determining the selectivity and potency of a PROTAC in inducing protein degradation, which may serve as a conceptual framework to guide the future development of PROTACs.

## Abstract (web summary)

Simultaneous targeting of BCL-xL and BCL-2 is an attractive approach for cancer treatment. Based on information gained by computational structure modelling, the authors develop a PROTAC that induces degradation of both BCL-xL and BCL-2 and effectively targets BCL-xL/2-dependent leukaemia cells.

## Introduction

Proteolysis-targeting chimeras (PROTACs) are small molecule degraders that can degrade a protein of interest (POI) through the ubiquitin-proteasome system (UPS) by targeting the POI to an E3 ubiquitin ligase for ubiquitination and subsequent degradation by proteasomes. PROTACs have many advantages as potential therapeutics compared to conventional small molecule inhibitors (SMIs). This is largely attributable to their unique mechanism of action of degrading POIs in a catalytic manner^1^. In addition, they have the ability to target undruggable and mutant proteins, to improve target selectivity, and to extend duration of action^1^. More importantly, by targeting a POI to a cell/tissue selective/specific E3 ligase for degradation, PROTACs can overcome the on-target and dose-limiting toxicity of SMIs^2^. Therefore, PROTACs have become an emerging technology for the development of unique targeted anticancer therapeutics^3,4^.

Although a number of PROTACs have been developed to efficiently degrade various cancer targets, their development remains empirical because the determinants of an efficient PROTAC-mediated protein degradation remain poorly characterized. By solving the crystal structure of MZ1, a Brd4 degrader, with von Hippel-Lindau protein (VHL) and Brd4 bromodomain 2 (Brd4^BD2^), Gadd et al. revealed that the formation of a stable and cooperative E3 ligase-PROTAC-POI ternary complex is important for PROTACs to effectively and specifically degrade their target^5^. This led to the development of a Brd4 PROTAC (AT1) with improved potency and selectivity, demonstrating the power of using structure-guided design to facilitate the development of PROTACs^5,6^. However, the induction of a stable ternary complex by a PROTAC does not always lead to the degradation of its target. For example, a VHL-recruiting PROTAC using the promiscuous kinase inhibitor Foretinib as its warhead can induce stable interactions of c-Abl and Arg with VHL, but cannot degrade either^7^. Similar findings have been reported for a number of VHL-recruiting PROTACs that can selectively degrade one of their targets despite their ability to induce strong interactions with VHL and their non-degradable targets^8–12^. One of the possible mechanisms for PROTACs to achieve target selectivity could, at least in part, be attributed to the differential distribution and orientation of lysines on the surface of different targets, resulting in some of the lysines becoming inaccessible to the E2 component for ubiquitination^8–12^. This hypothesis is supported by the finding that only certain surface exposed lysines on CK1α are accessible to the E2 recruited by lenalidomide via the cereblon (CRBN) E3 ligase for ubiquitination^13^. In addition, it has been proposed that some E3 ligases cover a relatively narrower region on POIs, hence providing fewer potential surface lysines to be ubiquitinated and leading to a higher substrate selectivity; whereas E3 ligases with a broader area of action have lower lysine and substrate specificity^7,14^. However, the hypothesis that lysine accessibility determines PROTAC-inducing protein degradation, which in turn contributes to the selectivity of PROTACs, has not been experimentally validated.

BCL-xL and BCL-2 belong to the anti-apoptotic BCL-2 protein family and play an important role in promoting tumor initiation, progression, and development of drug resistance by protecting tumor cells from apoptosis^15^. Inhibition of these BCL-2 family proteins with SMIs has been extensively investigated as a therapeutic strategy for cancers^16–21^, resulting in the discovery of several inhibitors as promising anticancer drug candidates, including ABT263 (navitoclax, a BCL-xl/2 dual inhibitor). Unfortunately, ABT263 has not obtained regulatory approval for clinical use because the inhibition of BCL-xL induces on-target and dose-limiting thrombocytopenia^21–23^. To overcome this toxicity, we recently converted ABT263 into a VHL-recruiting PROTAC, DT2216, that potently degrades BCL-xL but not BCL-2 despite binding to both proteins with high affinities^2^. In addition, we found that K87 is the only lysine ubiquitinated by DT2216 albeit there are four lysines on the surface of BCL-xL and two on BCL-2. Although DT2216 is very useful for the treatment of some BCL-xL-dependent T-cell acute lymphoblastic leukemia and T-cell lymphoma, it has a limited effect on other leukemias and most solid tumors unless combined with ABT199 (venetoclax, a selective BCL-2 inhibitor) or chemotherapy^2^. This is because these leukemias and solid tumors depend on both BCL-xL and BCL-2 for survival. Therefore, PROTACs that can degrade both BCL-xL and BCL-2 should have a broader application for the treatment of leukemia and solid tumors than DT2216.

Although BCL-xL and BCL-2 are homologous proteins sharing about 45% sequence identity^24^, they have distinct surface lysine distributions with only one conserved surface lysine^25^. The structural similarity, as well as the differential lysine distribution and the limited number of lysines on their surfaces, make BCL-xL/2 an ideal model system to study how the accessibility of lysines to the E2 component of the ubiquitination complex influences the degradation of a POI by PROTACs. Based on the computational modeling of the entire NEDD8-VHL Cullin RING E3 ubiquitin ligase (CRL^VHL^)/DT2216/BCL-xL/UbcH5B(E2)-Ub/RBX1 complex structure, the possible motion modes of the constructed large complex has been predicted with normal model analysis algorithm^26^. Presumably, not all lysines on the surface of POI are available for ubiquitination because the appropriate spatial proximity between the substrate lysine with the narrow catalytic site on E2 is indispensable for occurring ubiquitination reaction. Moreover, even the surface lysines available for ubiquitination are not necessarily equivalent in ubiquitination preference, because the ubiquitination transfer reaction should be physically dependent on the formation of the Ub-E2/POI complex (at least a transient transition complex) in a very specific reaction-ready state. Thus, the protonated state, flexibility, and orientation of substrate lysine as well as the other residues around the lysine involved in the E2/POI interface would also be key factors contributing to the preference of the lysine ubiquitination specificity. Based on this assumption, we hypothesize that changing the lysine accessibility and preference in forming the E2/POI complex by altering the conformation of the E3/PROTAC/POI ternary complex may influence the specificity and efficiency of a PROTAC. With the analysis of the binding mode of ABT263 to BCL-xL, we have selected a different PROTAC linker site on ABT263—which is also solvent-exposed but distant from the linker site in DT2216—to allow for a different E3/PROTAC/POI ternary conformation. This has led to the generation of a BCL-xL/2 dual degrader, which is more potent than DT2216 against leukemia cells that depend on BCL-xL/2 for survival.

## Results

### Requirement of accessible lysines for BCL-xL degradation

**Fig. 1:**
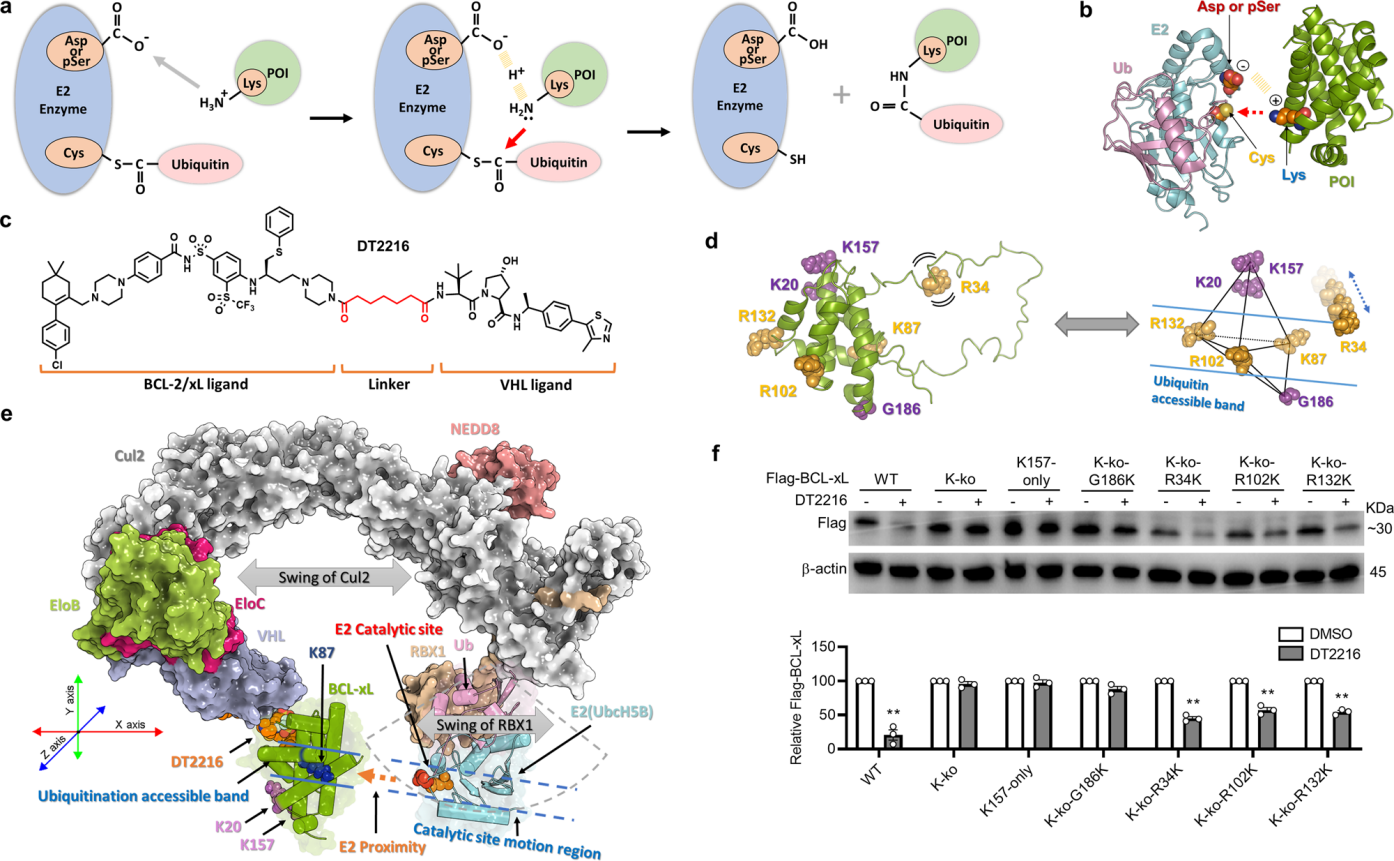
A band region of accessible lysines on BCL-xL. **a** A theoretical model of ubiquitination reaction. The protein of interest (POI), ubiquitin, and E2 enzyme are colored in green, pink, and blue, respectively. The conserved E2 serine/aspartate (CES/D) site, catalytic cysteine, and target lysine are showed in orange balls. The target lysine is firstly attracted to the CES/D site by electrostatic interaction, and subsequently attack the thioester bond between ubiquitin and E2 enzyme. **b** Atomic details of the catalytic mechanism. **c** The structure of DT2216. **d** A predicted band region of E2-accessible lysines on BCL-xL. The six selected lysine residues or lysine point mutation sites around BCL-xL are shown in sphere style. R34K, R102K, R132K, and K87 are in the predicted band region, and K20, K157, and G186K are outside of the band region. **e** A computational model of the entire CRL^VHL^/DT2216/BCL-xL/E2-Ub/RBX1 complex. The possible motion region of E2 is shown with dashed sector. The catalytic site motion region is represented by two blue dashed lines. The accessible region of the ubiquitination is represented by the two blue lines along the direction of E2 proximity to POI represented by an orange dashed arrow. The potential direction of swing of Cul2 and RBX1 are shown by a gray double-headed arrow. **f** The point mutation analyses confirm the existence of a band region of lysines on BCL-xL. WT: wild type Flag-BCL-xL; K-ko: all the surface lysines on BCL-xL are mutated to arginines; K157-only: all the surface lysines on BCL-xL are mutated to arginines except K157; K-ko-G186K, K-ko-R34K, K-ko-R102K, and K-ko-R132K represent that all the surface lysines on BCL-xL are mutated to arginines and then Gly186, Arg34, Arg102, and Arg132 are respectively mutated to lysine. β-actin was used as an equal loading control. The quantification of the relative Flag-BCL-xL protein content in the immunoblots is presented as mean values ± s.e.m. (*n* = 3 biologically independent experiments) in the bar graph (bottom panel). Statistical significance was calculated with unpaired two-tailed Student’s *t*-tests comparing DMSO- to DT2216-treated samples. **P < 0.01; *P* values were 0.0089, 0.0023, 0.0077, and 0.0026 for WT, K-ko-R34K, K-ko-R102K, and K-ko-R132K, respectively. Source data are provided as a Source data file.

Recently, we reported the development of DT2216, a potent VHL-based BCL-xL PROTAC, as a safer and more potent antitumor agent than its warhead ABT263^2^. Interestingly, we found that DT2216 can degrade BCL-xL but not BCL-2 in part because DT2216 cannot form a ternary complex with BCL-2 and VHL in cells. However, the lack of the formation of a stable ternary complex may not be the sole factor affecting the degradability of BCL-2 by PROTACs because XZ739 (Supplementary Fig. 1a), a potent CRBN-recruiting BCL-xL PROTAC^27^ that formed cellular ternary complexes with both BCL-xL and BCL-2, could degrade BCL-xL but not BCL-2 (Supplementary Fig. 1b, c). More importantly, we found that DT2216 degraded BCL-xL in a K87 ubiquitination-dependent manner^2^, even though there are three other surface lysines on BCL-xL (Supplementary Fig. 2a–c). These findings demonstrate that DT2216 has a lysine preference for inducing BCL-xL ubiquitination and degradation. This preference may be attributable to the accessibility of the preferred lysine on BCL-xL to E2 for the transfer of ubiquitin, which fits one of the theoretical models of ubiquitination reaction^28^ (Fig. 1a, b). In this model, the conserved E2 serine/aspartate (CES/D) active site which contains a negatively charged aspartate or a phosphorylated serine on E2 plays a key role in attracting and aligning of the positively charged substrate lysine toward the catalytic cysteine on E2^28^. At the transition state, the aspartate or the phosphorylated serine promotes deprotonation of the substrate lysine, which allows its nucleophilic attack of the thioester bond between a donor ubiquitin and E2 enzyme. Finally, the C-terminal of the donor ubiquitin is transferred to the target acceptor lysine to form an amide bond. As such, the transfer of a ubiquitin to a POI is strictly dependent on the appropriate orientation of the target lysine on the POI to allow its specific interaction with the E2 catalytic site, instead of a simple spatial proximity of the E2 enzyme to the POI. Therefore, the accessibility of a surface lysine on a POI to the catalytic site of E2 plays a critical role in determining whether the corresponding lysine is a potential site for ubiquitin transfer and thus the degradability of the POI.

To evaluate this hypothesis, we built a computational model of the entire CRL^VHL^/DT2216/BCL-xL/E2-Ub/RBX1 complex to assess the accessibility of the surface lysines on BCL-xL (Fig. 1c–f). Based on this model, the E2-Ub component of the complex is tied to the Cul2 with an unfolded hinge loop region (about 5 aa), which is expected to make the E2 relatively flexible at a certain region with different loop conformation. Therefore, the RBX1-induced flexibility makes the catalytic site of E2 accessible in a certain region, hereby defined as the “catalytic site motion region”. In addition, according to the normal mode analysis of the entire complex, we observed two major motion modes of the complex (Supplementary Fig. 2d, e and Supplementary Movies 1 and 2). Specifically, the first motion mode mainly represents the open-close motion (Supplementary Fig. 2d and Supplementary Movie 1), which demonstrates that the E2 can move closer or away from BCL-xL (along the X-axis in Fig. 1e). The second motion mode mainly represents the twisting motion of the Cullin backbone (Supplementary Fig. 2e and Supplementary Movie 2), which permits the swing of the E2 and BCL-xL in an opposite direction (vertical to the Cullin backbone plain, along the Z-axis in Fig. 1e). These two motion modes enlarge the potentially accessible region of the E2 catalytic site, which extends the previously defined “catalytic site motion region”. Combining both major motion modes of the E2 and its accessible motion region, it is reasonable to suggest that the “catalytic site motion region” only covers a small band region of BCL-xL. We hypothesize that only the BCL-xL surface lysines located within the band region are accessible to the catalytic site of E2. Therefore, we constructed various BCL-xL mutants that each possessed only one lysine located in different regions on the protein surface and then tested their degradation after DT2216 treatment. The results confirmed that both wild-type (WT) BCL-xL and the BCL-xL mutants containing lysines located within the band region, i.e., K87-only^2^, and R102K, and R132K mutants, as well as R34K that is located in the flexible loop of BCL-xL and can be accessible to the E2, can be degraded by DT2216. In contrast, K20-only^2^, G186K, and K157-only BCL-xL mutants that have a lysine located outside of the band region cannot be degraded by DT2216 (Fig. 1d–f and Supplementary Fig. 2a–c). We further confirmed that lysine to arginine mutations on the BCL-xL, such as BCL-xL-ko and K157-only BCL-xL mutants, do not interfere with the ternary complex formation (Supplementary Fig. 3). Interestingly, our previous study showed the K16 and K20 are not ubiquitinated by DT2216 and are dispensable for DT2216-induced BCL-xL degradation^2^. Although K20 is distant from K157 in sequence, they are structurally neighboring residues located outside of the band region (Fig. 1d, e), which further strengthens the rationale for a lysine accessible region required for E2-mediated ubiquitination. K16 is structurally neighboring K87, which implies that it is located within the band region. However, K16 forms a salt bridge with E98 and a hydrogen bond with D95 (Supplementary Fig. 2c). The strong electrostatic interactions may not only shield the positive charge of lysine for attracting CES/D of E2, but also prevents its prerequisite conformation adjustment for participating in the reaction.

### Development of BCL-xL and BCL-2 dual PROTACs/degraders

**Fig. 2:**
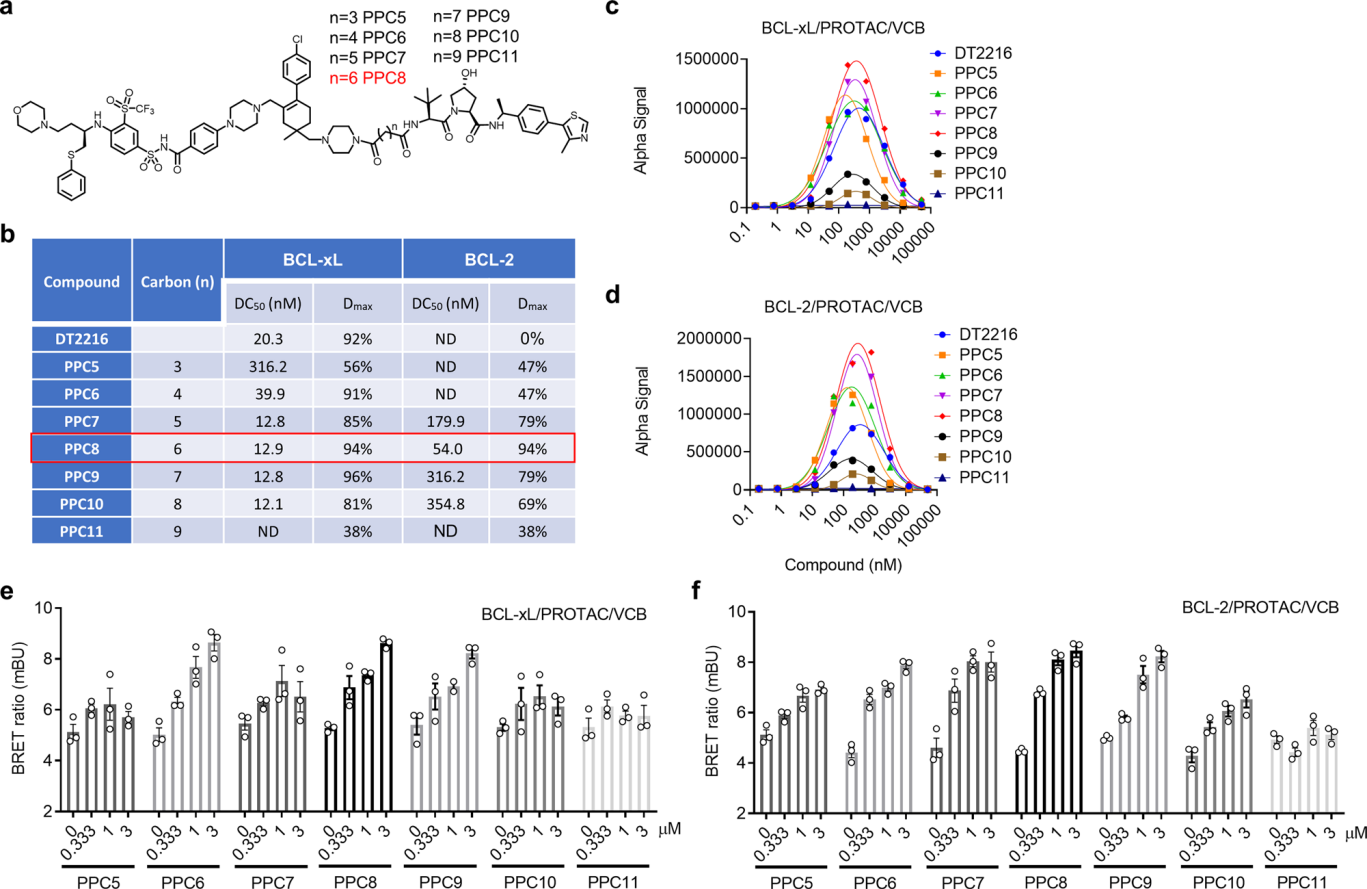
Synthesis and evaluation of a series of BCL-xL and/or BCL-2 degraders. **a** The structure of the BCL-xL and/or BCL-2 degraders (racemic mixture). **b** The BCL-xL and BCL-2 degradation profiling of the PROTACs evaluated in 293T cells after the cells were treated with four different concentrations of indicated PROTACs for 16 h. The DC_50_ and D_max_ were calculated using Prism based on the ImageJ-quantified western blots in Supplementary Fig. 4. **c**, **d** AlphaLISA assay to determine the formation of BCL-xL/PROTAC/VHL-ELOC-ELOB (VCB) (**c**) or BCL-2/PROTAC/VCB (**d**) ternary complex in a cell-free condition. Data represent the mean of a single experiment with 2 technical replicates. Similar results were obtained in one more independent experiment. **e**, **f** NanoBRET assays evaluating intracellular BCL-xL/PROTAC/VCB (**e**) or BCL-2/PROTAC/VCB (**f**) ternary complex formation. 293T cells were transiently transfected with HiBit-BCL-xL, LgBit, and HaloTag-VHL or HiBit-BCL-2, LgBit, and HaloTag-VHL and then treated with a serial dilution of the indicated compounds for 6 h. Data are expressed as mean ± s.e.m. of three biological replicates. Source data are provided as a Source data file.

Since the location of lysines and their orientation toward E2 determine the specificity/efficacy of DT2216-mediated BCL-xL degradation, it is foreseeable that using a different link-out position on ABT263 may change the geometry of BCL-xL-E3 interaction with the exposure of different lysines toward the E2 for more efficient BCL-xL degradation. To test this hypothesis, we synthesized a series of BCL-xL PROTACs with different linker lengths using the same VHL ligand and ABT263 as the warhead (Fig. 2a, b), but utilized one of the two methyl groups on the cyclohexene ring of ABT263 as the linker attachment site, a point distant from the morpholine linker position of DT2216 (Fig. 1c) (Supplementary Methods). It has been well established that the linker length is also a critical factor that determines whether a stable ternary complex can be induced by a PROTAC, which in turn determines the efficiency of the PROTAC to degrade its target. The optimal linker length for the series of PROTACs can be determined by the linker length of the compound with the highest degradation efficiency. To avoid any confounding effect of apoptosis on protein degradation, we profiled the BCL-xL/2 degradation by these compounds in HEK293T cells (Fig. 2b and Supplementary Fig. 4) that were insensitive to the induction of apoptosis by BCL-xL/2 inhibition or degradation (Supplementary Fig. 5a). We found that PPC5 with the shortest linker in this series was able to partially degrade BCL-xL (Fig. 2b and Supplementary Fig. 4). The best DC_50_ and D_max_ were achieved by PPC7, PPC8, and PPC9 (Fig. 2b and Supplementary Fig. 4). PPC11 with the longest linker in this series can only reach 38% maximal degradation of BCL-xL (Fig. 2b and Supplementary Fig. 4). These results indicated that there is an optimal linker length for the PROTACs to induce BCL-xL degradation, as seen from other PROTACs reported previously^29^. More importantly, we found that four (PPC7, PPC8, PPC9, and PPC10) of these compounds also degraded BCL-2, with PPC8 being the most potent (Fig. 2b). In vitro ternary complex formation assays showed that PPC5, PPC6, PPC7, and PPC8 can form stronger ternary complexes with both BCL-xL and BCL-2, but PPC9, PPC10, and PPC11 formed much weaker ternary complexes (Fig. 2c, d). PPC8 formed the strongest ternary complex for both BCL-xL and BCL-2 (Fig. 2c, d). We further compared their abilities to form intracellular ternary complexes using cellular NanoBRET assay and found that PPC6, PPC7, PPC8, and PPC9 formed stronger BCL-xL/PROTAC/VHL-EloC-EloB (VCB) ternary complexes than those formed by PPC5, PPC10, and PPC11 (Fig. 2e, f). Our results support the notion that the formation of intracellular ternary complex is necessary but not sufficient for the induction of BCL-xL/2 degradation and that other factors, such as the exposure of lysines and their orientation to E2, likely are critical in determining the efficacy of a PROTAC.

**Fig. 3:**
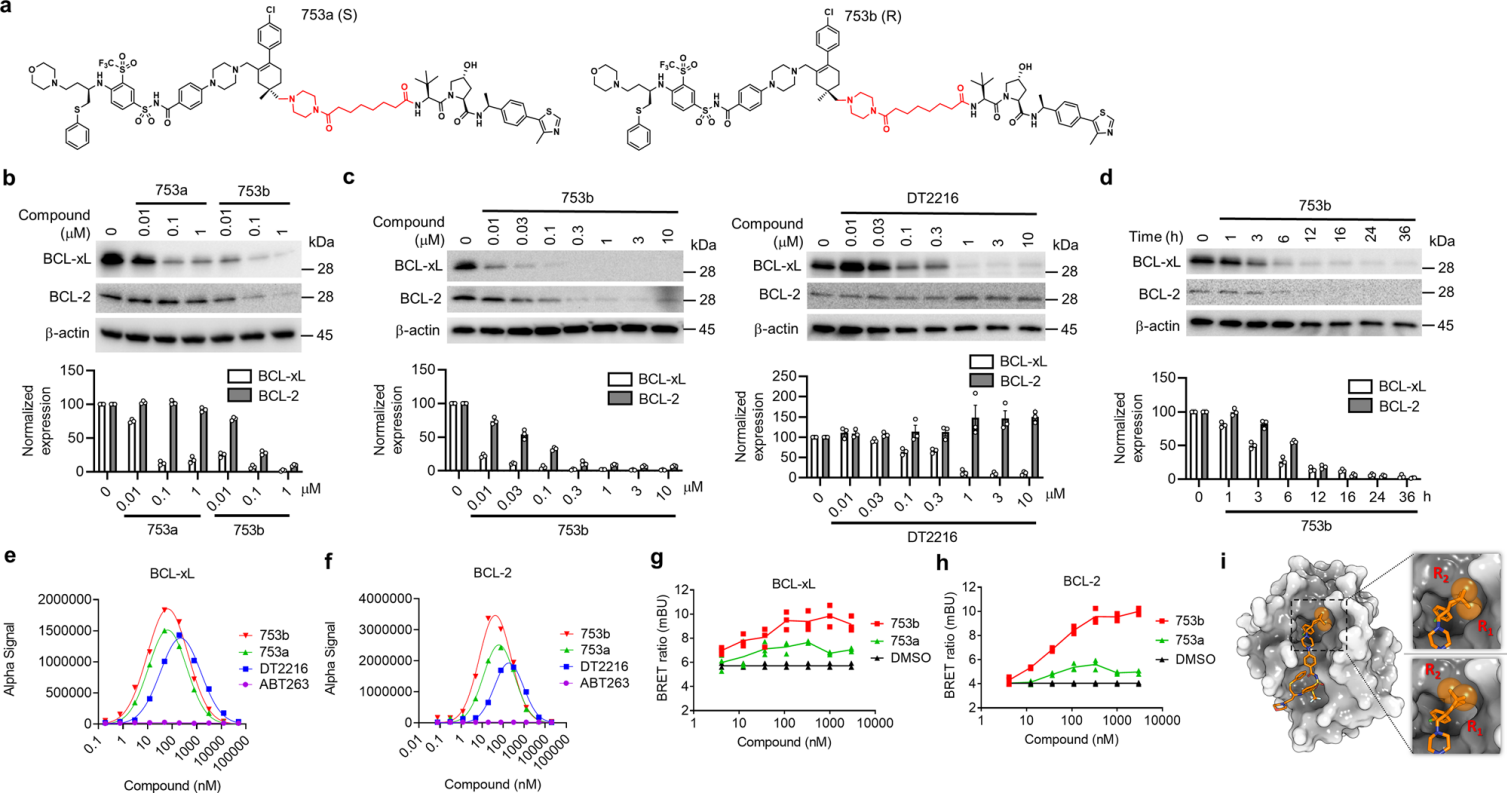
753b is a dual BCL-xL/2 degrader. **a** The structure of 753a and 753b. **b** The potency of 753a and 753b in degrading BCL-xL/2 was evaluated in 293T cells by immunoblots after the cells were treated with different concentrations of 753a or 753b for 16 h. **c** Comparison of the potencies of 753b and DT2216 in degrading BCL-xL/2 by immunoblots in 293T cells after the cells were treated with different concentrations of 753b or DT2216 for 16 h. **d** The time course of 753b-mediated BCL-xL/2 degradation was evaluated in 293T cells by immunoblots after the cells were treated with 1 μM 753b for various time points as indicated. Representative immunoblots are shown in (**a**–**d**) and β-actin was used as a loading control in all immunoblot analyses. The normalized protein content in the immunoblots is presented as mean values ± s.e.m. (*n* = 3 biologically independent experiments) in the bar graph (bottom panel). **e**, **f** The formation of BCL-xL/PROTAC/VCB (**e**) and BCL-2/PROTAC/VCB (**f**) ternary complexes in a cell-free condition was determined by AlphaLISA assay. Data represent the mean of a single experiment with 2 technical replicates. Similar results were obtained in one more independent experiment. **g**, **h** NanoBRET assays showed that 753b forms more stable ternary complexes with BCL-xL (**g**) and BCL-2 (**h**) in 293T cells compared with 753a. 293T cells were transiently transfected with HiBit-BCL-xL, LgBit, and HaloTag-VHL or HiBit-BCL-2, LgBit, and HaloTag-VHL and then treated with a serial dilution of 753a or 753b for 6 h. Data are expressed as mean of three biological replicates. **i** The different conformations of linker methyl groups on ABT263 observed in X-ray crystal structure of BCL-xL/ABT263 complex (PDB 4QNQ). Source data are provided as a Source data file.

We selected the most potent degrader PPC8 for further study due to its high potency in degrading both BCL-xL/2. PPC8 contains two mixed stereoisomers (753a and 753b) (Fig. 3a). We separated the two stereoisomers and found that the *R*-epimer, 753b, was the major active component in inducing BCL-xL/2-degradation (Fig. 3b); whereas 753a, the *S*-epimer, could only partially degrade BCL-xL but not BCL-2 (Fig. 3b). In addition, 753b exhibited a higher potency (DC_50_ = 6 nM) in degrading BCL-xL than DT2216 (DC_50_ = 30 nM) (Fig. 3c) and PPC8 (Fig. 2). More importantly, 753b also degraded BCL-2 with a DC_50_ of 48 nM; whereas DT2216 did not induce BCL-2 degradation^2^ (Fig. 3c). The 753b-mediated degradation of BCL-xL and BCL-2 was time-dependent with a similar kinetics (Fig. 3d). Similar results were also observed in Hela cells, which are also resistant to apoptosis induction by BCL-xL/2 inhibition or degradation (Supplementary Fig. 5b, c). These results demonstrated that 753b is a potent BCL-xL/2 dual degrader.

We further performed several experiments to determine the mechanism that determines the differential effects of these two stereoisomers. 753a and 753b exhibited similar binary binding affinity to BCL-xL/2, but much weaker than that of the warhead ABT263 (Supplementary Fig. 6). AlphaLISA assay showed that 753b formed stronger ternary complexes with BCL-xL/2 than those by 753a and DT2216 (Fig. 3e, f). Furthermore, cellular NanoBRET ternary complex formation assay confirmed that 753b displayed a much higher activity to induce the ternary complex formation with both BCL-xL and BCL-2 relative to 753a (Fig. 3g, h). Based on the ternary complex models of VHL/DT2216/BCL-xL and VHL/753b/BCL-xL (Supplementary Fig. 7), it is noticeable that more residues of BCL-xL and VHL are involved in the BCL-xL/VHL interface interaction in the ternary complex induced by 753b than that induced by DT2216, which may partially explain why 753b is a stronger inducer of the ternary complex than DT2216. Moreover, the conformation of the linker tethering piperazine ring in DT2216 is significantly different from the corresponding morpholine ring in 753b, which suggests that DT2216 may disturb the original binding mode of ABT263 after being converted to the PROTAC whereas 753b has kept a similar binding mode of ABT263. This difference may also partially explain why 753b is a better PROTAC than DT2216 to induce the formation of the ternary complex. As depicted in Fig. 3i, the corresponding link-out methyl groups for 753a (R_1_) and 753b (R_2_) in ABT263 showed two different conformations in X-ray crystal structure (PDB 4QNQ). Although the exact ratio of these two conformations is unknown, the energy for the two conformations would be similar because both conformations could be observed in the X-ray crystal structure. The R_2_ linker site for 753b is solvent-exposed in both conformations. However, the R_1_ linker site for 753a is solvent-exposed in one conformation and partially buried in the other conformation. Therefore, the R_2_ linker site should be more solvent-exposed than the R_1_ linker site, which may explain why 753b could induce stronger ternary complex formation than 753a. In addition, our previous study showed that DT2216 can induce the ternary formation of BCL-xL but not BCL-2 with VCB in cells^2^. These findings demonstrate that not only the link-out position and linker length but also the steric orientation of the link-out site have a major influence on the ability of ABT263-derived PROTACs to degrade their targets in part via promoting the formation of a ternary complex in cells.

### Mechanism of action of 753b

**Fig. 4:**
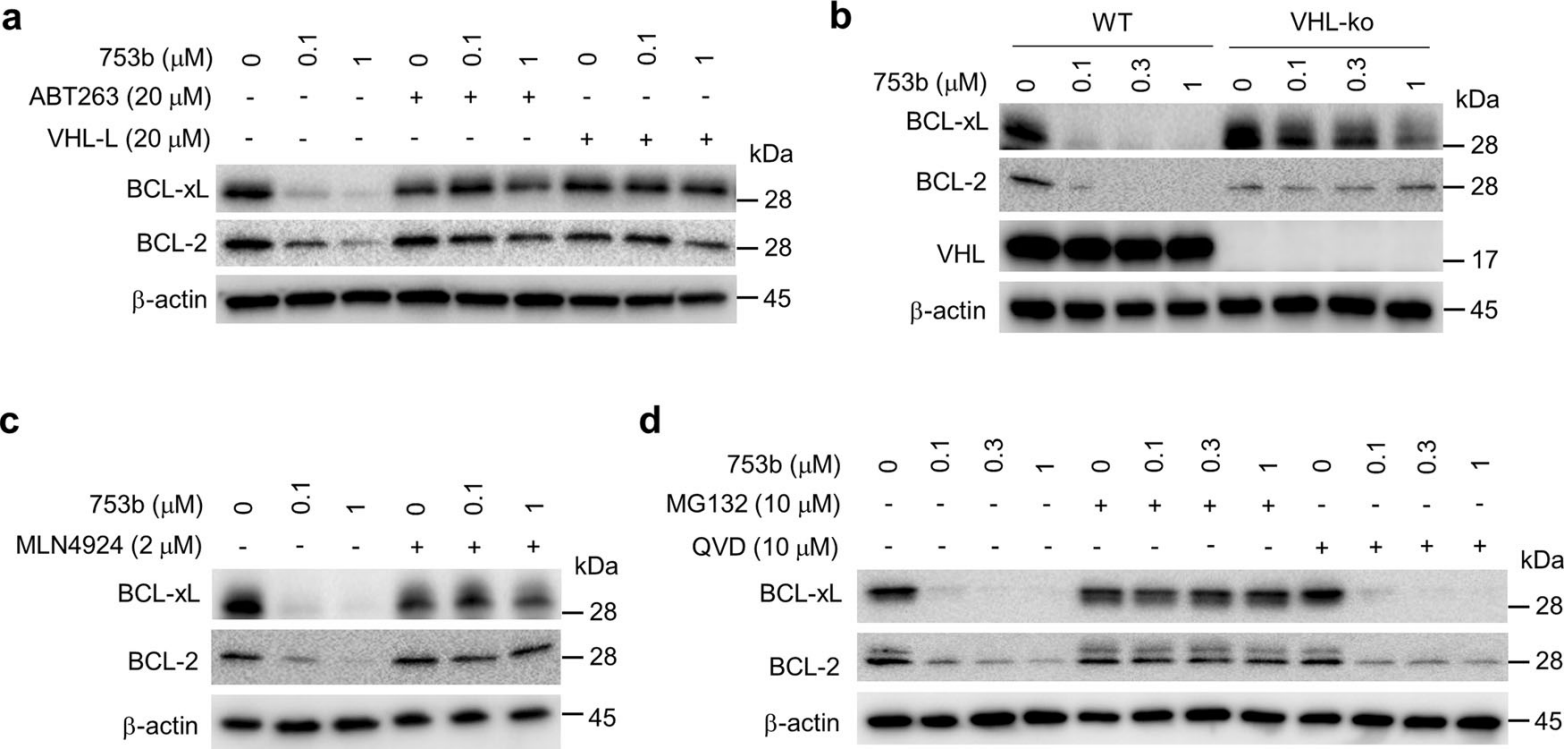
753b degrades BCL-xL/2 in a VHL- and proteasome-dependent manner. **a** Pretreatment with ABT263 or VHL-L blocks the BCL-xL/2 degradation induced by 753b in 293T cells. **b** CRISPR knockout of VHL blocks 753b-induced BCL-xL/2 degradation as shown in wild type (WT) and VHL knockout (VHL-ko) 293T cells. **c** Inhibition of CRL^VHL^neddylation with MLN4924 blocks BCL-xL/2 degradation induced by 753b in 293T cells. **d** Inhibition of proteasomes with MG132, but not that of caspases with the pan-caspase inhibitor QVD, blocks 753b-induced BCL-xL/2 degradation in 293T cells. Source data are provided as a Source data file.

A series of experiments were performed to confirm that 753b degrades BCL-xL and BCL-2 via the UPS. First, we found that pretreatment of the cells with ABT263 or VHL ligand completely blocked the degradation of BCL-xL and BCL-2 by 753b (Fig. 4a). In addition, knockout of VHL or inhibition of NEDD8-activating enzyme and proteasomes with MLN4924 and MG132, respectively, abrogated 753b-mediated degradation of BCL-xL and BCL-2, whereas caspase inhibition with the pan-caspase inhibitor QVD had no such effect (Fig. 4b–d). Furthermore, quantitative real-time PCR revealed no significant changes in the mRNA levels of *BCL2L1* and *BCL2* after 753b treatment (Supplementary Fig. 8a). Similar results were further validated in Hela cells (Supplementary Fig. 8b–e). Collectively, these results confirm that 753b degrades BCL-xL and BCL-2 in a VHL- and proteasome-dependent manner.

**Fig. 5:**
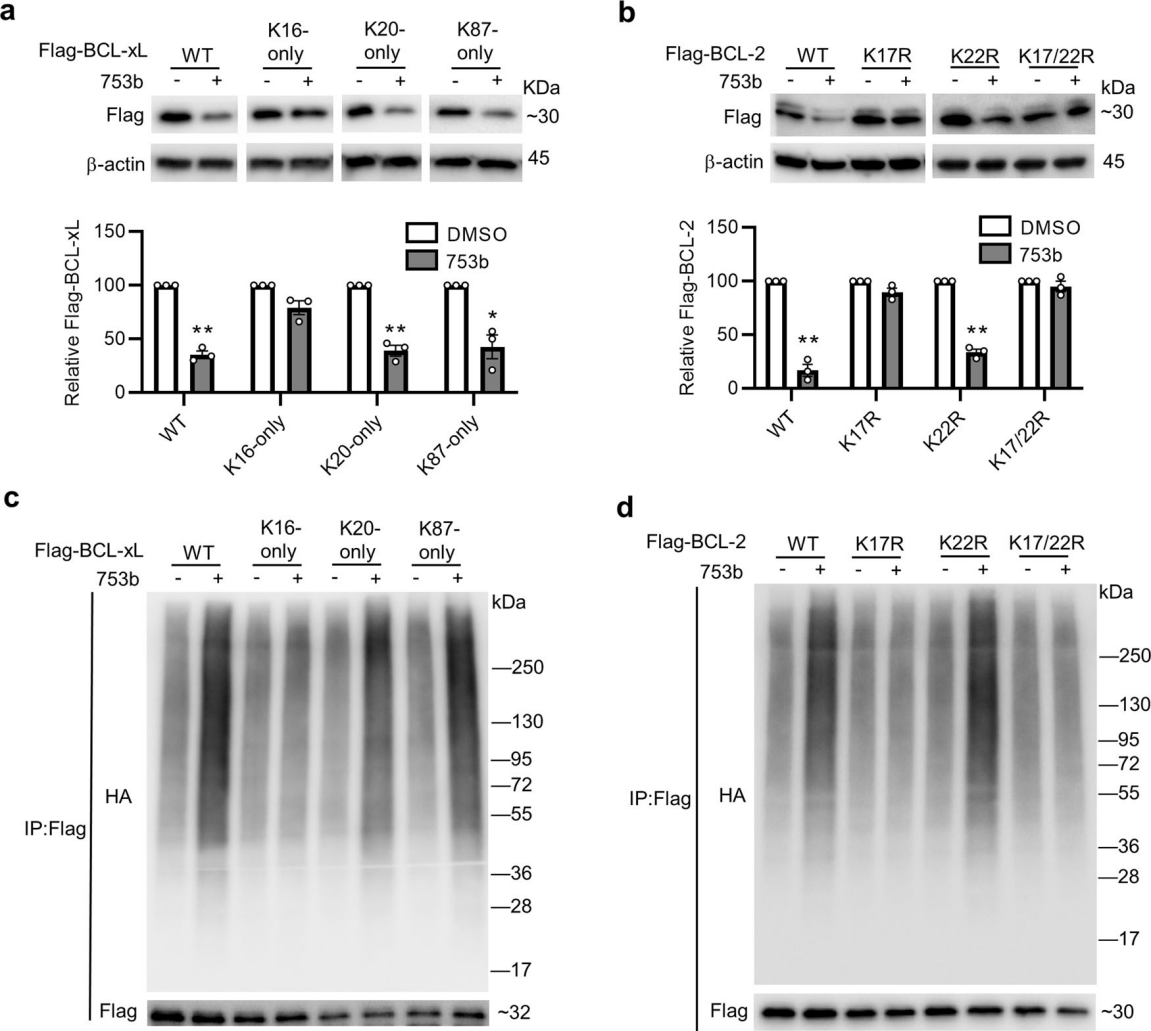
Identification of the key accessible surface lysines on BCL-xL and BCL-2. **a** 753b degrades WT BCL-xL and K20-only and K87-only BCL-xL mutants, but not K16-only BCL-xL mutant, in 293T cells. K16-only, K20-only, and K87-only represent that all the surface lysines on BCL-xL are mutated to arginines except K16, K20, and K87, respectively. Representative immunoblots of Flag-BCL-xL WT and mutants are shown in the top panel. Statistical significance was calculated with unpaired two-tailed Student’s *t*-tests comparing DMSO- to 753b-treated samples. *P < 0.05; **P < 0.01; *P* values were 0.0030, 0.0070, and 0.035 for WT, K20-only, and K87-only BCL-xL, respectively. **b** 753b degrades WT BCL**-**2 and K22R BCL-2 mutant, but not K17R and K17/22R BCL-2 mutants, in 293T cells. K17R and K22R represents that K17 and K22 on BCL-2 are respectively mutated to arginine; K17/22R means both K17 and K22 on BCL-2 are mutated to arginine. Representative immunoblots of Flag-BCL-2 WT and mutants are shown in the top panel. The quantification of the relative Flag-BCL-xL or Flag-BCL-2 protein content in the immunoblots is presented as mean values ± s.e.m. (*n* = 3 biologically independent experiments) in the bottom panel. Statistical significance was calculated with unpaired two-tailed Student’s *t*-tests comparing DMSO- to 753b-treated samples. **P < 0.01; *P* values were 0.0043 and 0.0020 for WT and K22R BCL-2 mutant, respectively. **c** 753b induces polyubiquitination of WT, K20-only, and K87-only BCL-xL, but not K16-only BCL-xL in 293 T cells. **d** 753b induces polyubiquitination of WT and K22R BCL-2, but not K17R and K17/22R BCL-2 in 293T cells. For **c**, **d** 293T cells were co-transfected as indicated with Flag-BCL-xL WT or mutants and HA-tagged ubiquitin (HA-Ub) plasmids (**c**) or Flag-BCL-2 WT or mutants and HA-Ub plasmids (**d**). After 36 h, cells were pre-treated with MG132 (10 µM) for 2 h and then treated with or without 753b (0.1 µM for BCL-xL and 1 uM for BCL-2) for 5 h. The Flag-tagged proteins were immunoprecipitated (IP) and immunoblotted with HA or Flag antibody. The input results are shown in Supplementary Fig. 9. Data are a representative of two independent experiments. Source data are provided as a Source data file.

Based on the computational model of the entire CRL^VHL^/DT2216/BCL-xL/E2-Ub/RBX1 complex (Fig. 1e), we predict that three (K16, K20, and K87) and two (K17 and K22) surface lysines on BCL-xL and BCL-2, respectively, are located in the band region (Fig. 1d, e and Supplementary Fig. 2b). K16 on BCL-xL is not available for ubiquitination due to its strong interaction with surrounding residue (Supplementary Fig. 2c) and K22 on BCL-2 is conserved with K16 on BCL-xL and also forms a salt bridge with E152 and hydrogen bonds with D102 (Supplementary Fig. 2c). Therefore, K16 (BCL-xL) and K22 (BCL-2) residues are excluded as the possible ubiquitination sites. We constructed various BCL-xL and BCL-2 mutants and showed that WT and K20- and K87-only BCL-xL mutants, but not K16-only BCL-xL mutant, can be substantially degraded by 753b (Fig. 5a). In BCL-2, the WT and K22R mutant (with K17) could be degraded by 753b; whereas K17R and K17/22R mutants (without K17) were resistant to 753b-mediated degradation (Fig. 5b). We also performed co-immunoprecipitation (co-IP) assays and confirmed that 753b-induced polyubiquitination of WT, K20-only, and K87-only BCL-xL and that of WT and K22R BCL-2 (Fig. 5c, d and Supplementary Fig. 9a, b). These results support the notion that by changing the link-out position on ABT263, we can not only generate a more potent BCL-xL degrader, but also expand the ability of the PROTAC to degrade BCL-2 by allowing the additional lysines on BCL-xL (K20) and BCL-2 (K17) to interact with the E2 enzyme for ubiquitination.

### Construction of a hypothetic E2-POI binding model

**Fig. 6:**
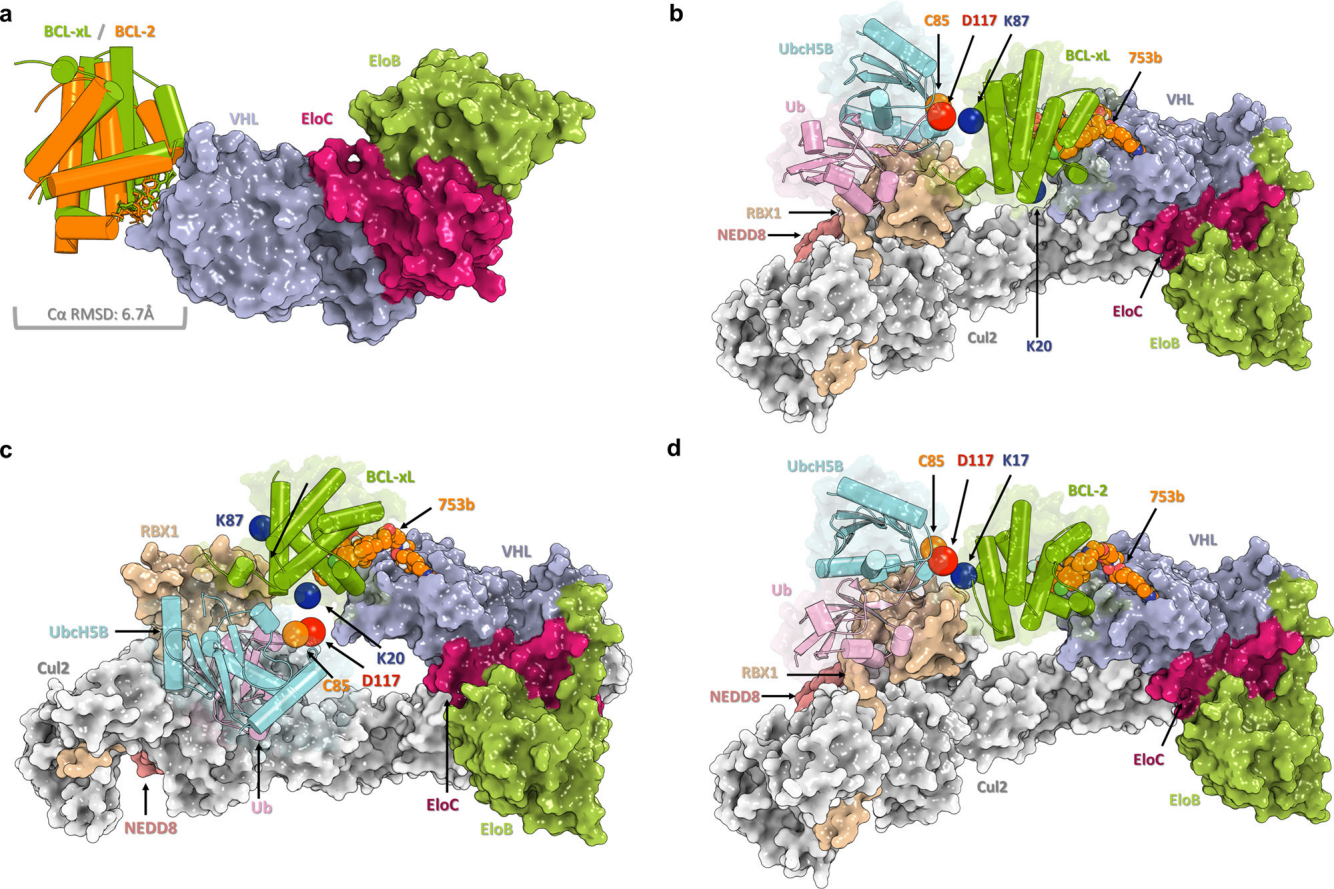
Computational models of the CRL^VHL^/753b/BCL-xL/E2-Ub/RBX1 and CRL^VHL^/753b/BCL-2/E2-Ub/RBX1 complexes. **a** The superimposition of the modeled ternary complexes of VCB/753b/BCL-xL and VCB/753b/BCL-2. The Cα RMSD of BCL-xL and BCL-2 is 6.7 Å. **b** The computational model of catalytic transition state of CRL^VHL^/753b/BCL-xL/UbcH5B-Ub/RBX1 with UbcH5B contacting with K87 of BCL-xL. The K87 of BCL-xL, C85, and D117 of UbcH5B are colored in blue, orange, and red, respectively. **c** The computational model of catalytic transition state of CRL^VHL^/753b/BCL-xL/UbcH5B-Ub/RBX1 with UbcH5B contacting with K20 of BCL-xL. **d** The computational model of catalytic transition state of CRL^VHL^/753b/BCL-2/UbcH5B-Ub/RBX1 with UbcH5B contacting with K17 of BCL-2.

The concept of a band region is a postulated model for the accessible region of E2 catalytic cysteine on a POI, with assumed flexibility of the Cullin scaffold^30,31^ as well as the RBX1 hinge loop region^32,33^ (Fig. 1e, Supplementary Fig. 2d, e and Supplementary Movies 1 and 2). To further investigate the underlying entity of this concept at the atomic level, we constructed hypothetical E2-POI binding models of the CRL^VHL^/DT2216/BCL-xL/E2-Ub/RBX1 by incorporating reported cryo-EM/crystal structures, ternary complex modeling, and normal mode analysis. Based on the ternary complexes of VCB/DT2216/BCL-xL constructed by using the PRosettaC package^34^, extensive computational sampling for the accessible conformations for the CRL^VHL^/DT2216/BCL-xL/E2-Ub/RBX1 model was performed to seek the rationale of POI-Lysine/E2-Catalytic-Cystine contacting state, namely, the ubiquitination-accessible state. The conformational sampling was limited on altering the most flexible moieties of the modeled complex, including pushing the conformation along major normal modes of Cullin scaffold as well as appropriately rotating dihedral angles of amino acids in the unfolded hinge loop region of RBX1. We confirmed that K87, R102K, and R132K on BCL-xL are located within the band region which is feasible to achieve a conformation that is close to the reaction transition state (Supplementary Fig. 10). On the contrary, K20, K157, and G186K are not in the band region and thus are not feasible to achieve a conformation that is close to the reaction transition state. This finding is consistent with our experimental data presented in Fig. 1f, which supports our hypothesis that the band region is based on E2 accessibility to the surface lysine(s) on the POI. Next, we constructed hypothetical E2-POI binding models of the CRL^VHL^/753b/BCL-xL/E2-Ub/RBX1 and CRL^VHL^/753b/BCL-2/E2-Ub/RBX1 complexes (Fig. 6) as described above. According to the ternary complexes of VCB/753b/BCL-xL and VCB/753b/BCL-2 constructed by using the PRosettaC package^34^, compound 753b induces a similar conformation of the ternary complex with both BCL-xL and BCL-2 (Fig. 6a), which is consistent with a similar strength of their modeled ternary complexes. Based on the predicted POI-Lysine/E2-Catalytic-Cystine contacting model, we confirmed that the major ubiquitination site(s) on BCL-xL and BCL-2, i.e., K87 (Fig. 6b) and K20 (Fig. 6c) of BCL-xL and K17 (Fig. 6d) of BCL-2, are located within the band region which is feasible to achieve a conformation that is close to the reaction transition state. This finding is consistent with our experimental data presented in Fig. 5a–d, demonstrating that the computational models of the entire Ub-E2 and CRL complexes have the potential to aid the development of more potent BCL-xL/2 dual degraders. Furthermore, our models suggest that by inducing different conformations of the ternary complexes (Supplementary Fig. 7), DT2216 and 753b could exhibit different potency/selectivity of PROTACs in part via altering the binding interface between VCB and BCL-xL/BCL-2 as well as the position of the surface lysine(s) on BCL-xL/BCL-2 for ubiquitination.

### 753b is a more potent antitumor agent than DT2216

**Fig. 7:**
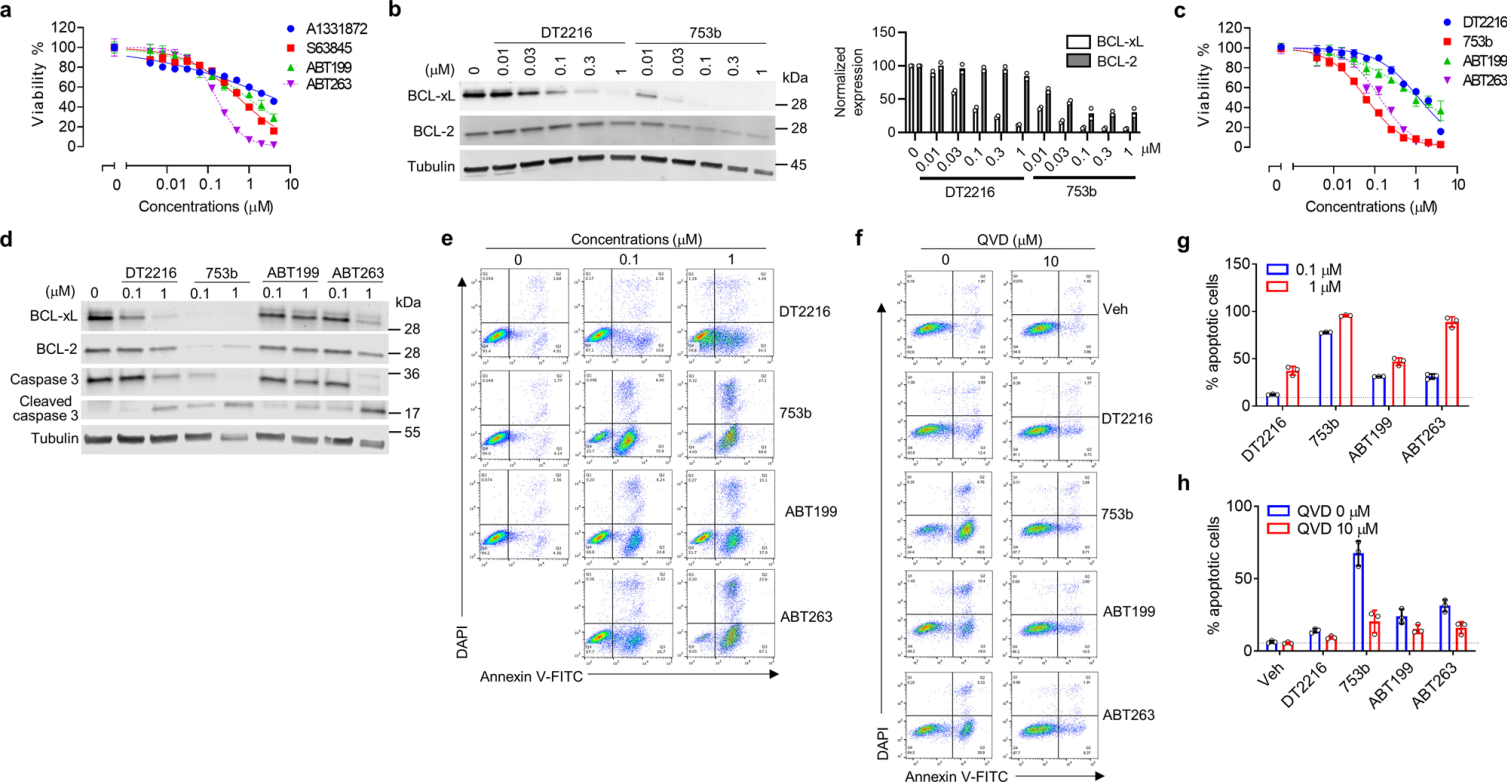
753b is a more potent anti-leukemic agent than DT2216. **a** Kasumi-1 AML cells are primarily dependent on BCL-xL/2 for survival. Viability of Kasumi-1 AML cells was measured after the cells were incubated with serially diluted ABT199 (a selective BCL-2 inhibitor), ABT263 (a dual BCL-xL/BCL-2 inhibitor), A1331872 (a selective BCL-xL inhibitor), or S63845 (a selective MCL-1 inhibitor), for 72 h. IC_50_ values for ABT199, ABT263, A1331872, and S63845 are 866.9, 156.5, 3587.0, and 527.4 nM, respectively. **b** Potencies of 753b and DT2216 in degrading BCL-xL/2 by immunoblots in Kasumi-1 cells after 16 h treatment. Tubulin was used as an equal loading control. The normalized protein content in the immunoblots is presented as mean values (*n* = 2 biologically independent experiments) in the bar graph (right panel). **c**–**h** 753b is more potent against Kasumi-1 cells than DT2216. **c** Viability of Kasumi-1 cells was measured after the cells were incubated with serially diluted DT2216, 753b, ABT199, or ABT263 for 72 h. IC_50_ values for DT2216, 753b, ABT199, and ABT263 are 1147.0, 59.64, 1130.0, and 130.9 nM, respectively. For the viability assays, the data are presented as mean ± s.d. from three replicate cell cultures in a representative experiment. Similar results were also observed in two additional independent experiments. **d** Representative immunoblot analyses of Kasumi-1 cells 16 h after treatment as indicated. Tubulin was used as an equal loading control. **e** Representative flow cytometry analyses of apoptosis using Annexin-V and DAPI staining in Kasumi-1 cells 24 h after treatment as indicated. **f** Representative flow cytometry analyses of apoptosis using Annexin-V and DAPI staining in Kasumi-1 cells after incubated with or without the pan-caspase inhibitor QVD (10 μM) for 2 h prior to being treated with DMSO (Veh) or 0.1 μM DT2216, 753b, ABT199, or ABT263 for 24 h. **g**, **h** Percentages of apoptotic cells in Kasium-1 cells analyzed by flow cytometry as shown in (**e**, **f**) are presented as mean ± s.d. from three replicate cell cultures in a representative experiment. Data are representative of two independent experiments. The dotted line represents the value from DMSO-treated cells. Source data are provided as a Source data file.

To evaluate the antitumor efficacy of 753b, we tested it in an acute myeloid leukemia (AML) cell line, Kasumi-1, which primarily depends on both BCL-xL and BCL-2 for survival because ABT263 (a BCL-xL/2 dual inhibitor) was more potent to kill the cells than A1331872^34^ (a selective BCL-xL inhibitor), ABT199^35^ (a selective BCL-2 inhibitor) or S63845^36^ (a selective MCL-1 inhibitor) (Fig. 7a). Compared with DT2216, 753b induced more potent BCL-xL degradation and could also degrade BCL-2 in Kasumi-1 cells (Fig. 7b). Further experiments also demonstrated that 753b is more potent than DT2216, ABT199 (a selective BCL-2 inhibitor) and ABT263 in killing Kasumi-1 cells by induction of apoptosis (Fig. 7c–e, g and Supplementary Fig. 11), which could be abrogated by the pretreatment of the cells with the pan-caspase inhibitor Q-VD-OPh (QVD) (Fig. 7f, h). Our previous study showed that DT2216 is less toxic to platelets than ABT263, because platelets express low levels of VHL^2^. Similar results were also observed for 753b, which failed to induce BCL-xL degradation in human platelets and was about three-fold less toxic to platelets than ABT263 (Supplementary Fig. 12).

## Discussion

For a protein to be degraded through the UPS, it has to meet at least three requirements: (1) a primary degron that can be recognized by a cognate E3 ubiquitin ligase; (2) a surface lysine(s) (second degron) that is accessible to E2 and becomes polyubiquitinated after E3 recognition, and (3) a structurally disordered segment (third degron) within or proximal to the polyubiquitinated secondary degron for the 26 S proteasome to recognize, unfold, and degrade the protein^37^. PROTACs bypass the first requirement by hijacking an E3 ligase to induce the formation of a ternary complex of the E3 ligase, PROTAC, and a POI, and then promote the proximity-induced ubiquitination and subsequent degradation of the POI. The induction of a stable ternary complex is an important determinant for a PROTAC to efficiently degrade its target^5,38^. In addition, accumulating evidence also suggests that the availability of a surface lysine on a POI accessible to an E2 for ubiquitination also plays a critical role in determining the degradability of the POI and the selectivity of a PROTAC^8–12,39^.

Using the computational modeling and mutagenesis of amino acids at the different locations on the surface of BCL-xL, we demonstrated that only the lysines located within a defined band region on the surface of BCL-xL are accessible to the catalytic site of E2 to mediate DT2216-induced BCL-xL ubiquitination and degradation. Furthermore, our data demonstrate that not only the location of the lysines but also their orientation toward the E2 component may determine if the lysines are accessible for ubiquitin transfer. We found that among two surface lysines within the band region on BCL-xL, only K87 can be ubiquitinated by DT2216 and is required for mediating DT2216-induced BCL-xL degradation, whereas K16 is dispensable for these events.

Furthermore, inspired by the aforementioned lysine accessibility findings, we developed a series of VHL-recruiting PROTACs that used the same warhead as DT2216, ABT263, but a different linker site. We expected that this linker site may change the geometry of BCL-xL-E3 interaction to expose different and/or more lysines on the protein surface and/or change the orientation of the lysines toward E2, leading to more efficient BCL-xL degradation as well as the degradation of BCL-2. Indeed, we found that the PROTACs PPC7, PPC8, and PPC9, especially PPC8, had higher potencies in degrading BCL-xL than DT2216. More importantly, unlike DT2216, they could also degrade BCL-2. We also discovered that although in cell ternary complex formation is necessary for the degradation of a POI by a PROTAC as shown previously^2^, the degradation efficacy of PROTACs with different linker lengths cannot be solely explained by the strength of the ternary complex formation induced by the different PROTACs. Moreover, we also successfully synthesized the two epimers of the most effective compound PPC8 and found that the *R*-epimer, 753b, displays even higher potency compared with the mixture compound PPC8, while the *S*-epimer, 753a, is much less potent in degrading BCL-xL and does not degrade BCL-2, compared to PPC8 and 753b. We further demonstrated that both K87 and K20 can mediate 753b-induced BCL-xL degradation, resulting in 753b being more potent than DT2216 in degrading BCL-xL, which only targets K87. Furthermore, 753b can also degrade BCL-2 in a K17-dependent manner. In addition, a hypothetical E2-POI binding model confirmed that K87 of BCL-xL and K17 of BCL-2 are located within the band region, which could achieve an E2-POI conformation that is close to the reaction transition state for E2 to transfer ubiquitin. This finding demonstrates that computational models of entire Ub-E2 and CRL complexes have the potential to aid the development of PROTACs to improve their potency as well as their selectivity. Using this strategy, we were able to broaden the specificity of ABT263-based PROTACs and generated a potent BCL-xL/2 dual degrader. Conversely, it can also be employed to improve the specificity of a PROTAC by restricting the accessibility of surface lysines on a POI to E2.

The generation of a potent BCL-xL/2 dual degrader has important clinical implications, because many types of leukemias and cancers co-depend on BCL-xL/2 for survival. Further, selection or upregulation of high BCL-xL-expressing AML cells may confer resistance to ABT199 (venetoclax), a BCL-2 inhibitor recently approved as key component of induction therapy for older AML patients^40^. Therefore, a PROTAC, such as 753b, that can degrade both BCL-xL and BCL-2, should be more effective and have a broader application for the therapy of leukemia and solid tumors than DT2216. For example, the Kasumi-1 cell line is a commonly used AML cell line that is highly resistant to cytarabine^41^, in part due to increased expression of BCL-xL and BCL-2. While these cells respond poorly to the treatment with ABT199 or DT2216 alone, they are sensitive to the treatment with ABT263, indicating that they are BCL-xL and BCL-2 co-dependent cells. Therefore, 753b induces more effective apoptosis of these AML cells than DT2216. Compared to ABT263, 753b is less toxic to platelets because platelets express lower levels of VHL than tumor cells, shown by us previously for DT2216^2^. With further modification, 753b or its optimized derivatives may be safe and effective therapeutics for treating patients with malignancies that depend on both BCL-xL an BCL-2 for survival.

However, different E3 ligases may define a different band region of surface lysines to mediate PROTAC-induced target ubiquitination and degradation. Some E3 ligases have a limited area of action and a more restricted lysine and substrate selectivity while others are broadly active over a large band region and a less defined lysine and substrate specificity^7,14^. Therefore, changing the linker site on a warhead may have a greater impact on the selectivity of a PROTAC that recruits an E3 ligase with a narrow band region of surface lysine with which to interact than an E3 ligase with a broader region of activity. For example, Lai et al. showed that both dasatinib-and bosutinib-based CRBN-recruiting PROTACs were capable of degrading c-Abl and BCR–Abl, whereas dasatinib-based VHL-recruiting PROTACs were able to degrade c-Abl but not BCR–Abl. Bosutinib-based VHL-recruiting PROTACs degrade none of them^8^. It has yet to be determined whether these differences are attributable to the different requirements of lysine accessibility for CRBN-recruiting versus VHL-recruiting PROTACs to induce target ubiquitination and degradation. In addition, lysine accessibility may have a greater impact on the degradability of a POI that has a limited number of surface lysines by a PROTAC than that of a POI with numerous lysines distributed across its surface. Nevertheless, lysine accessibility should be an important factor for consideration when developing a PROTAC to target other POIs or optimizing a PROTAC to improve its potency and specificity.

## Methods

### Chemical synthesis

Synthesis of the compound described in this paper and its intermediates is described in the Supplementary Methods and illustrated in Supplementary Fig. 13. All NMR spectra are shown in Supplementary Fig. 14–33.

### Computational modeling

#### Model of the CRL^VHL^/DT2216/BCL-xL/UbcH5B-Ub/RBX1 complex

The model was constructed by assembling several known or docked complexes with alignment of corresponding common subunits. Each part of the complex was from: Cul2 (PDB 5N4W), RBX1 (PDB 4P5O), NEDD8 (PDB 3DQV), VCB (PDB 4W9F, with VH032 analogue as ligand), UbcH5B-Ub (PDB 4V3K), BCL-xL (PDB 4QNQ, with ABT263 as ligand). It is known that NEDD8 binding would alter the conformation of winged-helix B (WHB) domain of Cul2, however, structure of Cul2-NEDD8 complex is not reported yet. With the assumption that NEDD8 binds to Cullin family with similar binding modes, the Cul2-NEDD8 binding conformation was modeled by transferring the NEDD8 from Cullin5-NEDD8 (PDB 3DQV) to Cul2 via alignment of Cullin scaffold. The WHB domain in Cul2 structure was also aligned to the corresponding WHB domain in Cullin5-NEDD8 complex. The RBX1-UbcH5B-Ub in active form (open state) was constructed by transferring the UbcH5B-Ub from structure of RNF32-UbcH5B-Ub (PDB 4V3K) to structure of RBX1-Ubc12-NEDD8 (PDB 4P5O) via alignment of UbcH5B to Ubc12. To demonstrate the flexibility of hinge loop region of RBX1, the initial RBX1 structure in Cul2 was replaced by a RBX1 structure with most extended loop region (PDB 3DQV), with synchronous movement of UbcH5B-Ub binding to RBX1. The model of VCB/VH032 complex was manually constructed based on the structure of VCB/VH032-analogue (PDB 4W9F) by adding the missing amino group in the analogue. Then the entire NEDD8-E3-E2 model (CRL^VHL^/VH032/UbcH5B-Ub/RBX1) was assembled with all the mentioned subunits based on the Cullin2 scaffold. The ternary complex conformation of BCL-xL/DT2216/VCB was constructed by Rosetta^42^-based PROTAC ternary modeling package PRosettaC^43^. The initial structures of VCB, BCL-xL, VHL032, and ABT263 were extracted from complex structures of VCB/VHL032 and BCL-xL/ABT263. The morpholine ring in ABT263 was manually modified to piperazine ring for consistency with DT2216. Openeye Toolkit^44^ was used to convert the small molecules in PDB format to SDF format. Openeye Toolkit was also used to convert DT2216 in ChemDraw format to SMILES format as a PRosettaC input. To obtain more predicted candidates of ternary complex, five independent runs were performed with “auto.py” of PRosettaC in full mode. The ternary complexes generated were clustered with density-based spatial clustering of applications with noise (DBSCAN) method^45^ and subsequently ranked based on the Rosetta interface energy. Subsequently, all the candidate ternary complexes were assembled to candidate CRL^VHL^/DT2216/BCL-xL/UbcH5B-Ub/RBX1 complexes by alignment of VCB part. K87 of BCL-xL is the known ubiquitination site for E2 modification, so K87 should be reachable by C85 of UbcH5B, which is the catalytic residue of E2. The distance between VHL and UbcH5B is about 50 Å (distance between VH032 linking site and C85 of UbcH5B), which is expected to be the maximal distance between POI and UbcH5B. So, the candidate complex with lowest energy as well as meeting the criteria that distance between K87 (ζ-N) atom of BCL-xL and C85 (γ-S) atom of UbcH5B less than 50 Å was selected as the model of the CRL^VHL^/DT2216/BCL-xL/UbcH5B-Ub/RBX1 complex. Hereinafter, all the structural alignment operations were performed by PyMol^46^, and the default parameters were used for all software/commands/functions unless otherwise noted.

#### Model of CRL^VHL^/753b/BCL-xL/UbcH5B-Ub/RBX1 in an E2/POI contacting state

Initial model of CRL^VHL^/753b/BCL-xL/UbcH5B-Ub/RBX1 complex was constructed by following the same protocol of constructing the CRL^VHL^/DT2216/BCL-xL/UbcH5B-Ub/RBX1 complex, using 753b instead of DT2216 as the ligand for PRosettaC input. Both K87 and K20 of BCL-xL were far away from the catalytic residue C85 of UbcH5B in the initial model, which suggested that the flexibility of E3 should be considered in demonstrating the quasi-reaction-ready state of ubiquitination. Based on the initial model, normal mode analysis was used to explore possible conformation perturbations of the complex. The top five major normal modes of complex was calculated with elastic network model sever DynOmics^47^, and 28 frame of atomic coordination of perturbated conformations for each major normal mode were generated by anisotropic network model (ANM) within root-mean-square deviation of 10 Å using the dynamics module “Molecular Motions—Animations” to generate the ANM-Driven conformers, while all other parameters were kept at default. A total of 140 perturbated conformations were generated in this step. Then the conformational flexibility of unfolded loop region of RBX1 (residue #36–40) were sampled by using PyMol^46^ script to randomly change the ψ and φ dihedral angels of these residues for 10,000 times using PyMol command “set_dihedral” with random angles. The UbcH5B and Ub binding to RBX1 were synchronously moved with RBX1. If the catalytic C85 of UbcH5B could not reach K87 or K20 of BCL-xL within any perturbated E3 conformations, it could be assumed that either K87 or K20 is not accessible for ubiquitination and vice versa. With removing crashed structures using the select function to detect crashed atoms in PyMol and calculating the distance between C85 of UbcH5B and K87/K20 of BCL-xL using the distance function in PyMol in all these 1,400,000 perturbated E3 conformations, we found that both K87 and K20 could be reached by C85 in multiple conformations with the contacting threshold of 8 Å. Then PyMol^46^ was used to reduce the distance to about 3 Å with manually fine-tuning for the loop-region of RBX1.

#### Model of CRL^VHL^/753b/BCL-2/UbcH5B-Ub/RBX1 in an E2/POI contacting state

The model for CRL^VHL^/753b/BCL-2/UbcH5B-Ub/RBX1 complex in an E2/POI contacting state was constructed by following the same protocol of constructing of the CRL^VHL^/753b/BCL-xL/UbcH5B-Ub/RBX1 complex in E2/POI contacting state, using the X-ray crystal structure of BCL-2/ABT263 complex (PDB:4LVT) instead of 753b. Besides, the ubiquitination accessibility of E2/BCL-2 was calculated by distance between C85 of UbcH5B and K17 of BCL-2.

#### Model of CRL^VHL^/DT2216/BCL-xL/UbcH5B-Ub/RBX1 in an E2/POI contacting state

The model for CRL^VHL^/DT2216/BCL-2/UbcH5B-Ub/RBX1 complex in an E2/POI contacting state was constructed by following the same protocol of constructing the CRL^VHL^/DT2216/BCL-xL/UbcH5B-Ub/RBX1 complex in E2/POI contacting state, using the constructed CRL^VHL^/DT2216/BCL-2/UbcH5B-Ub/RBX1 complex as a starting structure. The ubiquitination accessibility of E2/BCL-2 was calculated by the distance between C85 of UbcH5B and K87/K20/K157/G186K/R102K/R132K of BCL-xL. The conformation of G186K, R102K, and R132K were generated by the Mutagenesis function of PyMol. The model for E2 contacting K87/R132K of BCL-xL could be constructed with the same protocol described in the previous section. However, the E2 is not accessible for K20/K157/G186K/R102K of BCL-xL with sampling the five major normal modes alone. In this case, we explored the perturbation of conformations with the combination of the first two major normal modes, which are the most representative conformational motion modes of the complex. In the first step, the 5, 10, 15, 20, and 25 frames from the second major normal mode were selected as the starting structures for the next cycle of normal mode analysis. Elastic network model sever DynOmics was used to generate the first major normal mode for the five selected structures. Then the remaining steps followed the protocol for constructing the K87/R132K contacting model. Only R102K contacting model could meet the UbcH5B-lysine distance threshold. That is, E2 is still not accessible for K20/K157/G186K, which suggests that these residues are not potential ubiquitination sites.

#### Root-mean-square deviation (RMSD) between BCL-xL and BCL-2

It is not feasible to calculate the RMSD of two different proteins, however, BCL-xL and BCL-2 are homology proteins. Therefore, the RMSD between BCL-xL and BCL-2 is estimated by RMSD between BCL-xL and its duplicate aligned to BCL-2.

### Cell lines and cell culture

HEK293T (293T, Cat. No. ACS-4500), Hela (Cat. No. CCL-2), Kasumi-1 (Cat. No. CRL-2724) cells were recently purchased from American Type Culture Collection (ATCC, Manassas, VA, USA). 293T and Hela cells were cultured in complete Dulbecco’s modified Eagle medium (DMEM, Cat. No. 12430054, Thermo Fisher Scientific) supplemented with 10% (vol/vol) heat-inactivated fetal bovine serum (FBS, Cat. No. S11150H, Atlanta Biologicals, Flowery Branch, GA, USA), 100 U/ml penicillin and 100 µg/ml streptomycin (penicillin–streptomycin, Cat. No. 15140122, Thermo Fisher Scientific). Kasumi-1 cells were cultured in RPMI 1640 medium (Cat. No. 22400–089, Thermo Fisher Scientific) supplemented with 10% FBS, 100 U/ml penicillin and 100 µg/ml streptomycin. All cells were maintained in a humidified incubator at 37 °C and 5% CO_2._ Human platelet-rich plasma (PRP) was purchased from Zenbio (Durham, NC) (cat. no. SER-PRP-SDS).

### Immunoblotting

Cells were collected, washed once with ice-cold phosphate-buffered saline, pH 7.2 (PBS, cat. no. 20012027; Thermo Fisher Scientific) and lysed in RIPA lysis buffer (Cat. No. BP-115DG, Boston Bio Products, Ashland, MA, USA) supplemented with protease and phosphatase inhibitor cocktails (Cat. No. PPC1010, Sigma-Aldrich, St. Louis, MO, USA) through sonication. The samples were centrifuged at 14,000 r.p.m. for 5 min and the supernatants were transferred into a new tube. The protein concentration in the supernatants was determined using the Pierce BCA protein Assay kit (cat. no. 23225, Thermo Fisher Scientific). The protein concentration was normalized, and the samples were reduced in 4× Laemmli’s SDS-sample buffer (cat. no. BP-110R, Boston Bio Products) and denatured at 95 °C for 5 min. An equal amount of protein samples (20–40 μg per lane) were resolved using precast 4–20% Tris-glycine gels (Mini-PROTEAN TGX, cat. no. 456–1094, Bio-Rad), and resolved proteins were transferred onto 0.2-µm pore size polyvinylidene difluoride (PVDF) blotting membranes (cat. no. LC2002, Thermo Fisher Scientific) using mini trans-blot electrophoretic transfer cell (Bio-Rad). The membranes were blocked with non-fat dry milk (5% wt/vol) in 1× Tris-buffered saline-Tween-20 (TBST, cat. no. J77500, Affymetrix) for 1 h at room temperature, and were subsequently probed with primary antibodies at a predetermined optimal concentration in non-fat dry milk (5% wt/vol in TBST) overnight at 4 °C. The membranes were washed three times (5 min each) in TBST and then incubated with horse radish peroxidase (HRP)-conjugated secondary antibodies for 1 h at room temperature. Following sufficient washing with TBST, the membranes were exposed with chemiluminescent HRP substrate (cat. no. WBKLS0500, MilliporeSigma), and the signal was detected using the ChemiDoc MP Imaging System (Bio-Rad) and quantified using the ImageJ (v1.53a) software from NIH.

Antibodies purchased from Cell Signaling Technologies (CST) and the dilutions are as follows: BCL-xL (Cat. No. 2762S, 1:1000), BCL-2 (Cat. No. 2870S, 1:1000), VHL (Cat. No. 68547S, 1:1000), β-Tubulin (Cat. No. 2146S, 1:1000), Caspase-3 (Cat. No. 9662S, 1:1000), Cleaved Caspase-3 (Cat. No. 9664S, 1:1000), Flag-tag (Cat. No. 14793S, 1:1000), HA-tag (Cat. No. 3724S, 1:1000). β-actin antibody was purchased from MP Biomedicals (Cat. No. 8691001, 1:20,000).

### Viability assays

For the viability assays in 293T, Hela, Kasumi-1 cells, the cells in complete cell culture medium were seeded in 96-well plates (100 µl per well) at the optimized densities (50,000–100,000 suspension cells, 3000–5000 adherent cells). Suspension cells were treated 30 min after seeding, whereas adherent cells were allowed to adhere overnight and were then treated. Compound treatments were prepared in complete cell culture medium and 100 µl of 2× treatment-containing medium were added to each well. Complete cell culture medium without treatment was added in control wells, and wells containing medium without cells served as background. The outer wells of the 96-well plate were not used for treatment and were filled with 250 µl of PBS to reduce evaporation of medium from inner wells. Each compound and/or combination was tested at nine different concentrations with three replicates. The cell viability was measured after 72 h by tetrazolium-based MTS assay. MTS reagent (2 mg ml^–1^ stock; cat. no. G1111, Promega) was freshly supplemented with phenazine methosulfate (PMS, 0.92 mg ml^–1^ stock, cat. no. P9625, Sigma-Aldrich) in 20:1 ratio, and 20 µl of this mixture was added to each control and treatment well. The cells were incubated for 4 h at 37 °C and 5% CO_2_, and then the absorbance was recorded at 490 nm using Biotek’s Synergy Neo2 multimode plate reader (Biotek). The average absorbance value of background wells was subtracted from absorbance value of each control and treatment well, and percent cell viability ((At/A0) × 100)) was determined in each treatment well, where At is the absorbance value of the treatment well and A0 is the average absorbance value of control wells after background subtraction. The data were expressed as average percentage cell viability and fitted in non-linear regression curves using GraphPad Prism 7 (GraphPad Software, La Jolla, CA, USA).

For the viability assays in human platelets, human platelet-rich plasma (PRP) was purchased from Zenbio (cat. no. SER-PRP-SDS). PRP was used for experiments immediately after delivery. PRP was transferred into 50-ml polypropylene tubes, each containing 5 ml acid citrate buffer (cat. no. sc-214744, Santa Cruz Biotechnology). To prevent clotting, prostaglandin E1 (PGE1, cat. no. sc-201223A, Santa Cruz Biotechnology) and apyrase (cat. no. A6237, Sigma-Aldrich) were added to final concentrations of 1 µM and 0.2 units per ml, respectively. After gently mixing the solution, platelets were pelleted by centrifugation at 1200 × *g* for 10 min. Pelleted platelets were gently washed without disruption in 2 ml HEPES-buffered Tyrode’s solution (10 mM HEPES, 135 mM NaCl, 2.8 mM KCl, 1 mM MgCl_2_, 2 mM CaCl_2_, 12 mM NaHCO_3_, 0.4 mM NaH_2_PO_4_, 0.25% BSA and 5.5 mM glucose, pH 7.4; cat. no. PY-921WB, Boston BioProducts) containing 1 µM PGE1 and 0.2 units ml^–1^ apyrase. After washing, pellets were slowly resuspended in 10 ml HEPES-buffered Tyrode’s solution containing 1 µM PGE1 and 0.2 units ml–1 apyrase. The number of platelets was counted using a HEMAVET 950FS hematology analyzer (Drew Scientific, Inc.). For viability assays, platelet number was adjusted to 2 × 10^8^ per ml in HEPES-buffered Tyrode’s solution containing 1 µM PGE1, 0.2 units ml^–1^ apyrase and 10% FBS. Each treatment was given in 2 ml platelet suspension in 15-ml polypropylene tubes for 72 h. The tubes were placed on a rotating platform at room temperature for the duration of treatment. After 72 h treatment, 200 µl of untreated or treated platelets were plated in each well of 96-well plates, and the viability was measured by MTS assay as described above.

### Plasmid construction for mutational analysis

Plasmids were purified on miniprep columns according to the manufacturer’s protocol (Cat. No. 27106 Qiagen, Germantown, MD, USA). The construction of Flag-BCL-xL (pDL2009) and Flag-BCL-xL-K-ko (pDL2129) were described previously^2^. Flag-BCL-2 was a gift from Clark Distelhorst (Addgene plasmid # 18003, Watertown, MA, USA). Plasmids Flag-BCL-xL-K16-only, Flag-BCL-xL-K20-only, Flag-BCL-xL-K157-only, Flag-BCL-xL-K-ko-R34K, Flag-BCL-xL-K-ko-R102K, Flag-BCL-xL-K-ko-R132K, Flag-BCL-xL-K-ko-G186K, Flag-BCL-2-K17R, Flag-BCL-2-K22R, and Flag-BCL-2-K17/22R were constructed by using Q5® Site-Directed Mutagenesis Kit (Cat. No. E0554S, NEB, Ipswich, MA, USA) and the following primer sets. Flag-BCL-xL-K-ko (pDL2129) or Flag-BCL-2 was used as the template. Flag-BCL-xL-K16-only: forward (5′-CTCTCCTACAaGCTTTCCCAG-3′) and reverse (5′-AAAGTCAACCACCAGCTC-3′); Flag-BCL-xL-K20-only: forward (5′-CTTTCCCAGAaAGGATACAGC-3′) and reverse (5′-CCTGTAGGAGAGAAAGTC-3′); Flag-BCL-xL-K157-only: forward (5′-AGCGTAGACAaGGAGATGCAGG-3′) and reverse (5′-TTCCACGCACAGTGCCCC-3′); Flag-BCL-xL-R34K: forward (5′-GAAGAGAACAaGACTGAGGCC-3′) and reverse (5′-CACATCACTAAACTGACTCC-3′); Flag-BCL-xL-R102K: forward (5′-ACTGCGGTACaaGCGGGCATTC-3′) and reverse (5′-TCAAACTCGTCGCCTGCC-3′); Flag-BCL-xL-R132K: forward (5′-TGAACTCTTCaaGGATGGGGTAAAC-3′) and reverse (5′-TTCACTACCTGTTCAAAG-3′); Flag-BCL-xL-G186K: forward (5′-CCAGGAGAACaaCGGCTGGGATAC-3′) and reverse (5′-ATCCAAGGCTCTAGGTGG-3′); Flag-BCL-2-K17R: forward (5′-ATAGTGATGAgGTACATCCATTATAAGC-3′) and reverse (5′-CTCCCGGTTATCGTACCC-3′); Flag-BCL-2-K22R: forward (5′-ATCCATTATAgGCTGTCGCAG-3′) and reverse (5′-GTACTTCATCACTATCTCC-3′); Flag-BCL-2-K17/22R: forward (5′-cattatagGCTGTCGCAGAGGGGCTA-3′) and reverse (5′-gatgtaccTCATCACTATCTCCCGGTTATCG-3′). DNA sequences in all these plasmids were authenticated by automatic sequencing. 293T cells were transfected with different plasmids using Lipofectamine 2000 reagent (Cat. No. 11668019, Thermo Fisher Scientific) and 36 h later, the cells were treated with indicated compound for 6 h before being analyzed. The cells were harvested using RIPA lysis buffer containing protease and phosphatase inhibitor as mentioned above. The primers used in this study are also listed in Supplementary Table 1.

### Reverse transcription and quantitative PCR (RT-qPCR)

Total RNA was extracted using RNeasy Plus Mini Kit (Qiagen, Cat. No. 74134). Reverse transcription was carried out by using High Capacity cDNA Reverse Transcription Kit (Invitrogen). Quantitative PCR (qPCR) was performed using an QuantStudio™ 5 Real-Time PCR System with SYBR Green. The PCR reactions were set up in a 96-well optical plate by adding the following reagents into each well: 2 μl of cDNA, 10 μl of SYBR Green PCR Master Mix (Applied Biosystems, Foster City, CA, USA); the final concentrations of primers were 0.5 μmol/L in a final volume of 20 μl. The PCR amplification protocol was initiated at 50 °C for 2 min followed by 3 min at 95 °C and 40 PCR cycles consisting of 5 s at 95 °C followed by 60 °C for 30 s. All samples were tested with the reference gene GAPDH for data normalization to correct for variations in RNA quality and quantity. The specificity of amplification of targets with high Ct values was confirmed by analysis of the temperature dissociation curves. Primers used for measuring gene transcriptional level: *GAPDH* primers (forward 5′-GACCACTTTGTCAAGCTCATTTC-3′ and reverse 5′-CTCTCTTCCTCTTGTGCTCTTG-3′) were described previously^48^. *BCL2L1* primers are forward 5′-GAGCTGGTGGTTGACTTTCTC-3′ and reverse 5′-TCCATCTCCGATTCAGTCCCT-3′; *BCL2* primers are forward 5′-GGTGGGGTCATGTGTGTGG-3′ and reverse5′-CGGTTCAGGTACTCAGTCATCC-3′.

### AlphaScreen for the determination of PROTAC and BCL-xL/BCL-2 binary binding affinity

AlphaScreen competitive binding assays were used to evaluate the binding affinities of 753a and 753b toward BCL-xL/BCL-2. The reagents were diluted in a buffer containing 250 mM HEPES pH 7.5, 1 M NaCl, 1% BSA, and 0.05% Tween-80. His-tagged BCL-xL (0.5 nM, Cat. No. SRP0187, Sigma-Aldrich) or BCL-2 (1 nM, Cat. No. SRP0186, Sigma-Aldrich) were incubated with serial diluted 753a or 753b and 2 nM biotin-tagged BAD (biotin-LWAAQRYGRELRRMSDEFEGSFKGL amino-terminal, AnaSpec, for BCL-xL) or 10 nM BIM peptides (biotin-MRPEIWIAQELRRIGDEFNA N-terminal, AnaSpec, for BCL-2) to a final volume of 40 µl in a 96-well PCR plate. DT2216 and ABT263 were also included in the assays. After incubation for 2 h, 5 µl 6× His-acceptor beads (Cat. No. AL128M, PerkinElmer) were added to each well at 20 µg/ml final concentration and incubated for 1 h. Then 5 µl streptavidin-donor beads were added (Cat. No. 6760002, PerkinElmer) to each well at 20 µg/ml final concentration and incubated for 45 min. At the end of the incubation period, 17 µl of each sample was transferred in adjacent wells of 384-well proxy plate (Cat. No. 6008280, PerkinElmer). The plate was scanned using the Alpha program on Biotek’s Synergy Neo2 multimode plate reader. The inhibition constant (Ki) was calculated using the non-linear regression, one site, competitive binding, Fit Ki function on GraphPad Prism 9 software, based on experimentally determined dissociation constant (Kd) for each protein–peptide pair.

### NanoBRET ternary complex formation assay

Plasmids HaloTag-VHL (Cat. No. CS1679C155) and CMV HiBit (Cat. No. CS1956B03) were purchased from Promega. HiBit-BCL-xL and HiBit-BCL-2 were constructed previously^2^. The two mutants (HiBit-BCL-xL-K157-only and HiBit-BCL-xL-K-ko) were constructed through Gibson assembly method. The primer pair (5′-TGGCTCGAGCGGTGGGAATTCTGGTATGTCTCAGAGCAACCGGGAGCTGGTG-3′ and 5′-TCTTCCGCTAGCTCCACCGGATCCTCCTCATcTCCGACTGAAGAGTGAGCC-3′) and the template Flag-BCL-xL-K157-only or Flag-BCL-xL-K-ko (pDL2129)^2^ were used to clone the BCL-xL-K157-only-containing and BCL-xL-K-ko-containing fragments, respectively. The primer pair (5′-GGCTCACTCTTCAGTCGGAgATGAGGAGGATCCGGTGGAGCTAGCGGAAGA-3′ and 5′-CACCAGCTCCCGGTTGCTCTGAGACATACCAGAATTCCCACCGCTCGAGCCA-3′) and pBit3.1-N (Cat. No. N2361, Promega) were used to clone the vector-containing fragment. The PCR fragments were assembled using NEBuilder® HiFi DNA Assembly Master Mix (Cat. No. E2621, NEB, Ipswich, MA, USA). DNA sequences in all these plasmids were authenticated by automatic sequencing. 293 T cells (8 × 10^5^) were transfected with Lipofectamine (Life Technologies) and 1 μg HaloTag-VHL, 10 ng HiBit-BCL-xL or HiBit-BCL-xL-K157-only or HiBit-BCL-xL-K-ko and 10 ng LgBit or 1 μg HaloTag-VHL, 10 ng HiBit-BCL-2 and 10 ng LgBit. After 24 h, 2 × 10^4^ transfected cells were seeded into white 96-well tissue culture plates in Gibco™ Opti-MEM™ I Reduced Serum Medium, No Phenol Red (Cat. No. 11-058-021, Fisher) containing 4% FBS with or without HaloTag NanoBRET 618 Ligand (Cat. No. PRN1662, Promega) and incubated overnight at 37 °C, 5% CO_2_. The following day, serial diluted compounds were added into the medium and plates were incubated at 37 °C, 5% CO_2_, for 6 h. After treatment, NanoBRET Nano-Glo Substrate (cat. no. N1662, Promega) was added into the medium, and the contents were mixed by shaking the plate for 30 s before measuring donor and acceptor signals on Biotek plate reader. Dual-filtered luminescence was collected with a 450/50 nm bandpass filter (donor, NanoBiT-BCL-XL protein or NanoBiT-BCL-2 protein) and a 610-nm longpass filter (acceptor, HaloTag NanoBRET ligand) using an integration time of 0.5 s. mBRET ratios were calculated following the NanoBRET™ Nano-Glo® Detection System (Cat. No. N1662, Promega).

### Plasmids for purification of recombinant VCB protein complex

The plasmid co-expresses Elongin B and Elongin C (AA 17-112) (EloB/C) was a gift from Nicola Burgess-Brown (Addgene plasmid # 110274). pGEX-6P-1-VHL(54-213) (pDL2200) was constructed through Gibson assembly method. The primer pair (5′-CTGGAAGTTCTGTTCCAGGGGCCCATGGAGGCCGGGCGGCCGCGGC-3′ and 5′-ATCGTCAGTCAGTCACGATGCGGCTCAATCTCCCATCCGTTGATGTGCAATG-3′) and HA-VHL (a gift from William Kaelin, Addgene plasmid # 19999) were used to clone the VHL(54-213)-containing fragment. The primer pair (5′-CATTGCACATCAACGGATGGGAGATTGAGCCGCATCGTGACTGACTGACGAT-3′ and 5′-GCCGCGGCCGCCCGGCCTCCATGGGCCCCTGGAACAGAACTTCCAG-3′) and pGEX-6P-1 Brd4 full-length (a gift from Peter Howley, Addgene plasmid # 14447) were used to clone the vector-containing fragment. The PCR fragments were assembled using NEBuilder® HiFi DNA Assembly Master Mix (Cat. No. E2621, NEB, Ipswich, MA, USA). DNA sequences in all these plasmids were authenticated by automatic sequencing.

### Expression and purification of VCB complex

To purify glutathione-S-transferase-tagged-VHL-EloC-EloB (GST-VCB) protein complex, pGEX-6P-1-VHL (54-213) and EloB/C co-transformed cells were grown in LB broth at 37 °C with shaking until the optical density at 600 nm reached 0.6–0.7. Isopropyl-β-D-thiogalactopyranoside (1 mM) was added to induce protein expression overnight at 16 °C. Cell pellet was resuspended in lysis buffer [50 mM Tris-Hcl (pH 7.5), 150 mM NaCl, 1 mM EDTA, 0.05% NP-40, 1 mg/ul Lysozyme, 1× protease inhibitor, and 1 mM DTT] and lysed using sonication. The lysate was cleared by centrifugation at 20,000 × *g* for 30 min at 4 °C and purified by using Glutathione Sepharose™ 4B Media (Cat No. 45-000-139, Cytiva). After elution with glutathione, the proteins were concentrated by using Pierce Protein Concentrators PES, 30 K MWCO (Cat No. 88502, ThermoFisher) and then dialyzed into the buffer containing 50 mM HEPES, 150 mM NaCl, and 0.1 mM EDTA (pH 7.5).

### AlphaLISA assay for evaluating the ternary complex formation

AlphaLISA assay was used to evaluate the in vitro ternary complex formation between target proteins (BCL-xL or BCL-2), PROTAC and E3 ligase (VCB complex). In each well, 5 nM His-tagged BCL-xL or BCL-2 proteins and 5 nM GST-VCB complex was incubated with varying concentrations of compounds in fourfold serial dilutions, to a final volume of 40 µl in a 96-well PCR plate. After incubation at room temperature for 30 min, 5 µl alpha glutathione-donor beads (Cat. No. 6765300, PerkinElmer) were added to each well (20 µg/ml final concentration) and incubated for another 15 min. Thereafter, 5 µL 6× His-acceptor beads were added to each well (20 µg/ml final concentration) and incubated for an additional 45 min at room temperature. Thereafter, 17 µl of each sample was transferred in adjacent wells of a 384-well proxy plate, and the plate was scanned using the Alpha program on Biotek’s Synergy Neo2 multimode plate reader. The data were expressed as average AlphaLISA signal and plotted against different concentrations of compounds using GraphPad Prism 9 software.

### VHL knockout by CRISPR/Cas9 genomic editing

To deplete VHL, the sgRNAs targeting human *VHL* were designed and cloned into lentiCRISPR v2 vector (a gift from Feng Zhang; Addgene plasmid # 52961). Packaging 293T cells were transfected with *VHL* sgRNAs or negative control (non-targeting sgRNA-NC^49^) and helper vectors (pMD2.G and psPAX2; Addgene plasmid # 12259 and 12260) using Lipofectamine 2000 reagent (Cat. No. 11668019, Life Technologies). Medium containing lentiviral particles and 8 μg/mL polybrene (Sigma-Aldrich) was used to infect 293 T cells. Infected cells were selected in medium containing 2 μg/mL puromycin. The target guide sequences are as follows: forward (5’-CACCGTGTCCGTCAACATTGAGAGA-3’) and reverse (5’-AAACTCTCTCAATGTTGACGGACAC-3’). Single clones were selected by serial dilution.

### Polyubiquitination assay

293T cells were cotransfected with wild type or mutated Flag-BCL-xL and HA-ubiquitin (a gift from T. Dawson, Addgene, Plasmid no. 17608) or wild type or mutated Flag-BCL-2 and HA-ubiquitin for 40 h, and then pretreated with 10 μM MG132 for 1 h to prevent PROTAC-induced target protein degradation before treated with DMSO or 753b for 5 h. Proteins were extracted by using IP lysis buffer (Cat. No. 87788, Thermal Fisher Scientific) and IP was performed using Anti-FLAG M2 Magnetic Beads (Cat. No. M8823, Sigma-Aldrich) according to the manufacturer’s protocol. Anti-FLAG M2 Magnetic Beads were washed with 1× TBS three times and then added to protein samples, and the mixture was incubated at 4 °C with rotation overnight. The magnetic beads were collected and then washed three times with 1× TBS. Immunoprecipitated samples were eluted with 2 × SDS-sample buffer and boiled 5 min at 95 °C.

### Flow cytometry

Kasumi-1 cells were plated in 12 well plates at 0.2 × 10^6^ cells/well and treated with DMSO, 0.1 or 1 μM DT2216, 753b, ABT-199, or ABT263 for 24 h. For the apoptosis inhibition experiment, Kasumi-1 cells were cultured with or without the pan-caspase inhibitor QVD (10 μM, Cat. No. SML0063, Sigma-Aldrich) for 2 h prior to being treated with DMSO (Veh) or 0.1 μM DT2216, 753b, ABT199, or ABT263 for 24 h. After treatment, cells were stained with Annexin V-FLUOS (1: 200, Cat. No. 11828681001, Roche, Little Falls, NJ, USA) at room temperature for 15 min, washed with PBS and then added DAPI (0.2 μg/mL, Cat. No. D1306, Invitrogen, Waltham, MA, USA) for apoptosis flow cytometry on Gallios (Beckman, Indianapolis, IN, USA).

### Statistics and reproducibility

All experiments were performed 2 or more independent times with similar results.

### Reporting summary

Further information on research design is available in the Nature Research Reporting Summary linked to this article.

## Supplementary information

Supplementary Information

Description of Additional Supplementary Files

Supplementary Data 1

Supplementary Movie 1

Supplementary Movie 2

Reporting Summary

## Data Availability

The structural models generated in this study are provided in Supplementary Data 1. All the input files used for generating structural model with PRosettaC could be accessed from GitHub repository: https://github.com/lezephyr1988/BCLxl-BCL2. The crystal structures used in this study are available in the PDB under accession codes 3DQV, 4LVT, 4P5O, 4QNQ, 4V3K, 4W9F, and 5N4W. Source data are provided with this paper.

## Source data

Source data

## Code availability

The PyMol script (including example and description) for automatically sampling dihedral angels to generate the E2/POI contacting model is available in GitHub at https://github.com/lezephyr1988/sample_ternary_dihedral.
