# Supplementary material for: Development of a BCL-xL and BCL-2 dual degrader with improved anti-leukemic activity,
Source: Nat Commun. 2021 Nov 25;12:6896. doi: 10.1038/s41467-021-27210-x (PMC8617031; doi:10.1038/s41467-021-27210-x)
Supplement: Supplementary file 1 — Supplementary Information [file 41467_2021_27210_MOESM1_ESM.docx]

**­­­­­Supplementary Information**

**Development of a BCL-xL and BCL-2 dual degrader with improved anti-leukemic activity**

Lv D et al.

| **Supplementary Item** | **Title** |
| --- | --- |
| Supplementary Fig. 1 | CRBN-based PROTAC XZ739 can form a ternary complex with both BCL-xL and BCL-2 but only degrades BCL-xL. |
| Supplementary Fig. 2 | Comparison of lysine distributions on BCL-xL and BCL-2 and the normal modes of the CRLVHL/DT2216/BCL-xL/E2-Ub/RBX1 complex. |
| Supplementary Fig. 3 | Lysine to arginine mutations on BCL-xL do not affect the formation of the BCL-xL-DT2216-VHL ternary complex. |
| Supplementary Fig. 4 | BCL-xL/2 degradation induced by the new BCL-xL and/or BCL-2 PROTACs with different linker lengths. |
| Supplementary Fig. 5 | 293T and Hela cells are not BCL-xL/2 dependent. |
| Supplementary Fig. 6 | BCL-xL and BCL-2 binary binding affinity determined by AlphaScreen assay. |
| Supplementary Fig. 7 | The modelled ternary complexes. |
| Supplementary Fig. 8 | Validation of the mechanism of 753b. |
| Supplementary Fig. 9 | Polyubiquitination of BCL-xL and BCL-2 induced by 753b. |
| Supplementary Fig. 10 | Computational models of the CRLVHL/DT2216/BCL-xL/E2-Ub/RBX1 for UbcH5B-K87, UbcH5B-R102K and UbcH5B-R132K contacting status. |
| Supplementary Fig. 11 | Gating strategy to determine the percentage of apoptotic cells in Kasium-1 cells by flow cytometry for Fig. 7. |
| Supplementary Fig. 12 | Evaluation of 753b in human platelets. |
| Supplementary Fig. 13 | The synthetic scheme of the PROTACs |
| Supplementary Fig. 14 | ^1^H NMR of **2/2b/2a** |
| Supplementary Fig. 15 | ^13^C NMR of **2/2b/2a** |
| Supplementary Fig. 16 | ^1^H NMR of **3/3b/3a** |
| Supplementary Fig. 17 | ^13^C NMR of **3/3b/3a** |
| Supplementary Fig. 18 | ^1^H NMR of **4/4b/4a** |
| Supplementary Fig. 19 | ^13^C NMR of **4/4b/4a** |
| Supplementary Fig. 20 | ^1^H NMR of **6** |
| Supplementary Fig. 21 | ^1^H NMR of **6b** |
| Supplementary Fig. 22 | ^13^C NMR of **6b** |
| Supplementary Fig. 23 | ^1^H NMR of **6a** |
| Supplementary Fig. 24 | ^1^H NMR of **PPC5** |
| Supplementary Fig. 25 | ^1^H NMR of **PPC6** |
| Supplementary Fig. 26 | ^1^H NMR of **PPC7** |
| Supplementary Fig. 27 | ^1^H NMR of **PPC8** |
| Supplementary Fig. 28 | ^1^H NMR of **PPC9** |
| Supplementary Fig. 29 | ^1^H NMR of **PPC10** |
| Supplementary Fig. 30 | ^1^H NMR of **PPC11** |
| Supplementary Fig. 31 | ^1^H NMR of **753b** |
| Supplementary Fig. 32 | ^13^C NMR of **753b** |
| Supplementary Fig. 33 | ^1^H NMR of **753a** |
| Supplementary Table 1 | The primers used in this study |
| Supplementary Methods | Chemical syntheses of PROTACs |

Supplementary Figures

**
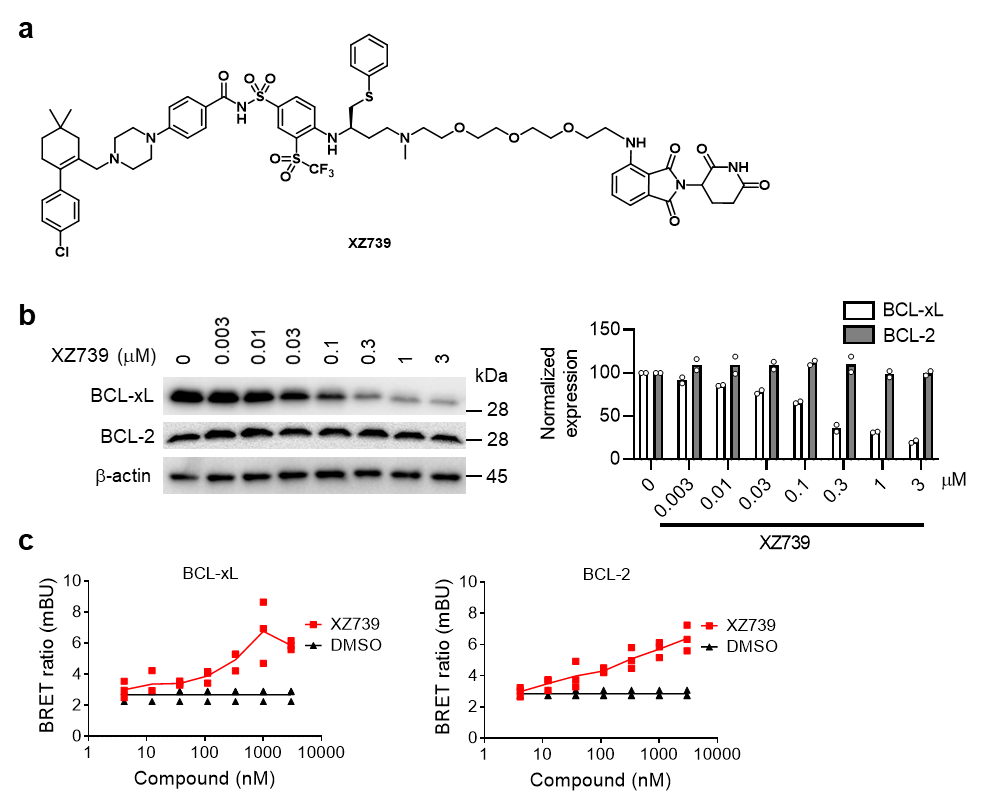
**

**Supplementary Fig. 1. CRBN-based PROTAC XZ739 can form a ternary complex with both BCL-xL and BCL-2 but only degrades BCL-xL. a**, The structure of XZ739. **b**, Representative immunoblots are shown to demonstrate that XZ739 degraded BCL-xL but not BCL-2 in 293T cells after the cells were treated with different concentrations of XZ739 for 16 h. β-actin was used as an equal loading control. The normalized protein content in the immunoblots is presented as mean values (*n* = 2 biologically independent experiments) in the bar graph (right panel). **c**, NanoBRET assays showed that XZ739 can form stable ternary complexes with both BCL-xL and BCL-2 in 293T cells. 293T cells were transiently transfected with HiBit-BCL-xL, LgBit and HaloTag-VHL or HiBit-BCL-2, LgBit and HaloTag-VHL and then treated with a serial dilution of the XZ739 for 6 h. Data are expressed as mean of three biological replicates. Source data are provided as a Source Data file.


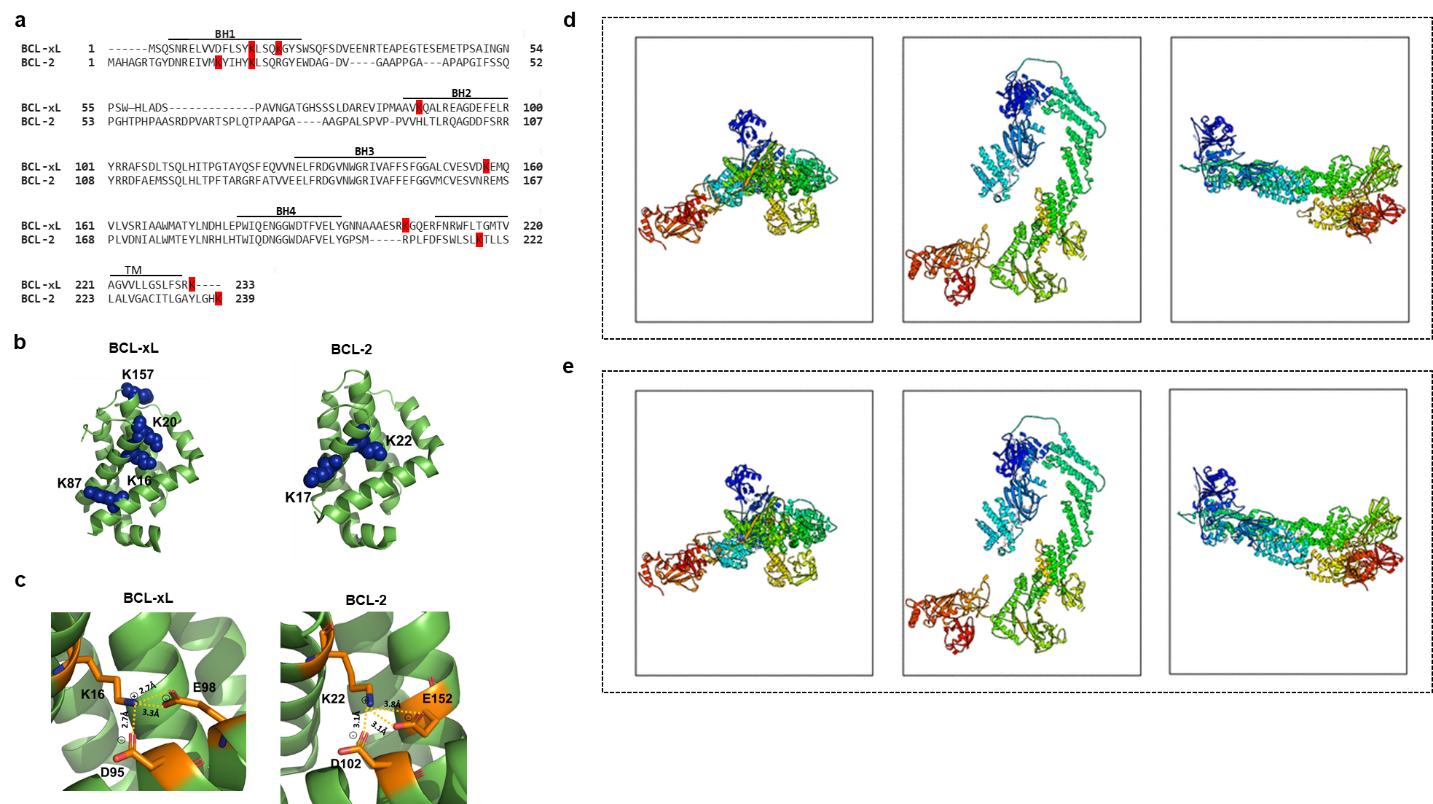


**Supplementary Fig. 2. Comparison of lysine distributions on BCL-xL and BCL-2 and the normal modes of the CRL^VHL^/DT2216/BCL-xL/E2-Ub/RBX1 complex. a**, The sequence alignment of BCL-xL and BCL-2 illustrates the localization of lysine residues on BCL-xL and BCL-2. Lysines are colored in red. **b**, The NMR structures of BCL-xL and BCL-2. **c**, Salt bridge and hydrogen bond formed by K16 of BCL-xL and K22 of BCL-2. **d**, Orthographic views of the major normal mode for BCL-xL approaching E2. **e**, Orthographic views of the major normal mode for BCL-xL wobbling around E2. The motion views of these two modes are presented in Supplementary Movies 1 and 2.

**Supplementary Fig. 3. Lysine to arginine mutations on BCL-xL do not affect the formation of the BCL-xL-DT2216-VHL ternary complex.** NanoBRET assays were used to compare the ternary complex formation of WT BCL-xL and K157-only and K-ko BCL-xL mutants with VHL and DT2216 in 293T cells. 293T cells were transiently transfected with HiBit-BCL-xL-WT /K157-only/K-ko, LgBit and HaloTag-VHL and then treated with a serial dilution of DT2216 for 6 h. WT represents wild type HiBit-BCL-xL; K157-only means that all the surface lysines on BCL-xL are mutated to arginines except K157; K-ko represents that all the surface lysines on BCL-xL are mutated to arginines. Data are expressed as mean ± s.e.m. of three biological replicates. Source data are provided as a Source Data file.


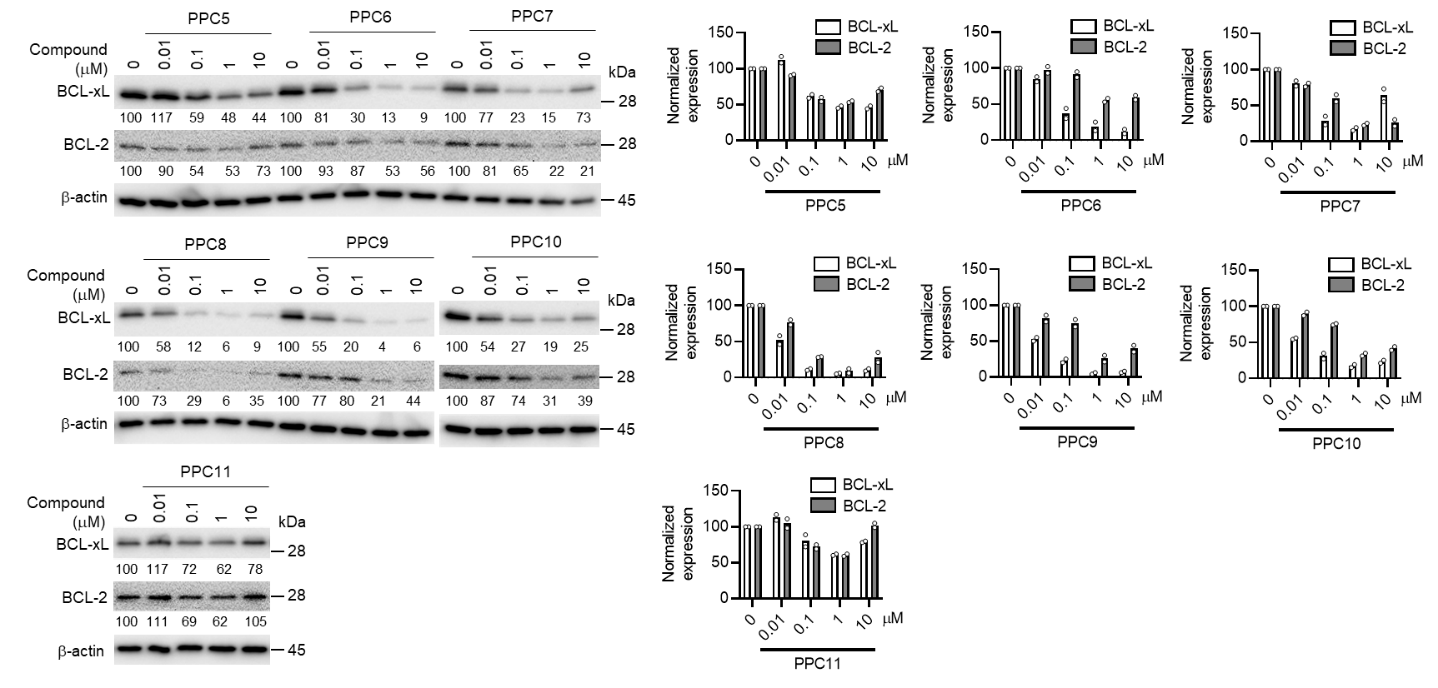


**Supplementary Fig. 4. BCL-xL/2 degradation induced by the new BCL-xL and/or BCL-2 PROTACs with different linker lengths.** 293T cells were treated with different concentrations of indicated PROTACs for 16 h. Representative immunoblots are shown and data are a representative of two independent experiments. β-actin was used as an equal loading control in all immunoblot analyses. The normalized protein content in the immunoblots is presented as mean values (*n* = 2 biologically independent experiments) in the bar graph (right panel). Source data are provided as a Source Data file.

**
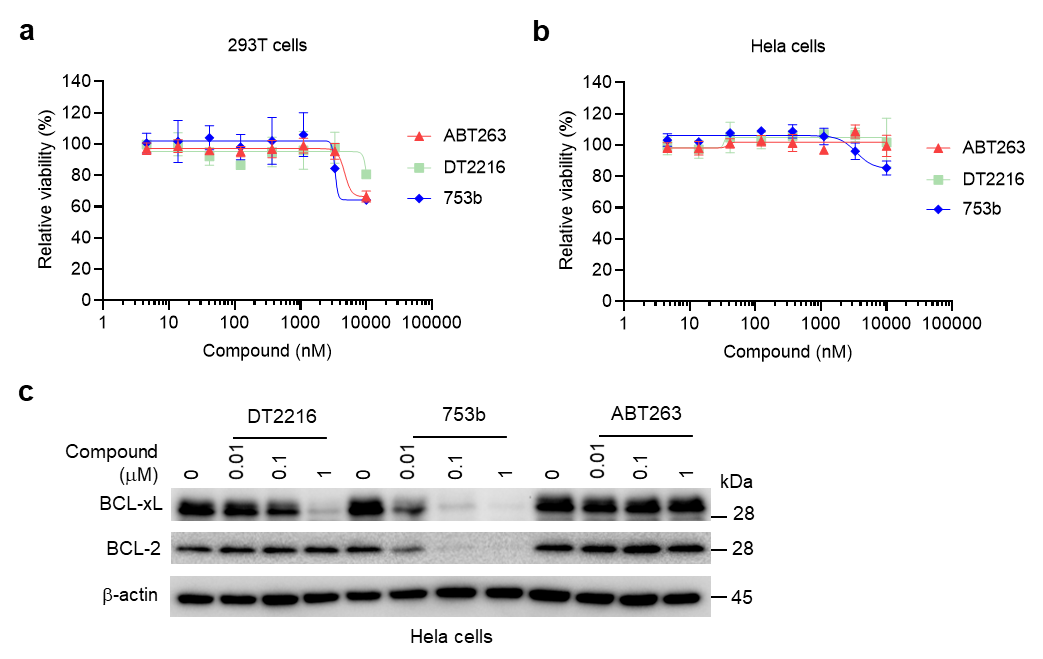
**

**Supplementary Fig. 5. 293T and Hela cells are not BCL-xL/2 dependent. a** and **b**, Viability of 293T (**a**) and Hela (**b**) cells were determined after they were incubated with increasing concentrations of ABT263, DT2216, or 753b for 72 h. The data are presented as mean ± s.d. from three replicate cell cultures in a representative experiment. Similar results were observed in two additional independent experiments. **c**, Comparison of BCL-xL/2 degradation in Hela cells under DT2216, 753b, or ABT263 treatment. Hela cells were treated with different concentrations of DT2216, 753b, or ABT263 for 16 h. Representative immunoblots are shown. β-actin was used as an equal loading control. Source data are provided as a Source Data file.

**
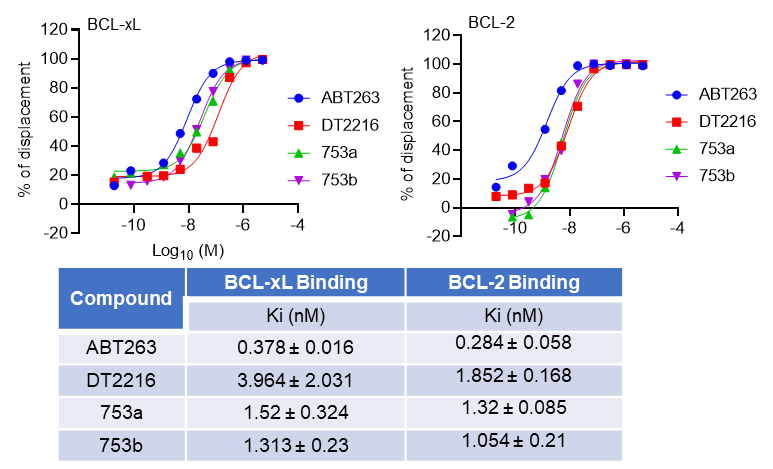
**

**Supplementary Fig. 6. BCL-xL and BCL-2 binary binding affinity determined by AlphaScreen assay.** The data from a representative AlphaScreen assay are presented in the top panels. Data represent the mean of a single experiment with 2 technical replicates. Similar results were obtained in one more independent experiment. Ki values (in nM) are presented in the table as the mean ± s.d. of two individual experiments performed in triplicates. Source data are provided as a Source Data file.

**
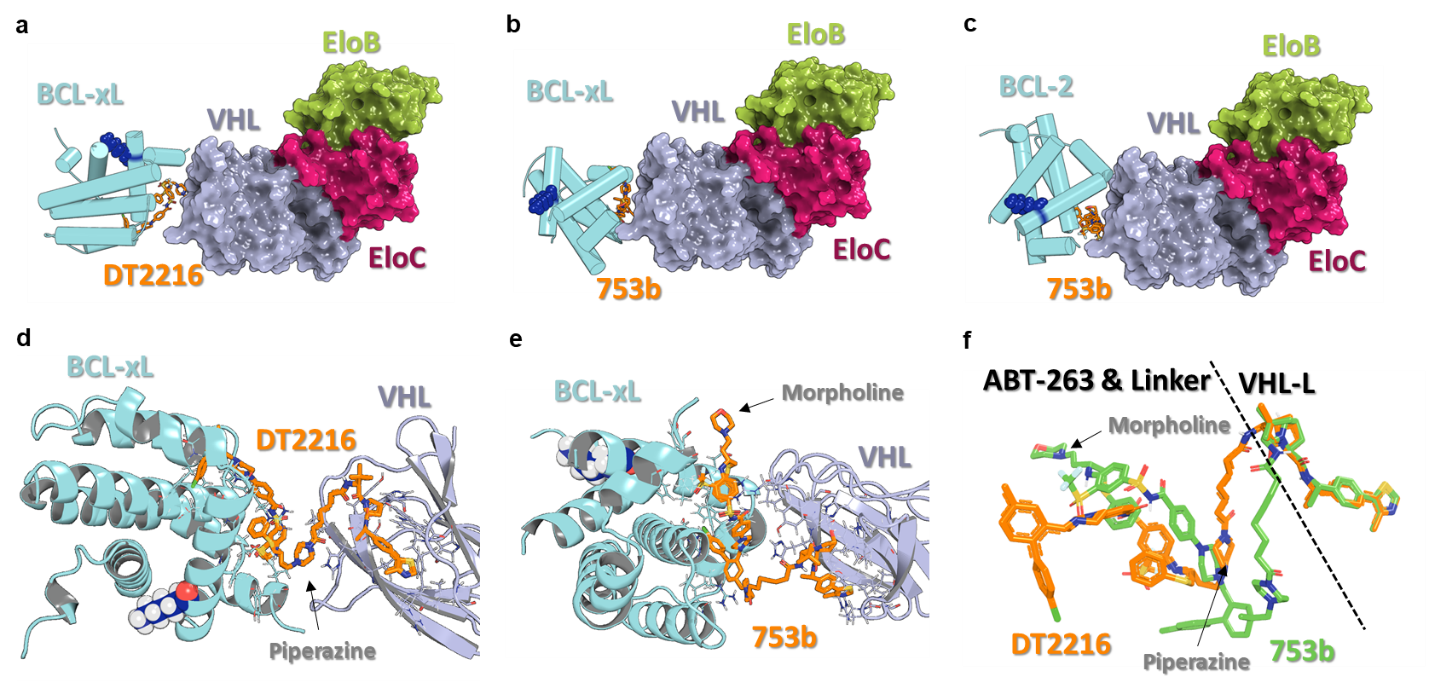
**

**Supplementary Fig. 7. The modelled ternary complexes. a**, The modelled ternary complex of VCB/DT2216/BCL-xL. K87 is shown in blue sphere. **b.** The modelled ternary complex of VCB/753b/BCL-xL. K87 is shown in blue sphere**. c.** The modelled ternary complex of VCB/753b/BCL-2. K17 is shown in blue sphere. **d.** The modelled ternary complex of VHL/DT2216/BCL-xL. Residues within 4Å around DT2216 are shown in lines style. K87 is shown in sphere style. **e.** The modelled ternary complex of VHL/753b/BCL-xL. K87 is shown in sphere style. **f.** The superimposed conformation of DT2216 and 753b in the modelled ternary complexes. DT2216 and 753b are colored in gold and green respectively.

**
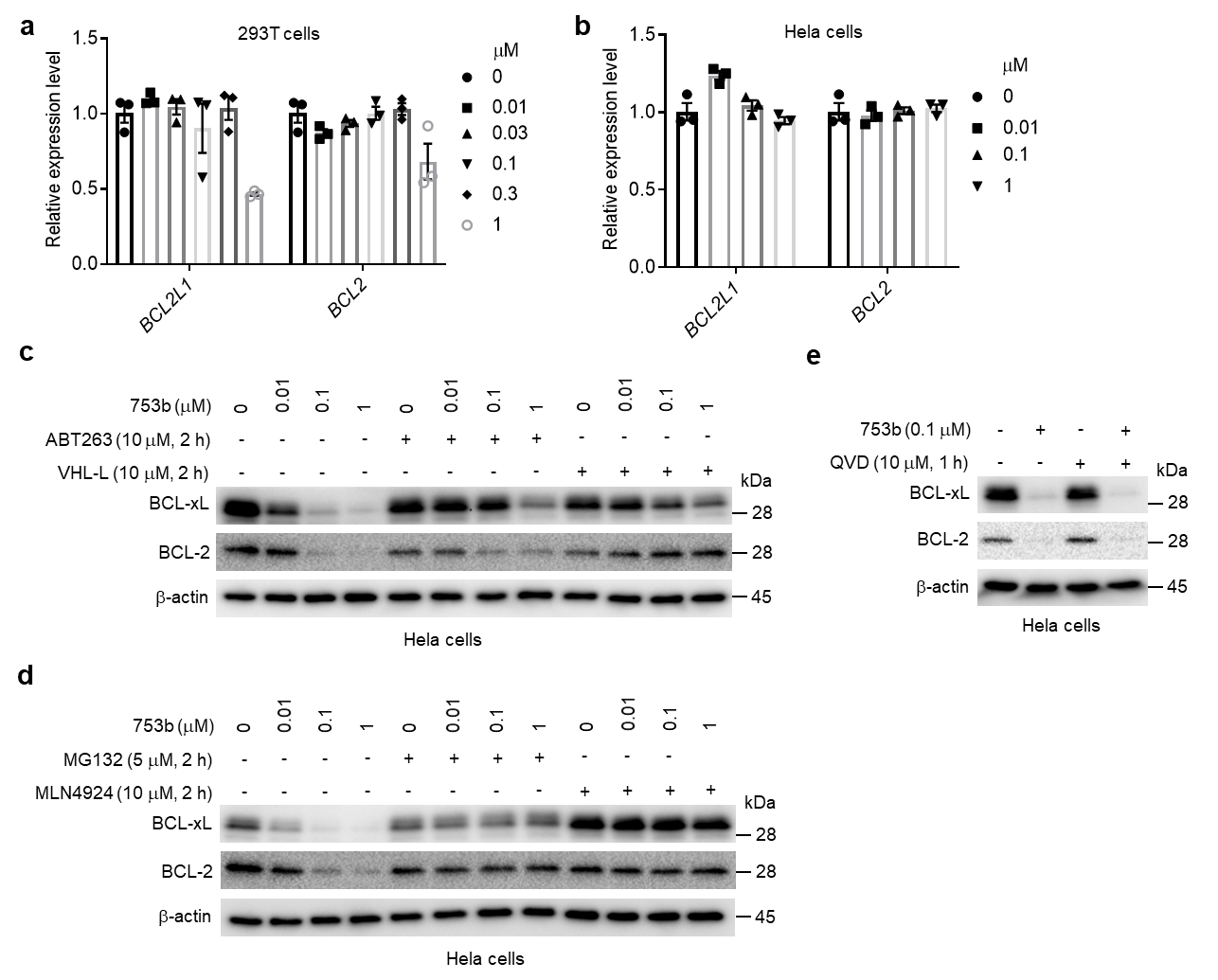
**

**Supplementary Fig. 8. Validation of the mechanism of 753b. a** and **b,** The levels of *BCL2L1* and *BCL2* gene expression were not changed under 753b treatment in 293T (**a**) or Hela cells (**b**). 293T or Hela cells were treated with different concentrations of 753b for 16 h and the expression of *BCL2L1* and *BCL2* mRNA were determined by qPCR. Data are expressed as mean ± s.e.m. of three biological replicates. **c**, Pretreatment with ABT263 or VHL ligand (VHL-L) blocks BCL-xL/2 degradation by 753b in Hela cells. Hela cells were treated with different concentrations 753b for 16 h with or without ABT263 or VHL-L pretreatment. **d**, BCL-xL/2 degradation induced by 753b in Hela cells is dependent on proteasome and activation E3 ligase by neddylation. Hela cells were treated with different concentrations of 753b for 16 h with or without the proteasome inhibitor MG132 or the neddylation inhibitor MLN4924 pretreatment. **e**, BCL-xL/2 degradation is not caspase-dependent in Hela cells. Hela cells were treated with different concentrations 753b for 16 h with or without the pan-caspase inhibitor QVD pretreatment. Representative immunoblots are shown in **c**-**e**. β-actin was used as an equal loading control in all immunoblot analyses. Source data are provided as a Source Data file.

**
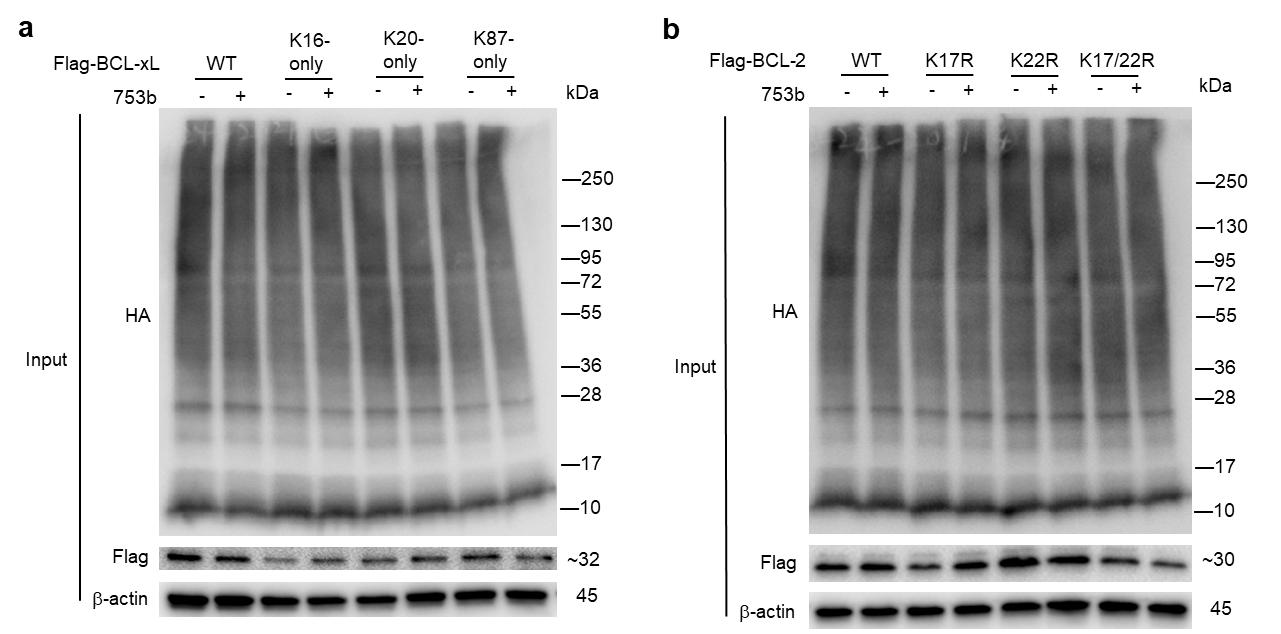
**

**Supplementary Fig. 9. Polyubiquitination of BCL-xL and BCL-2 induced by 753b.** **a**, The input (cell lysate before performing IP) results corresponding to **Fig. 5c** for analysis of 753b-induced BCL-xL polyubiquitination in 293T cells. **b**, The input results corresponding to **Fig. 5d** for analysis of 753b-induced BCL-2 polyubiquitination in 293T cells. 293T cells were co-transfected as indicated with Flag-BCL-xL WT or mutants and HA-tagged ubiquitin (HA-Ub) plasmids (**a**) or Flag-BCL-2-WT or mutants and HA-Ub plasmids (**b**). After 36 h, cells were pretreated with MG132 (10 µM) for 2 h and then treated with or without 753b (0.1 µM for BCL-xL and 1 µM for BCL-2) for 5 h. Data are representative of two independent experiments. Source data are provided as a Source Data file.

**
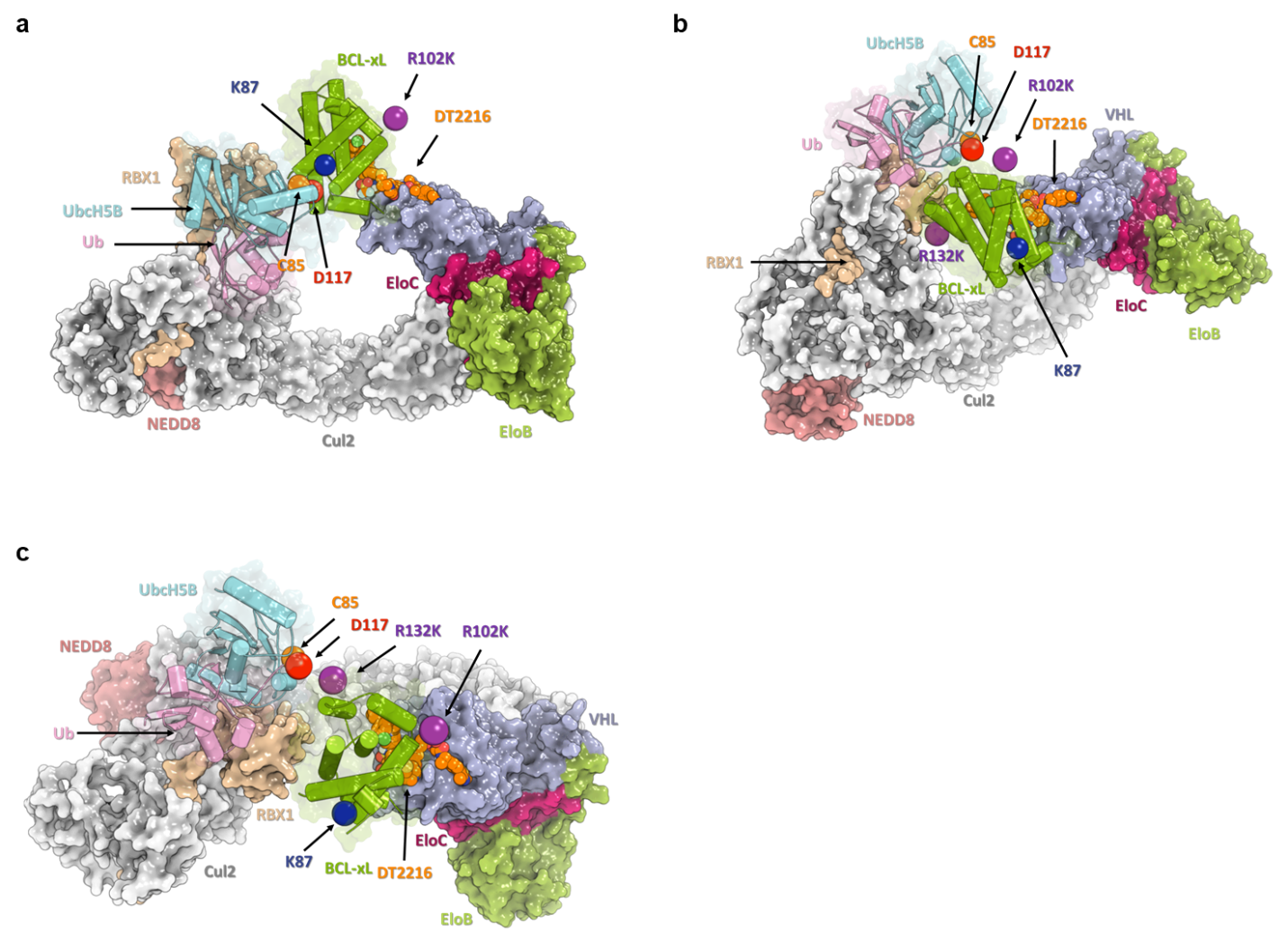
**

**Supplementary Fig. 10. Computational models of the CRL^VHL^/DT2216/BCL-xL/E2-Ub/RBX1 for UbcH5B-K87, UbcH5B-R102K and UbcH5B-R132K contacting status. a**, The computational model of CRL^VHL^/DT2216/BCL-xL/UbcH5B-Ub/RBX1 with UbcH5B contacting with K87 of BCL-xL. **b**, The computational model of CRL^VHL^/DT2216/BCL-xL/UbcH5B-Ub/RBX1 with UbcH5B contacting with R102K of BCL-xL. **c**, The computational model of CRL^VHL^/DT2216/BCL-xL/UbcH5B-Ub/RBX1 with UbcH5B contacting with R132K of BCL-xL. The K87 of BCL-xL is colored in blue; R102K and R132K of BCL-xL are colored in purple; and C85 and D117 of UbcH5B are colored in orange, and red, respectively.


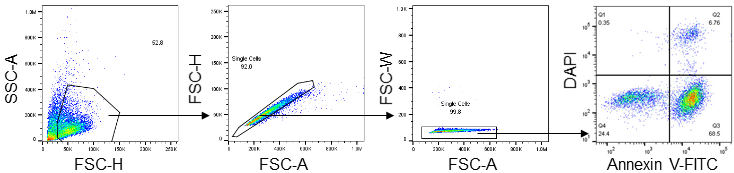
Supplementary Fig. 11. Gating strategy to determine the percentage of apoptotic cells in Kasium-1 cells by flow cytometry for Fig. 7.


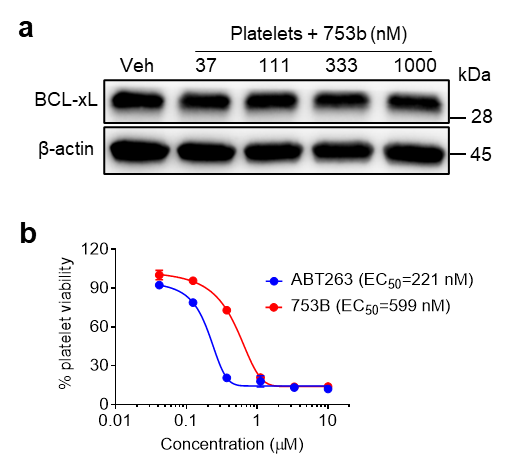


Supplementary Fig. 12. Evaluation of 753b in human platelets. a, A representative western blot image demonstrates that 753b cannot degrade BCL-xL in human platelets. b, Viability of human platelets was measured after they were incubated with increasing concentrations of ABT263 or 753b for 48 h. The data are presented as mean ± s.d. from three replicate platelet cultures in a representative experiment. Similar results were also observed in one additional independent experiment. Source data are provided as a Source Data file.

**Supplementary Fig. 13**. **The synthetic scheme of the PROTACs**

Supplementary Table

Supplementary Table 1. The primers used in this study

Supplementary Methods

Chemical syntheses of PROTACs. The synthetic scheme of the PROTACs presented in the manuscript is illustrated in Supplementary Fig. 13.

General Remarks. Chemicals obtained from commercial suppliers were used without further purification. Water was purified with Elga CLXXUVFM2 Purelab classic UVF water purification system. All reactions with water- and/or air-sensitive starting materials were carried out in pre-dried glass wares under argon atmosphere with standard procedure. THF, DCM, and DMF were obtained via a solvent purification system by filtering through two columns packed with activated alumina and 4 Å molecular sieve, respectively. Flash chromatography was performed using silica gel (230–400 mesh) as the stationary phase. Reaction progress was monitored by thin layer chromatography (silica coated glass plates) and visualized by UV light, and/or by LC-MS. ^1^H NMR spectra were recorded in CDCl_3_ at 600 MHz. Chemical shifts *δ* are given in ppm using tetramethylsilane as an internal standard. Multiplicities of NMR signals are designated as singlet (s), broad singlet (br s), doublet (d), doublet of doublets (dd), triplet (t), quartet (q), and multiplet (m). All final compounds for biological testing were of ≥ 95.0% purity as analyzed by LC–MS, performed on an Advion AVANT LC system with the expression CMS using a Thermo Accucore™ Vanquish™ C18+ UHPLC Column (1.5 µm, 50 x 2.1 mm) at 40 °C. Gradient elution was used for UHPLC with a mobile phase of acetonitrile and water containing 0.1% formic acid.

Synthesis of **PPC5-PPC11** commenced from racemic alcohol **1**^1^. Alcohol **1** was converted to the corresponding aldehyde **2** through Swern oxidation. Reductive amination of **2** with *tert*-butyl piperazine-1-carboxylate in the presence of NaBH(OAc)_3_ and TEA afforded compound **3**. Saponification of ester **3** under the treatment of LiOH gave acid **4**, which was then coupled with sulfonamide **5**^2^ to afford compound **6**. Boc-deprotection of **6** was achieved with 4N HCl in dioxane and the resultant amine **7** was coupled with acids **8.1-8.7**^3^ to afford **PPC5-PPC11**. **753b** and **753a** were synthesized by following the same synthetic protocol for **PPC5-PPC11**, with enantiopure alcohols **1b**/**1a**^1^ as the starting material.

**Synthetic Procedures.**

**Ethyl 4-(4-((4'-chloro-4-formyl-4-methyl-3,4,5,6-tetrahydro-[1,1'-biphenyl]-2-yl)methyl)piperazin-1-yl)benzoate** (**2**), **ethyl (*R*)-4-(4-((4'-chloro-4-formyl-4-methyl-3,4,5,6-tetrahydro-[1,1'-biphenyl]-2-yl)methyl)piperazin-1-yl)benzoate** (**2b**), **and** **ethyl (*S*)-4-(4-((4'-chloro-4-formyl-4-methyl-3,4,5,6-tetrahydro-[1,1'-biphenyl]-2-yl)methyl)piperazin-1-yl)benzoate** (**2a**): To a stirring solution of (COCl)_2_ (0.13 mL, 1.55 mmol) in DCM (6 mL) was added DMSO (0.22 mL, 3.09 mmol) dropwise at –78 °C and the mixture was kept stirring at the same temperature for 30 min. A solution of alcohol **1**, **1b**, or **1a** (500 mg, 1.03 mmol) dissolved in 10% DMSO solution in DCM (5 mL) was added to the reaction mixture dropwise at -78 °C and the mixture was continued stirring for 45 min. TEA (0.86 mL, 6.18 mmol) was added dropwise and the temperature was allowed to gradually rise to room temperature. The reaction mixture was diluted with DCM (100 mL) then washed with sat. NaHCO_3_ solution (15 mL), followed by water (15 mL) and brine (15 mL). The organic portion was dried over anhydrous Na_2_SO_4_, filtered, and then concentrated under reduced pressure to dryness. The crude product was purified by flash silica gel chromatography (30% EtOAc in hexanes) to afford the corresponding aldehyde **2** (464 mg), **2b** (464 mg), and **2a** (469 mg) as white powders in 94%, 94%, and 95% yield, respectively. MS and NMR data of **2**, **2b** and **2a** are identical. ^1^H NMR (600 MHz, CDCl_3_) δ 9.53 (s, 1H), 7.90 (d, *J* = 9.0 Hz, 2H), 7.27 (d, *J* = 8.4 Hz, 2H), 6.96 (d, *J* = 8.4 Hz, 2H), 6.82 (d, *J* = 9.1 Hz, 2H), 4.32 (q, *J* = 7.1 Hz, 2H), 3.27 (t, *J* = 5.1 Hz, 4H), 2.84 (q, *J* = 12.6 Hz, 2H), 2.66 (d, *J* = 17.6 Hz, 1H), 2.46 – 2.39 (m, 2H), 2.35 (q, *J* = 5.9, 5.4 Hz, 2H), 2.29 (d, *J* = 6.1 Hz, 2H), 1.99 (dt, *J* = 11.9, 5.5 Hz, 1H), 1.64 – 1.60 (m, 1H), 1.36 (t, *J* = 7.1 Hz, 3H), 1.15 (s, 3H).

^13^C NMR (150 MHz, Chloroform-d) δ 205.4, 166.8, 154.2, 141.1, 135.3, 132.5, 131.2, 129.7, 128.7, 128.5, 120.1, 113.7, 60.5, 60.4, 52.6, 47.7, 45.2, 33.9, 30.1, 29.2, 21.2, 14.6.

HRMS (ESI) m/z [M + H]^+^ calcd. For C_28_H_34_ClN_2_O_3_ 481.2252, found 481.2233.

***tert*-Butyl 4-((4'-chloro-6-((4-(4-(ethoxycarbonyl)phenyl)piperazin-1-yl)methyl)-4-methyl-2,3,4,5-tetrahydro-[1,1'-biphenyl]-4-yl)methyl)piperazine-1-carboxylate** (**3**), ***tert*-butyl (*R*)-4-((4'-chloro-6-((4-(4-(ethoxycarbonyl)phenyl)piperazin-1-yl)methyl)-4-methyl-2,3,4,5-tetrahydro-[1,1'-biphenyl]-4-yl)methyl)piperazine-1-carboxylate** (**3b**), **and *tert*-butyl (*S*)-4-((4'-chloro-6-((4-(4-(ethoxycarbonyl)phenyl)piperazin-1-yl)methyl)-4-methyl-2,3,4,5-tetrahydro-[1,1'-biphenyl]-4-yl)methyl)piperazine-1-carboxylate** (**3a**)**:** To a stirring solution of aldehyde **2**, **2b**, or **2a** (192 mg, 0.4 mmol) in DCM (6 mL) was successively added *tert*-butyl piperazine-1-carboxylate (111 mg, 0.6 mmol), NaBH(OAc)_3_ (508 mg, 2.4 mmol), TEA (0.67 mL, 4.8 mmol) at rt and the mixture was stirred for 5 h. After consumption of the starting material, the reaction mixture was diluted with DCM (100 mL) and washed with sat. NH_4_Cl solution (10 mL), followed by water (15 mL) and brine (15 mL). The organic portion was dried over anhydrous Na_2_SO_4_, filtered, and concentrated under reduced pressure to dryness. The crude product was purified by flash silica gel chromatography (45% EtOAc in hexanes) to afford **3** (236 mg), **3b** (231 mg), and **3a** (236 mg) as white powders in 89%, 91%, and 91% yield, respectively. MS and NMR data of **3**, **3b** and **3a** are identical. ^1^H NMR (600 MHz, CDCl_3_) δ 7.89 (d, *J* = 9.0 Hz, 2H), 7.27 (d, *J* = 8.4 Hz, 2H), 6.99 (d, *J* = 8.4 Hz, 2H), 6.81 (d, *J* = 9.1 Hz, 2H), 4.32 (q, *J* = 7.1 Hz, 2H), 3.40 (t, *J* = 5.0 Hz, 4H), 3.24 (t, *J* = 5.1 Hz, 4H), 2.79 (s, 2H), 2.49 (d, *J* = 5.2 Hz, 4H), 2.35 (qt, *J* = 11.0, 4.8 Hz, 4H), 2.22 (d, *J* = 3.8 Hz, 3H), 2.13 (d, *J* = 17.5 Hz, 1H), 1.93 (d, *J* = 18.5 Hz, 1H), 1.63 – 1.56 (m, 3H), 1.45 (s, 9H), 1.36 (t, *J* = 7.1 Hz, 3H), 0.95 (s, 3H).

^13^C NMR (150 MHz, Chloroform-d) δ 166.8, 155.0, 154.3, 141.9, 134.8, 132.2, 131.3, 129.9, 129.5, 128.4, 120.0, 113.6, 79.6, 77.4, 68.5, 60.7, 60.4, 55.9, 52.6, 47.7, 37.9, 34.6, 31.9, 30.5, 28.6, 23.4, 14.6.

HRMS (ESI) m/z [M + H]^+^ calcd. For C_37_H_52_ClN_4_O_4_ 651.3672, found. 651.3648.

**4-(4-((4-((4-(*tert*-butoxycarbonyl)piperazin-1-yl)methyl)-4'-chloro-4-methyl-3,4,5,6-tetrahydro-[1,1'-biphenyl]-2-yl)methyl)piperazin-1-yl)benzoic acid** (**4**), **(*R*)-4-(4-((4-((4-(tert-butoxycarbonyl)piperazin-1-yl)methyl)-4'-chloro-4-methyl-3,4,5,6-tetrahydro-[1,1'-biphenyl]-2-yl)methyl)piperazin-1-yl)benzoic acid** (**4b**), **and (*S*)-4-(4-((4-((4-(*tert*-butoxycarbonyl)piperazin-1-yl)methyl)-4'-chloro-4-methyl-3,4,5,6-tetrahydro-[1,1'-biphenyl]-2-yl)methyl)piperazin-1-yl)benzoic acid** (**4a**)**:** To a stirring solution of **3**, **3b**, or **3a** (210 mg, 0.32 mmol) in THF (4 mL) was added MeOH (2 mL) and LiOH·H_2_O (40 mg, 0.96 mmol) dissolved in water (2 mL). After stirring for 12 h at rt, the reaction was quenched with (1N) HCl solution (1 mL). The volatiles were removed under reduced pressure and EtOAc (70 mL) was added to it. The organic portion was washed with water (10 mL) and brine (10 mL), dried over anhydrous Na_2_SO_4_, filtered, and concentrated under reduced pressure to dryness. The crude product was purified by flash silica gel chromatography (60% EtOAc in hexanes) to afford the corresponding acid **4** (181 mg), **4b** (179 mg), and **4a** (181 mg) as white power in 90%, 91%, and 91% yield, respectively. MS and spectral data of **4**, **4b** and **4a** are identical. ^1^H NMR (600 MHz, CDCl_3_) δ 7.93 (d, *J* = 8.8 Hz, 2H), 7.27 (d, *J* = 8.4 Hz, 2H), 6.99 (d, *J* = 8.4 Hz, 2H), 6.84 – 6.79 (m, 2H), 3.39 (s, 4H), 3.28 (t, *J* = 5.2 Hz, 4H), 2.81 (d, *J* = 4.5 Hz, 2H), 2.49 (s, 4H), 2.36 (dd, *J* = 7.2, 4.3 Hz, 4H), 2.28 (d, *J* = 8.5 Hz, 1H), 2.22 (d, *J* = 3.9 Hz, 3H), 2.14 (d, *J* = 17.5 Hz, 1H), 1.93 (d, *J* = 17.3 Hz, 1H), 1.63 – 1.58 (m, 1H), 1.45 (s, 9H), 0.95 (s, 3H).

^13^C NMR (150 MHz, Chloroform-d) δ 171.4, 155.1, 154.5, 141.9, 135.5, 132.3, 131.9, 129.9, 128.9, 128.5, 119.6, 113.6, 79.7, 68.7, 60.6, 55.9, 53.6, 52.5, 47.3, 37.9, 34.6, 31.9, 30.6, 28.6, 23.2.

HRMS (ESI) m/z [M + H]^+^ calcd. For C_35_H_48_ClN_4_O_4_ 623.3359, found. 623.3330.

***tert*-Butyl 4-((4'-chloro-4-methyl-6-((4-(4-(((4-(((*R*)-4-morpholino-1-(phenylthio)butan-2-yl)amino)-3-((trifluoromethyl)sulfonyl)phenyl)sulfonyl)carbamoyl)phenyl)piperazin-1-yl)methyl)-2,3,4,5-tetrahydro-[1,1'-biphenyl]-4-yl)methyl)piperazine-1-carboxylate** (**6**), ***tert*-butyl 4-(((*R*)-4'-chloro-4-methyl-6-((4-(4-(((4-(((*R*)-4-morpholino-1-(phenylthio)butan-2-yl)amino)-3-((trifluoromethyl)sulfonyl)phenyl)sulfonyl)carbamoyl)phenyl)piperazin-1-yl)methyl)-2,3,4,5-tetrahydro-[1,1'-biphenyl]-4-yl)methyl)piperazine-1-carboxylate** (**6b**), **and** ***tert*-butyl 4-(((*S*)-4'-chloro-4-methyl-6-((4-(4-(((4-(((*R*)-4-morpholino-1-(phenylthio)butan-2-yl)amino)-3-((trifluoromethyl)sulfonyl)phenyl)sulfonyl)carbamoyl)phenyl)piperazin-1-yl)methyl)-2,3,4,5-tetrahydro-[1,1'-biphenyl]-4-yl)methyl)piperazine-1-carboxylate** (**6a**): To a stirring solution of the compound **4**, **4b**, or **4a** (100 mg, 0.16 mmol) in DCM (5 mL) was added compound **5** (80 mg, 0.14 mmol) followed by addition of EDCI·HCl (91 mg, 0.48 mmol), DMAP (19 mg, 0.16 mmol) and TEA (0.04 mL, 0.32 mmol), and the reaction was stirred for 10 h at room temperature. Upon completion of the reaction, the mixture was diluted with DCM (60 mL). The mixture was washed with sat. NH_4_Cl solution (10 mL) followed by water (15 mL) and brine (15 mL). The organic portion was dried over anhydrous Na_2_SO_4_, filtered, and concentrated under reduced pressure to dryness. The crude product was purified by flash silica gel chromatography (0% to 10% MeOH in DCM) to afford the compound **6** (137 mg), **6b** (137 mg), and **6a** (136 mg) as white powders in 85%, 84%, and 85% yield, respectively.

Compound **6**: ^1^H NMR (600 MHz, CDCl_3_) δ 8.36 (d, *J* = 2.3 Hz, 1H), 8.11 (dd, *J* = 9.2, 2.3 Hz, 1H), 7.63 (d, *J* = 8.8 Hz, 2H), 7.37 (d, *J* = 7.2 Hz, 2H), 7.31 (t, *J* = 7.4 Hz, 2H), 7.28 (d, *J* = 8.4 Hz, 2H), 7.07 (d, *J* = 8.6 Hz, 1H), 6.98 (d, *J* = 8.4 Hz, 2H), 6.78 (d, *J* = 9.2 Hz, 2H), 6.61 (d, *J* = 9.4 Hz, 1H), 3.94 – 3.86 (m, 1H), 3.66 (p, *J* = 7.2, 6.1 Hz, 4H), 3.40 (s, 4H), 3.27 (s, 4H), 3.10 (dd, *J* = 13.9, 5.1 Hz, 1H), 3.02 (dd, *J* = 13.9, 7.3 Hz, 1H), 2.83 (s, 2H), 2.49 (s, 4H), 2.44 (s, 2H), 2.39-2.34 (m, 5H), 2.34 – 2.25 (m, 4H), 2.25-2.18 (s, 3H), 2.14 – 2.11 (m, 2H), 1.92 (d, *J* = 17.2 Hz, 1H), 1.67 (td, *J* = 14.1, 5.6 Hz, 1H), 1.60 (dt, *J* = 14.3, 7.6 Hz, 1H), 1.45 (s, 9H), 1.46-1.44 (m, 1H), 0.94 (s, 3H). LC-MS (ESI): m/z 1158.3 [M + H]^+^.

Compound **6b**: ^1^H NMR (600 MHz, CDCl_3_) δ 8.33 (s, 1H), 8.09 (dd, *J* = 9.2, 2.4 Hz, 1H), 7.67 (d, *J* = 8.5 Hz, 2H), 7.36 (d, *J* = 7.6 Hz, 2H), 7.29 (t, *J* = 7.6 Hz, 2H), 7.27 (s, 1H), 7.26 (s, 1H), 7.03 (d, *J* = 8.8 Hz, 1H), 6.98 (d, *J* = 8.4 Hz, 2H), 6.76 (d, *J* = 8.6 Hz, 2H), 6.59 (d, *J* = 9.3 Hz, 1H), 3.89 (s, 1H), 3.65 (q, *J* = 5.3 Hz, 5H), 3.39 (t, *J* = 5.1 Hz, 4H), 3.25 (d, *J* = 5.4 Hz, 4H), 3.09 (dd, *J* = 13.9, 5.0 Hz, 1H), 3.01 (dd, *J* = 13.9, 7.2 Hz, 1H), 2.83 (s, 2H), 2.48 (s, 4H), 2.43 (s, 2H), 2.36 (d, *J* = 5.5 Hz, 8H), 2.20 (d, *J* = 3.2 Hz, 3H), 2.13 (d, *J* = 17.0 Hz, 2H), 1.92 (d, *J* = 18.4 Hz, 1H), 1.69 – 1.65 (m, 1H), 1.61 – 1.56 (m, 1H), 1.44 (s, 9H), 0.93 (s, 3H).

^13^C NMR (150 MHz, Chloroform-d) δ 166.7, 155.1, 154.0, 151.7, 141.6, 137.9, 136.3, 134.8, 132.5, 131.3, 130.1, 129.8, 129.5, 128.6, 128.4, 128.2, 127.6, 122.2, 121.4, 119.2, 113.8, 113.1, 108.7, 79.7, 68.5, 66.8, 60.6, 55.9, 54.7, 53.7, 52.5, 50.8, 47.1, 39.2, 37.7, 34.6, 31.8, 30.6, 30.1, 28.6, 23.2.

HRMS (ESI) m/z [M + H]^+^ calcd. For C_56_H_72_ClF_3_N_7_O_8_S_3_ 1158.4240, found. 1158.4276.

Compound **6a**: ^1^H NMR (600 MHz, CDCl_3_) δ 8.34 (s, 1H), 8.09 (d, *J* = 10.2 Hz, 1H), 7.66 (d, *J* = 8.3 Hz, 2H), 7.36 (d, *J* = 7.4 Hz, 2H), 7.28 (d, *J* = 12.8 Hz, 4H), 7.04 (s, 1H), 6.98 (d, *J* = 8.4 Hz, 2H), 6.75 (d, *J* = 8.6 Hz, 2H), 6.59 (d, *J* = 9.3 Hz, 1H), 3.89 (s, 1H), 3.65 (s, 5H), 3.38 (s, 4H), 3.25 (s, 4H), 3.09 (dd, *J* = 13.8, 4.8 Hz, 1H), 3.01 (dd, *J* = 14.0, 7.2 Hz, 1H), 2.82 (s, 2H), 2.48 (s, 3H), 2.35 (s, 5H), 2.32 (s, 3H), 2.21 (d, *J* = 3.9 Hz, 3H), 2.17 (s, 3H), 2.11 (s, 1H), 1.92 (d, *J* = 17.3 Hz, 2H), 1.67 (q, *J* = 7.6 Hz, 1H), 1.62 – 1.55 (m, 1H), 1.45 (s, 9H), 0.94 (s, 3H). LC-MS (ESI): m/z 1,158.4 [M + H]^+^.

**4-(4-((4'-Chloro-4-methyl-4-(piperazin-1-ylmethyl)-3,4,5,6-tetrahydro-[1,1'-biphenyl]-2-yl)methyl)piperazin-1-yl)-N-((4-(((*R*)-4-morpholino-1-(phenylthio)butan-2-yl)amino)-3-((trifluoromethyl)sulfonyl)phenyl)sulfonyl)benzamide hydrochloride** (**7**), **4-(4-(((*R*)-4'-chloro-4-methyl-4-(piperazin-1-ylmethyl)-3,4,5,6-tetrahydro-[1,1'-biphenyl]-2-yl)methyl)piperazin-1-yl)-N-((4-(((*R*)-4-morpholino-1-(phenylthio)butan-2-yl)amino)-3-((trifluoromethyl)sulfonyl)phenyl)sulfonyl)benzamide hydrochloride** (**7b**), **and** **4-(4-(((*S*)-4'-chloro-4-methyl-4-(piperazin-1-ylmethyl)-3,4,5,6-tetrahydro-[1,1'-biphenyl]-2-yl)methyl)piperazin-1-yl)-N-((4-(((*R*)-4-morpholino-1-(phenylthio)butan-2-yl)amino)-3-((trifluoromethyl)sulfonyl)phenyl)sulfonyl)benzamide hydrochloride** (**7a**): To a stirring solution of the **6** (100 mg, 0.086 mmol), **6b** (100 mg, 0.086 mmol), or **6a** (100 mg, 0.086 mmol) in DCM (3 mL) was added 4N HCl in dioxane (0.2 mL, 0.86 mmol) and the reaction mixture stirred at rt for 5 h. After completion of the reaction the volatiles were removed and the remaining solid was washed with diethyl ether (5 mL). The off-white crude salt was used in the next step without further purification. Compounds **7**, **7b** and **7b** have identical MS. LC-MS (ESI): m/z 1,058.5 [M + H]^+^.

**General procedure for the synthesis of final PROTACs**

To a stirring suspension of the hydrochloride salt **7** (16 mg, 0.015 mmol) in DCM (2 mL) was added TEA (20 µL, 0.15 mmol) followed by the corresponding acid (0.015 mmol) and HATU (6.3 mg, 0.016 mmol). The mixture was stirred at rt for 5 h. After completion, the mixture was diluted with DCM (50 mL) and washed with sat. NH_4_Cl solution (10 mL) followed by water (10 mL) and brine (10 mL). The organic portion was dried over anhydrous Na_2_SO_4_, filtered, and concentrated under reduced pressure to dryness. The crude product was purified by flash silica gel chromatography (0% to 15% MeOH in DCM) to afford the final compounds, which were further purified by preparative TLC. The average yield is 60%.

**(2*S*,4*R*)-1-((2*S*)-2-(5-(4-((4'-Chloro-4-methyl-6-((4-(4-(((4-(((*R*)-4-morpholino-1-(phenylthio)butan-2-yl)amino)-3-((trifluoromethyl)sulfonyl)phenyl)sulfonyl)carbamoyl)phenyl)piperazin-1-yl)methyl)-2,3,4,5-tetrahydro-[1,1'-biphenyl]-4-yl)methyl)piperazin-1-yl)-5-oxopentanamido)-3,3-dimethylbutanoyl)-4-hydroxy-N-((*S*)-1-(4-(4-methylthiazol-5-yl)phenyl)ethyl)pyrrolidine-2-carboxamide** (**PPC5**): ^1^H NMR (600 MHz, CDCl_3_) δ 8.67 (s, 1H), 8.32 (s, 1H), 8.09 (d, *J* = 9.1 Hz, 1H), 7.70 (t, *J* = 9.2 Hz, 2H), 7.57 – 7.51 (m, 1H), 7.37 (q, *J* = 7.8, 7.3 Hz, 6H), 7.31 – 7.23 (m, 5H), 7.02 (d, *J* = 8.5 Hz, 1H), 6.98 (d, *J* = 8.3 Hz, 2H), 6.91 (d, J = 7.1 Hz, 1H), 6.74 (dd, *J* = 8.5, 4.0 Hz, 2H), 6.60 (d, *J* = 9.4 Hz, 1H), 5.13 – 5.06 (m, 1H), 4.73 (t, *J* = 7.7 Hz, 1H), 4.54 (dd, *J* = 8.2, 5.5 Hz, 1H), 4.47 (s, 1H), 4.11 (d, *J* = 7.5 Hz, 1H), 3.93 – 3.85 (m, 1H), 3.69 – 3.63 (m, 4H), 3.57 (dt, *J* = 21.2, 10.6 Hz, 3H), 3.40 (s, 2H), 3.26 (s, 4H), 3.10 (dd, *J* = 13.8, 5.0 Hz, 1H), 3.02 (dd, *J* = 13.8, 7.1 Hz, 1H), 2.92 (s, 2H), 2.53 (d, *J* = 17.2 Hz, 3H), 2.49 (d, *J* = 2.2 Hz, 3H), 2.44 (s, 6H), 2.40 – 2.16 (m, 15H), 2.15 – 2.03 (m, 2H), 1.98 – 1.83 (m, 3H), 1.72 – 1.56 (m, 2H), 1.46 (d, *J* = 6.8 Hz, 3H), 1.42 (d, J = 6.4 Hz, 1H), 1.28 (s, 1H), 1.05 (s, 9H), 0.93 (d, *J* = 2.7 Hz, 3H).

HRMS (ESI) m/z [M + H]^+^ calcd. For C_79_H_100_ClF_3_N_11_O_11_S_4_ 1598.6122, found. 1598.6193.

**(2*S*,4*R*)-1-((2*S*)-2-(6-(4-((4'-Chloro-4-methyl-6-((4-(4-(((4-(((*R*)-4-morpholino-1-(phenylthio)butan-2-yl)amino)-3-((trifluoromethyl)sulfonyl)phenyl)sulfonyl)carbamoyl)phenyl)piperazin-1-yl)methyl)-2,3,4,5-tetrahydro-[1,1'-biphenyl]-4-yl)methyl)piperazin-1-yl)-6-oxohexanamido)-3,3-dimethylbutanoyl)-4-hydroxy-N-((*S*)-1-(4-(4-methylthiazol-5-yl)phenyl)ethyl)pyrrolidine-2-carboxamide** (**PPC6**): ^1^H NMR (600 MHz, CDCl_3_) δ 8.67 (s, 1H), 8.33 – 8.30 (m, 1H), 8.10 (d, J = 8.9 Hz, 1H), 7.70 (t, *J* = 8.6 Hz, 2H), 7.47 (dd, *J* = 17.6, 7.8 Hz, 1H), 7.40 – 7.35 (m, 6H), 7.27 (td, *J* = 19.9, 17.7, 7.3 Hz, 5H), 7.03 (d, *J* = 8.1 Hz, 1H), 6.98 (d, *J* = 8.0 Hz, 2H), 6.74 (dd, *J* = 8.8, 4.2 Hz, 2H), 6.60 (d, *J* = 9.4 Hz, 1H), 6.56 (t, *J* = 9.5 Hz, 1H), 5.13 – 5.04 (m, 1H), 4.75 (q, *J* = 7.8 Hz, 1H), 4.63 (dd, *J* = 8.7, 4.5 Hz, 1H), 4.48 (s, 1H), 4.11 (d, *J* = 11.0 Hz, 1H), 3.90 (d, *J* = 6.6 Hz, 1H), 3.67 (t, *J* = 8.4 Hz, 4H), 3.61 – 3.49 (m, 3H), 3.39 (s, 2H), 3.25 (s, 4H), 3.10 (dd, *J* = 13.9, 4.9 Hz, 1H), 3.02 (dd, *J* = 13.8, 7.1 Hz, 1H), 2.89 (s, 2H), 2.50 (s, 9H), 2.47 – 2.29 (m, 11H), 2.29 – 2.04 (m, 12H), 1.91 (d, *J* = 16.8 Hz, 1H), 1.68 (dd, *J* = 14.1, 8.1 Hz, 1H), 1.59 (s, 3H), 1.46 (d, *J* = 6.9 Hz, 3H), 1.42 (dd, *J* = 11.9, 5.7 Hz, 1H), 1.28 (s, 1H), 1.05 (s, 9H), 0.94 (s, 3H).

HRMS (ESI) m/z [M + H]^+^ calcd. For C_80_H_102_ClF_3_N_11_O_11_S_4_ 1612.6278, found. 1612.6346.

**(2*S*,4*R*)-1-((2*S*)-2-(7-(4-((4'-Chloro-4-methyl-6-((4-(4-(((4-(((*R*)-4-morpholino-1-(phenylthio)butan-2-yl)amino)-3-((trifluoromethyl)sulfonyl)phenyl)sulfonyl)carbamoyl)phenyl)piperazin-1-yl)methyl)-2,3,4,5-tetrahydro-[1,1'-biphenyl]-4-yl)methyl)piperazin-1-yl)-7-oxoheptanamido)-3,3-dimethylbutanoyl)-4-hydroxy-N-((*S*)-1-(4-(4-methylthiazol-5-yl)phenyl)ethyl)pyrrolidine-2-carboxamide** (**PPC7**): ^1^H NMR (600 MHz, CDCl_3_) δ 8.67 (s, 1H), 8.32 (s, 1H), 8.10 (d, *J* = 9.1 Hz, 1H), 7.69 (dd, *J* = 12.3, 9.0 Hz, 2H), 7.45 – 7.34 (m, 7H), 7.29 (dd, *J* = 17.6, 7.9 Hz, 5H), 7.04 (d, *J* = 8.2 Hz, 1H), 6.98 (d, *J* = 8.3 Hz, 2H), 6.74 (dd, *J* = 8.7, 4.8 Hz, 2H), 6.61 (d, *J* = 9.4 Hz, 1H), 6.35 (dd, *J* = 19.3, 8.7 Hz, 1H), 5.09 (dt, *J* = 13.6, 6.8 Hz, 1H), 4.73 (dt, *J* = 10.8, 8.0 Hz, 1H), 4.63 (t, *J* = 8.6 Hz, 1H), 4.48 (s, 1H), 4.10 (d, *J* = 11.2 Hz, 1H), 3.94 – 3.86 (m, 1H), 3.66 (s, 4H), 3.58 (d, *J* = 10.3 Hz, 3H), 3.40 (s, 2H), 3.25 (s, 4H), 3.10 (dd, *J* = 13.8, 4.9 Hz, 1H), 3.02 (dd, *J* = 13.8, 7.2 Hz, 1H), 2.93 – 2.80 (m, 2H), 2.63 – 2.41 (m, 11H), 2.41 – 2.30 (m, 6H), 2.30 – 2.03 (m, 11H), 1.91 (d, *J* = 20.2 Hz, 1H), 1.59 (ddd, *J* = 30.7, 14.5, 7.2 Hz, 7H), 1.46 (dd, *J* = 6.8, 2.3 Hz, 3H), 1.45 – 1.38 (m, 1H), 1.35 – 1.27 (m, 3H), 1.04 (s, 9H), 0.96 – 0.92 (m, 3H).

HRMS (ESI) m/z [M + H]^+^ calcd. For C_81_H_104_ClF_3_N_11_O_11_S_4_ 1626.6435, found. 1626.6513.

**(2*S*,4*R*)-1-((2*S*)-2-(8-(4-((4'-Chloro-4-methyl-6-((4-(4-(((4-(((*R*)-4-morpholino-1-(phenylthio)butan-2-yl)amino)-3-((trifluoromethyl)sulfonyl)phenyl)sulfonyl)carbamoyl)phenyl)piperazin-1-yl)methyl)-2,3,4,5-tetrahydro-[1,1'-biphenyl]-4-yl)methyl)piperazin-1-yl)-8-oxooctanamido)-3,3-dimethylbutanoyl)-4-hydroxy-N-((*S*)-1-(4-(4-methylthiazol-5-yl)phenyl)ethyl)pyrrolidine-2-carboxamide** (**PPC8**): ^1^H NMR (600 MHz, CDCl_3_) δ 8.67 (s, 1H), 8.32 (s, 1H), 8.10 (d, *J* = 9.1 Hz, 1H), 7.71 (t, *J* = 7.5 Hz, 2H), 7.44 (dd, *J* = 27.6, 7.8 Hz, 1H), 7.37 (dd, *J* = 15.4, 6.6 Hz, 6H), 7.31 – 7.23 (m, 5H), 7.03 (d, *J* = 8.4 Hz, 1H), 6.98 (d, *J* = 8.2 Hz, 2H), 6.74 (d, *J* = 7.4 Hz, 2H), 6.60 (d, *J* = 9.4 Hz, 1H), 6.33 (dd, *J* = 13.9, 8.9 Hz, 1H), 5.12 – 5.05 (m, 1H), 4.72 (q, *J* = 7.9 Hz, 1H), 4.64 – 4.59 (m, 1H), 4.49 (s, 1H), 4.10 (d, *J* = 11.3 Hz, 1H), 3.89 (s, 1H), 3.66 (s, 4H), 3.58 (d, *J* = 11.4 Hz, 3H), 3.41 (s, 2H), 3.24 (s, 4H), 3.09 (dd, *J* = 13.8, 4.8 Hz, 1H), 3.02 (dd, *J* = 13.8, 7.1 Hz, 1H), 2.87 (s, 2H), 2.50 (s, 8H), 2.38 (ddt, *J* = 24.5, 18.9, 9.7 Hz, 10H), 2.23 (dq, *J* = 25.6, 7.6 Hz, 8H), 2.14 – 2.05 (m, 2H), 1.91 (dd, J = 16.5, 6.8 Hz, 1H), 1.66 (s, 1H), 1.58 – 1.56 (m, 2H), 1.45 (d, *J* = 6.6 Hz, 4H), 1.26 (d, *J* = 14.6 Hz, 8H), 1.04 (s, 9H), 0.94 (s, 3H). LC-MS (ESI): m/z 1641.1 [M + H]^+^.

**(2*S*,4*R*)-1-((2*S*)-2-(9-(4-((4'-Chloro-4-methyl-6-((4-(4-(((4-(((*R*)-4-morpholino-1-(phenylthio)butan-2-yl)amino)-3-((trifluoromethyl)sulfonyl)phenyl)sulfonyl)carbamoyl)phenyl)piperazin-1-yl)methyl)-2,3,4,5-tetrahydro-[1,1'-biphenyl]-4-yl)methyl)piperazin-1-yl)-9-oxononanamido)-3,3-dimethylbutanoyl)-4-hydroxy-N-((*S*)-1-(4-(4-methylthiazol-5-yl)phenyl)ethyl)pyrrolidine-2-carboxamide** (**PPC9**): ^1^H NMR (600 MHz CDCl_3_) δ 8.67 (s, 1H), 8.32 (d, *J* = 2.3 Hz, 1H), 8.11 (dd, *J* = 9.3, 2.3 Hz, 1H), 7.69 (dd, *J* = 8.9, 6.3 Hz, 2H), 7.47 (dd, *J* = 39.4, 7.9 Hz, 1H), 7.40 – 7.33 (m, 6H), 7.31 – 7.24 (m, 5H), 7.04 (d, *J* = 8.5 Hz, 1H), 7.00 – 6.97 (m, 2H), 6.75 (dd, *J* = 9.2, 2.6 Hz, 2H), 6.60 (d, *J* = 9.4 Hz, 1H), 6.30 (dd, *J* = 24.3, 8.9 Hz, 1H), 5.13 – 5.06 (m, 1H), 4.74 – 4.70 (m, 1H), 4.63 (dd, *J* = 8.9, 5.2 Hz, 1H), 4.49 (s, 1H), 4.11 (d, *J* = 11.4 Hz, 1H), 3.93 – 3.85 (m, 1H), 3.70 – 3.62 (m, 4H), 3.61 – 3.52 (m, 3H), 3.41 (s, 2H), 3.24 (s, 4H), 3.08 (d, *J* = 5.0 Hz, 1H), 3.02 (dd, *J* = 13.9, 7.2 Hz, 1H), 2.85 (s, 1H), 2.55 – 2.44 (m, 9H), 2.40 – 2.29 (m, 8H), 2.27 – 2.08 (m, 11H), 1.92 – 1.87 (m, 1H), 1.56 (d, *J* = 15.1 Hz, 5H), 1.51 – 1.40 (m, 5H), 1.26 (d, *J* = 8.2 Hz, 9H), 1.04 (s, 9H), 0.94 (s, 3H).

HRMS (ESI) m/z [M + H]^+^ calcd. For C_83_H_108_ClF_3_N_11_O_11_S_4_ 1654.6748, found. 1654.6817.

**(2*S*,4*R*)-1-((2*S*)-2-(10-(4-((4'-Chloro-4-methyl-6-((4-(4-(((4-(((*R*)-4-morpholino-1-(phenylthio)butan-2-yl)amino)-3-((trifluoromethyl)sulfonyl)phenyl)sulfonyl)carbamoyl)phenyl)piperazin-1-yl)methyl)-2,3,4,5-tetrahydro-[1,1'-biphenyl]-4-yl)methyl)piperazin-1-yl)-10-oxodecanamido)-3,3-dimethylbutanoyl)-4-hydroxy-N-((*S*)-1-(4-(4-methylthiazol-5-yl)phenyl)ethyl)pyrrolidine-2-carboxamide** (**PPC10**): ^1^H NMR (600 MHz, CDCl_3_) δ 8.67 (s, 1H), 8.32 (s, 1H), 8.10 (dd, *J* = 9.2, 2.0 Hz, 1H), 7.70 (dd, *J* = 8.9, 4.4 Hz, 2H), 7.44 – 7.33 (m, 7H), 7.32 – 7.23 (m, 5H), 7.03 (d, *J* = 8.6 Hz, 1H), 6.98 (d, *J* = 8.3 Hz, 2H), 6.74 (d, *J* = 8.8 Hz, 2H), 6.60 (d, *J* = 9.5 Hz, 1H), 6.29 (dd, *J* = 17.3, 8.8 Hz, 1H), 5.07 (td, *J* = 7.2, 3.4 Hz, 1H), 4.70 (q, *J* = 7.8 Hz, 1H), 4.61 (dd, *J* = 8.9, 4.3 Hz, 1H), 4.49 (s, 1H), 4.10 (d, *J* = 11.5 Hz, 1H), 3.89 (s, 1H), 3.65 (q, *J* = 5.9 Hz, 5H), 3.61 – 3.53 (m, 3H), 3.42 (s, 2H), 3.25 (s, 4H), 3.09 (dd, *J* = 13.9, 5.0 Hz, 1H), 3.03 – 2.98 (m, 1H), 2.87 (s, 2H), 2.58 – 2.41 (m, 11H), 2.41 – 2.25 (m, 10H), 2.25 – 2.05 (m, 9H), 1.95 – 1.86 (m, 1H), 1.68 (ddd, *J* = 19.8, 14.8, 6.8 Hz, 2H), 1.45 (dd, *J* = 6.9, 2.9 Hz, 3H), 1.25 (s, 12H), 1.04 (s, 9H), 0.94 (s, 3H).

HRMS (ESI) m/z [M + H]^+^ calcd. For C_84_H_110_ClF_3_N_11_O_11_S_4_ 1668.6904, found. 1668.6983.

**(2*S*,4*R*)-1-((2*S*)-2-(11-(4-((4'-Chloro-4-methyl-6-((4-(4-(((4-(((*R*)-4-morpholino-1-(phenylthio)butan-2-yl)amino)-3-((trifluoromethyl)sulfonyl)phenyl)sulfonyl)carbamoyl)phenyl)piperazin-1-yl)methyl)-2,3,4,5-tetrahydro-[1,1'-biphenyl]-4-yl)methyl)piperazin-1-yl)-11-oxoundecanamido)-3,3-dimethylbutanoyl)-4-hydroxy-N-((*S*)-1-(4-(4-methylthiazol-5-yl)phenyl)ethyl)pyrrolidine-2-carboxamide** (**PPC11**): ^1^H NMR (600 MHz, CDCl_3_) δ 8.67 (s, 1H), 8.32 (d, *J* = 2.1 Hz, 1H), 8.09 (dd, *J* = 9.2, 2.0 Hz, 1H), 7.72 (dd, *J* = 9.0, 2.9 Hz, 2H), 7.43 – 7.34 (m, 7H), 7.31 – 7.23 (m, 5H), 7.01 (d, *J* = 8.6 Hz, 1H), 6.98 (d, *J* = 8.4 Hz, 2H), 6.75 (d, *J* = 9.0 Hz, 2H), 6.59 (d, *J* = 9.4 Hz, 1H), 6.25 (t, *J* = 9.2 Hz, 1H), 5.12 – 5.04 (m, 1H), 4.71 (td, *J* = 7.9, 3.2 Hz, 1H), 4.60 (dd, *J* = 8.8, 2.0 Hz, 1H), 4.50 (s, 1H), 4.11 (d, *J* = 11.5 Hz, 1H), 3.89 (dt, *J* = 8.0, 4.3 Hz, 1H), 3.65 (dt, *J* = 14.4, 7.1 Hz, 5H), 3.61 – 3.56 (m, 3H), 3.43 (s, 2H), 3.25 (d, *J* = 5.2 Hz, 4H), 3.09 (dd, *J* = 13.9, 5.0 Hz, 1H), 3.01 (dd, *J* = 13.9, 7.2 Hz, 1H), 2.86 (s, 2H), 2.59 – 2.45 (m, 9H), 2.44 – 2.26 (m, 12H), 2.25 – 2.04 (m, 9H), 1.93 (d, *J* = 17.2 Hz, 1H), 1.67 (dt, *J* = 14.2, 7.0 Hz, 1H), 1.46 (dd, *J* = 6.9, 1.7 Hz, 4H), 1.25 (s, 14H), 1.04 (s, 9H), 0.94 (s, 3H).

HRMS (ESI) m/z [M + H]^+^ calcd. For C_85_H_112_ClF_3_N_11_O_11_S_4_ 1682.7061, found. 1682.7142.

**(2*S*,4*R*)-1-((*S*)-2-(8-(4-(((*R*)-4'-chloro-4-methyl-6-((4-(4-(((4-(((*R*)-4-morpholino-1-(phenylthio)butan-2-yl)amino)-3-((trifluoromethyl)sulfonyl)phenyl)sulfonyl)carbamoyl)phenyl)piperazin-1-yl)methyl)-2,3,4,5-tetrahydro-[1,1'-biphenyl]-4-yl)methyl)piperazin-1-yl)-8-oxooctanamido)-3,3-dimethylbutanoyl)-4-hydroxy-N-((*S*)-1-(4-(4-methylthiazol-5-yl)phenyl)ethyl)pyrrolidine-2-carboxamide (753b)** and **(2*S*,4*R*)-1-((*S*)-2-(8-(4-(((*S*)-4'-chloro-4-methyl-6-((4-(4-(((4-(((*R*)-4-morpholino-1-(phenylthio)butan-2-yl)amino)-3-((trifluoromethyl)sulfonyl)phenyl)sulfonyl)carbamoyl)phenyl)piperazin-1-yl)methyl)-2,3,4,5-tetrahydro-[1,1'-biphenyl]-4-yl)methyl)piperazin-1-yl)-8-oxooctanamido)-3,3-dimethylbutanoyl)-4-hydroxy-N-((*S*)-1-(4-(4-methylthiazol-5-yl)phenyl)ethyl)pyrrolidine-2-carboxamide (753a)**: To a stirring suspension of the hydrochloride salt **7b** (50 mg, 0.045 mmol) or **7a** (50 mg, 0.045 mmol) in 3 mL DCM was added TEA (62 µL, 0.45 mmol) followed by known acid **8** (27 mg, 0.045 mmol) and HATU (19 mg ,0.05). The mixture was stirred at room temperature for 5 h. After completion of the reaction it was diluted with 50 mL DCM and washed with sat. NH_4_Cl solution (10 mL) followed by water (10 mL) and brine sol. (10 mL). The organic portion was dried over Na_2_SO_4_, and then evaporated in reduced pressure to obtain the crude. The crude was purified through flash silica gel chromatography (0% to 15% MeOH in DCM) to afford the compound **753b** (45 mg) or **753a** (43 mg) as white powder in 61% and 59% yield respectively.

Compound **753b**: (600 MHz, CDCl_3_) δ 8.67 (s, 1H), 8.31 (d, *J* = 2.2 Hz, 1H), 8.07 (d, *J* = 8.5 Hz, 1H), 7.72 (d, *J* = 8.5 Hz, 2H), 7.51 (d, *J* = 7.7 Hz, 1H), 7.38 (q, *J* = 8.3 Hz, 6H), 7.31 – 7.27 (m, 4H), 6.99 (d, *J* = 7.9 Hz, 3H), 6.72 (d, *J* = 8.4 Hz, 2H), 6.60 (d, *J* = 9.3 Hz, 1H), 6.26 (d, *J* = 8.5 Hz, 1H), 5.09 (p, *J* = 7.1 Hz, 1H), 4.71 (t, *J* = 8.0 Hz, 1H), 4.57 (d, *J* = 8.6 Hz, 1H), 4.49 (s, 1H), 4.11 (d, *J* = 11.4 Hz, 1H), 3.89 (s, 1H), 3.65 (q, *J* = 5.9 Hz, 4H), 3.57 (dd, *J* = 11.4, 3.5 Hz, 2H), 3.48 (s, 2H), 3.42 (s, 2H), 3.23 (s, 3H), 3.16 (q, *J* = 7.4 Hz, 2H), 3.10 (dd, *J* = 13.8, 5.0 Hz, 1H), 3.01 (dd, *J* = 13.8, 7.2 Hz, 1H), 2.50 (s, 4H), 2.40 – 2.33 (m, 4H), 2.32 (d, *J* = 7.5 Hz, 2H), 2.26 (t, *J* = 7.9 Hz, 5H), 2.13 – 2.06 (m, 3H), 1.71 – 1.62 (m, 2H), 1.59 – 1.51 (m, 5H), 1.47 (dt, *J* = 7.4, 4.0 Hz, 5H), 1.43 (d, *J* = 6.7 Hz, 9H), 1.27 (s, 4H), 1.04 (s, 10H), 0.96 (s, 3H).

^13^C NMR (150 MHz, Chloroform-d) δ 174.1, 172.3, 172.0, 170.1, 165.8, 154.3, 151.9, 150.6, 148.6, 143.5, 141.7, 138.2, 135.7, 135.1, 134.8, 132.5, 131.8, 131.3, 131.0, 130.2, 129.8, 129.7, 129.5, 128.7, 128.6, 127.6, 126.7, 113.7, 113.3, 108.9, 70.1, 68.2, 66.9, 60.6, 58.8, 57.7, 57.1, 56.2, 55.8, 54.7, 53.8, 52.5, 50.9, 49.0, 47.2, 46.3, 42.3, 39.3, 37.7, 36.4, 35.9, 35.4, 34.6, 33.3, 32.1, 30.5, 30.3, 29.1, 28.8, 26.7, 25.5, 25.3, 23.5, 22.4, 16.3.

HRMS (ESI) m/z [M + H]^+^ calcd. For C_82_H_106_ClF_3_N_11_O_11_S_4_ 1640.6591, found. 1640.6676.

Compound **753a:** (600 MHz, CDCl_3_) δ 8.67 (s, 1H), 8.32 (s, 1H), 8.08 (d, *J* = 9.2 Hz, 1H), 7.71 (d, *J* = 8.2 Hz, 2H), 7.44 (s, 1H), 7.40 – 7.35 (m, 6H), 7.31 – 7.27 (m, 4H), 6.99 (d, *J* = 8.2 Hz, 3H), 6.74 (d, *J* = 8.3 Hz, 2H), 6.60 (d, *J* = 9.3 Hz, 1H), 6.28 (d, *J* = 8.7 Hz, 1H), 5.08 (p, *J* = 7.1 Hz, 1H), 4.71 (t, *J* = 8.0 Hz, 1H), 4.60 (d, *J* = 8.4 Hz, 1H), 4.49 (s, 1H), 4.12 (d, *J* = 11.4 Hz, 1H), 3.89 (s, 1H), 3.65 (q, *J* = 6.1 Hz, 4H), 3.60 – 3.51 (m, 3H), 3.42 (s, 2H), 3.23 (s, 3H), 3.16 (q, *J* = 7.4 Hz, 1H), 3.12 – 3.08 (m, 1H), 3.04 – 2.98 (m, 1H), 2.51 (s, 3H), 2.42 (s, 4H), 2.39 – 2.33 (m, 4H), 2.31 (d, *J* = 7.0 Hz, 2H), 2.26 (s, 4H), 2.20 – 2.17 (m, 2H), 2.12 – 2.05 (m, 3H), 1.70 – 1.63 (m, 2H), 1.57 (s, 5H), 1.50 – 1.46 (m, 5H), 1.44 (d, *J* = 6.6 Hz, 7H), 1.26 (d, *J* = 10.4 Hz, 4H), 1.04 (s, 10H), 0.95 (s, 3H).

HRMS (ESI) m/z [M + H]^+^ calcd. For C_82_H_106_ClF_3_N_11_O_11_S_4_ 1640.6591, found. 1640.6665.

**NMR Spectra:**

**Supplementary Fig. 14.** ^1^H NMR of **2/2b/2a** (CDCl_3_, 600 MHz)

**Supplementary Fig. 15.** ^13^C NMR of **2/2b/2a** (CDCl_3_, 150 MHz)

**Supplementary Fig. 16.** ^1^H NMR of **3/3b/3a** (CDCl_3_, 600 MHz)

**Supplementary Fig. 17.** ^13^C NMR of **3/3b/3a** (CDCl_3_, 150 MHz)

**Supplementary Fig. 18.** ^1^H NMR of **4/4b/4a** (CDCl_3_, 600 MHz)

**Supplementary Fig. 19.** ^13^C NMR of **4/4b/4a** (CDCl_3_, 150 MHz)

**Supplementary Fig. 20.** ^1^H NMR of **6** (CDCl_3_, 600 MHz)

**Supplementary Fig. 21.** ^1^H NMR of **6b** (CDCl_3_, 600 MHz)

**Supplementary Fig. 22.** ^13^C NMR of **6b** (CDCl_3_, 150 MHz)

**Supplementary Fig. 23.** ^1^H NMR of **6a** (CDCl_3_, 600 MHz)

**Supplementary Fig. 24.** ^1^H NMR of **PPC5** (CDCl_3_, 600 MHz)

**Supplementary Fig. 25.** ^1^H NMR of **PPC6** (CDCl_3_, 600 MHz)

**Supplementary Fig. 26.** ^1^H NMR of **PPC7** (CDCl_3_, 600 MHz)

**Supplementary Fig. 27.** ^1^H NMR of **PPC8** (CDCl_3_, 600 MHz)

**Supplementary Fig. 28.** ^1^H NMR of **PPC9** (CDCl_3_, 600 MHz)

**Supplementary Fig. 29.** ^1^H NMR of **PPC10** (CDCl_3_, 600 MHz)

**Supplementary Fig. 30.** ^1^H NMR of **PPC11** (CDCl_3_, 600 MHz)

**Supplementary Fig. 31.** ^1^H NMR of **753b** (CDCl_3_, 600 MHz)

**Supplementary Fig. 32.** ^13^C NMR of **753b** (CDCl_3_, 150 MHz)

**Supplementary Fig. 33.** ^1^H NMR of **753a** (CDCl_3_, 600 MHz)

**Supplementary References**

1. Pal, P.; Thummuri, D.; Lv, D.; Liu, X.; Zhang, P.; Hu, W.; Poddar, S. K.; Hua, N.; Khan, S.; Yuan, Y.; Zhang, X.; Zhou, D.; Zheng, G. Discovery of a Novel BCL‑X_L_ PROTAC Degrader with Enhanced BCL‑2 Inhibition. *J. Med. Chem.* **2021**, *64*, 14230−14246.

2. Wang, G.; Zhang, H.; Zhou, J.; Ha, C.; Pei, D.; Ding, K. An efficient synthesis of ABT-263, a novel inhibitor of antiapoptotic Bcl-2 proteins. *Synthesis.* **2008**, *15*, 2398–2404.

3. Zhang, X.; Thummuri, D.; Liu, X.; Hu, W.; Zhang, P.; Khan, S.; Yuan, Y.; Zhou, D.; Zheng, G. Discovery of PROTAC BCL-XL degraders as potent anticancer agents with low on-target platelet toxicity. *Eur. J. Med. Chem*. **2020**, *192*, 112186–112210.
