# Supplementary material for: Development of a BCL-xL and BCL-2 dual degrader with improved anti-leukemic activity,
Source: Nat Commun. 2021 Nov 25;12:6896. doi: 10.1038/s41467-021-27210-x (PMC8617031; doi:10.1038/s41467-021-27210-x)
Supplement: Supplementary file 2 — Description of Additional Supplementary Files [file 41467_2021_27210_MOESM2_ESM.docx]

**Description of Additional Supplementary Files**

**File Name:** Supplementary Data 1

**Description:** Coordinates of generated structural models. Both PDB format and PyMol format files are included in the ZIP file.

**File Name:** Supplementary Movie 1

**Description:** Movie of the first major motion mode of CRLVHL/DT2216/BCL-xL/E2-Ub/RBX1.

**File Name:** Supplementary Movie 2

**Description:** Movie of the second major motion mode of CRLVHL/DT2216/BCL-xL/E2-Ub/RBX1.
