# Supplementary figures and images for: Development of a BCL-xL and BCL-2 dual degrader with improved anti-leukemic activity,
Source: Nat Commun. 2021 Nov 25;12:6896. doi: 10.1038/s41467-021-27210-x (PMC8617031; doi:10.1038/s41467-021-27210-x)

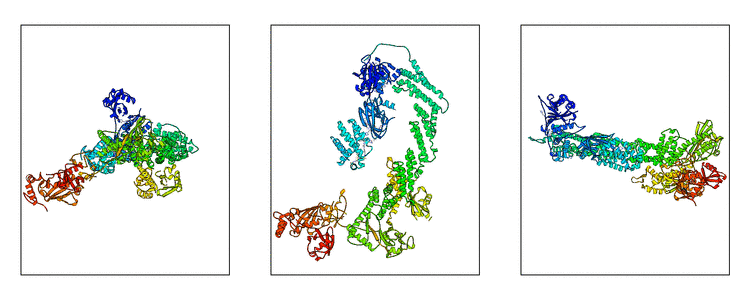

Supplement: Supplementary file 4 — Supplementary Movie 1 [file 41467_2021_27210_MOESM4_ESM.gif]

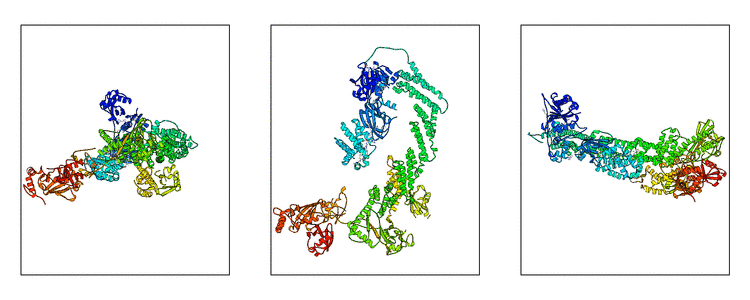

Supplement: Supplementary file 5 — Supplementary Movie 2 [file 41467_2021_27210_MOESM5_ESM.gif]
