# Supplementary material for: Development of a BCL-xL and BCL-2 dual degrader with improved anti-leukemic activity,
Source: Nat Commun. 2021 Nov 25;12:6896. doi: 10.1038/s41467-021-27210-x (PMC8617031; doi:10.1038/s41467-021-27210-x)
Supplement: Supplementary file 6 — Reporting Summary [file 41467_2021_27210_MOESM6_ESM.pdf]

nature research

Corresponding author(s): Daohong Zhou

Last updated by author(s): Nov 3, 2021

Reporting Summary

Nature Research wishes to improve the reproducibility of the work that we publish. This form provides structure for consistency and transparency in reporting. For further information on Nature Research policies, see our Editorial Policies and the Editorial Policy Checklist.

Statistics

For all statistical analyses, confirm that the following items are present in the figure legend, table legend, main text, or Methods section.

n/a	Confirmed	
[ ]	[x]	The exact sample size (*n*) for each experimental group/condition, given as a discrete number and unit of measurement
[ ]	[x]	A statement on whether measurements were taken from distinct samples or whether the same sample was measured repeatedly
[ ]	[x]	The statistical test(s) used AND whether they are one- or two-sided<br>*Only common tests should be described solely by name; describe more complex techniques in the Methods section.*
[x]	[ ]	A description of all covariates tested
[x]	[ ]	A description of any assumptions or corrections, such as tests of normality and adjustment for multiple comparisons
[ ]	[x]	A full description of the statistical parameters including central tendency (e.g. means) or other basic estimates (e.g. regression coefficient) AND variation (e.g. standard deviation) or associated estimates of uncertainty (e.g. confidence intervals)
[ ]	[x]	For null hypothesis testing, the test statistic (e.g. *F*, *t*, *r*) with confidence intervals, effect sizes, degrees of freedom and *P* value noted<br>*Give P values as exact values whenever suitable.*
[x]	[ ]	For Bayesian analysis, information on the choice of priors and Markov chain Monte Carlo settings
[x]	[ ]	For hierarchical and complex designs, identification of the appropriate level for tests and full reporting of outcomes
[x]	[ ]	Estimates of effect sizes (e.g. Cohen's *d*, Pearson's *r*), indicating how they were calculated

Our web collection on statistics for biologists contains articles on many of the points above.

Software and code

Policy information about availability of computer code

Data collection	Image Lab Touch version 2.2.0.08 Software (Bio-rad, Hercules, CA, USA) was used for scanning all immunoblots on the ChemiDoc MP imaging system; Gen5 version 3.04 software (BioTek, Winooski, VT, USA) was used for absorbance and luminescence measurements on Synergy Neo2 multi-mode plate reader; FlowJo V10 software was used for the acquisition of flow cytometry data.
Data analysis	ImageJ v1.53a software (NIH) was used for the quantification of immunoblots; GraphPad Prism v7 or v9 (GraphPad Software, La Jolla, CA, USA) was used for the preparation of all the graphs, determination of half maximal effective concentration (EC50) values, Inhibition constant (Ki), and the statistical analyses; FlowJo V10 software was used to analyze the flow cytometry data. PyMol version 2.4.0a0 was used to perform molecular visualization, alignment, refinement, and RMSD calculation; OpenEye Toolkit version 2018.Nov.3 was used for molecular format converting, protonation status assigning , 3D conformation generation; PRosettaC (GitHub commit number 9cc9e11d045c9961fbdb667e700a98a46723a45b) was used to predicting the ternary complex; PatchDock version 1.0 was used for protein-protein docking (called by PRosettaC); Rosetta version 2019.35.60890 was used for structural optimization and energy computation (called by PRosettaC); ENM module of DynOmics portal version 1.0 was used to calculate the normal mode of complex.

For manuscripts utilizing custom algorithms or software that are central to the research but not yet described in published literature, software must be made available to editors and reviewers. We strongly encourage code deposition in a community repository (e.g. GitHub). See the Nature Research guidelines for submitting code & software for further information.

Data

Policy information about availability of data

All manuscripts must include a data availability statement. This statement should provide the following information, where applicable:

- Accession codes, unique identifiers, or web links for publicly available datasets
- A list of figures that have associated raw data
- A description of any restrictions on data availability

Data availability
The raw immunoblot images are supplied as source data. Source data are provided with this paper. The primers used in this study are also listed in Supplementary Table 1. The structural models generated in this study are provided in the Supplementary Data 1. The x-ray crystal structure data used in this study are available in the PDBbank database under accession code 3DQV [http://doi.org/10.2210/pdb3DQV/pdb], 4LVT [http://doi.org/10.2210/pdb4LVT/pdb], 4P5O [http://doi.org/10.2210/pdb4P5O/pdb], 4QNQ [http://doi.org/10.2210/pdb4QNQ/pdb], 4V3K [http://doi.org/10.2210/pdb4V3K/pdb], 4W9F [http://doi.org/10.2210/pdb4W9F/pdb], and 5N4W [http://doi.org/10.2210/pdb5N4W/pdb]. The authors declare that data supporting the findings of this study are available within the paper.
Code availability
All software/server used in this study (PyMol, OpenEye Toolkit, PRosettaC, PatchDock, DynOmics) could be accessed with academic free license. The PyMol script (including example and description) of automatically sampling dihedral angels for generating E2/POI contacting model could be accessed from GitHub repository: https://github.com/lezephyr1988/sample_ternary_dihedral. All the input files used for generating structural model with PRosettaC could be accessed from GitHub repository: https://github.com/lezephyr1988/BCLxl-BCL2.

Field-specific reporting

Please select the one below that is the best fit for your research. If you are not sure, read the appropriate sections before making your selection.

[x] Life sciences [ ] Behavioural & social sciences [ ] Ecological, evolutionary & environmental sciences

For a reference copy of the document with all sections, see nature.com/documents/nr-reporting-summary-flat.pdf

Life sciences study design

All studies must disclose on these points even when the disclosure is negative.

Sample size	Statistical methods were not used to determine sample sizes.<br>However, for quantification purposes, a sample size of n = 2 or 3 unless otherwise stated (reproduced two or more independent times) was used to assess reproducibility and robustness of each experiment performed. Sample sizes were based on prior study in the field (For example: Alabi S, Jaime-Figueroa S, Yao Z, et al. Mutant-selective degradation by BRAF-targeting PROTACs[J]. Nature communications, 2021, 12(1): 1-11.).
Data exclusions	In general, no data were excluded from the analyses.
Replication	To ensure reproducibility of experimental findings, all biochemical or cellular assays were repeated independently at least two times. One representative result for each experiment is presented in the main Figures or the Extended Data Figures. We confirm that all attempts of replication were successful.
Randomization	Cultured cells were passaged evenly (random distribution) into dishes or flasks for treatment with PROTACs and/or indicated compounds. Due to dose-response nature of the experiments performed, randomization of the samples post-treatment would make data interpretation impossible.
Blinding	Investigators were not blinded to the nature of their samples during data collection and analysis based on prior study in the field (for example: Alabi S, Jaime-Figueroa S, Yao Z, et al. Mutant-selective degradation by BRAF-targeting PROTACs[J]. Nature communications, 2021, 12(1): 1-11.).

Reporting for specific materials, systems and methods

We require information from authors about some types of materials, experimental systems and methods used in many studies. Here, indicate whether each material, system or method listed is relevant to your study. If you are not sure if a list item applies to your research, read the appropriate section before selecting a response.

Materials & experimental systems

n/a	Involved in the study	
	☒	Antibodies
	☒	Eukaryotic cell lines
☒		Palaeontology and archaeology
☒		Animals and other organisms
☒		Human research participants
☒		Clinical data
☒		Dual use research of concern

Methods

n/a	Involved in the study	
☒		ChIP-seq
	☒	Flow cytometry
☒		MRI-based neuroimaging

Antibodies

Antibodies used

Antibodies purchased from Cell Signaling Technologies (CST) and the dilutions are as follows: BCL-xL (Cat No. 2762S, 1:1000), BCL-2 (Cat No. 2870S, 1:1000), VHL (Cat No. 68547S, 1:1000), Tubulin (Cat No. 2146S, 1:1000), Caspase-3 (Cat No. 9662S, 1:1000), Cleaved Caspase-3 (Cat No. 9664S, 1:1000), Flag-tag (Cat No. 14793S, 1:1000), HA-tag (Cat No. 3724S, 1:1000). β-actin antibody was purchased from MP Biomedicals (Cat No. 8691001, 1:20 000).

Validation

All the used antibodies are commercially available. The antibodies used in a specific species or application have been validated by manufacturers to be used in that species/application and this information is provided in their website and/or antibody datasheets.
CST Antibody Validation Principles
To ensure our antibodies will work in your experiment, we adhere to the Hallmarks of Antibody Validation™, six complementary strategies that can be used to determine the functionality, specificity, and sensitivity of an antibody in any given assay. CST adapted the work by Uhlen, et. al., ("A Proposal for Validation of Antibodies." Nature Methods (2016)) to build the Hallmarks of Antibody Validation, based on our decades of experience as an antibody manufacturer and our dedication to reproducible science.
CST Hallmarks of Antibody Validation
We guarantee that our antibodies are fit for purpose by carefully tailoring the combination of validation strategies applied to each product. This means customizing our validation process according to the biological role of the target, while considering the sensitivity requirements of the downstream assay, the availability of appropriate testing models, and the relevance of each method to target investigation.
1. Binary Model: Antibody signal is measured in model systems with known presence/absence of target signal. Includes wild-type vs. genetic knockout, targeted induction or silencing.
2. Ranged Expression: Antibody signal strength is measured in cell lines or tissues representing a known continuum of target expression levels. Includes siRNA and heterozygous knockout assays.
3. Orthogonal Data: Antibody signal is correlated to target expression in model systems measured using antibody independent assays. Includes mass spectrometry and in situ hybridization.
4. Multiple Antibodies: Antibody signal is compared to the signal observed using antibodies targeting nonoverlapping epitopes of the target. Includes IP, ChIP, and ChIP-seq.
5. Heterologous Expression: Antibody signal is evaluated in cell lines following heterologous expression of native (or mutated) target protein.
6. Complementary Assays: Antibody specificity may be validated using complementary assays. Includes competitive ELISA, peptide dot blots, peptide blocking, or protein arrays.
The following antibodies from CST were used in this study.
BCL-xL (Cat No. 2762S, 1:1000)
https://www.cellsignal.com/products/primary-antibodies/bcl-xl-antibody/2762
BCL-2 (Cat No. 2870S, 1:1000)
https://www.cellsignal.com/products/primary-antibodies/bcl-2-50e3-rabbit-mab/2870
VHL (Cat No. 68547S, 1:1000)
https://www.cellsignal.com/products/primary-antibodies/vhl-antibody/68547?site-search-type=Products&N=4294956287&Ntt=vhl&fromPage=plp
Tubulin (Cat No. 2146S, 1:1000)
https://www.cellsignal.com/products/primary-antibodies/b-tubulin-antibody/2146
Caspase-3 (Cat No. 9662S, 1:1000)
https://www.cellsignal.com/products/primary-antibodies/caspase-3-antibody/9662
Cleaved Caspase-3 (Cat No. 9664S, 1:1000)
https://www.cellsignal.com/products/primary-antibodies/cleaved-caspase-3-asp175-5a1e-rabbit-mab/9664
Flag-tag (Cat No. 14793S, 1:1000)
https://www.cellsignal.com/products/primary-antibodies/dykddddk-tag-d6w5b-rabbit-mab-binds-to-same-epitope-as-sigma-s-anti-flag-m2-antibody/14793
HA-tag (Cat No. 3724S, 1:1000)
https://www.cellsignal.com/products/primary-antibodies/ha-tag-c29f4-rabbit-mab/3724

Antibody from MP Biomedicals (β-actin, Cat No. 8691001, 1:20 000).
Validated in our previous publication (https://doi.org/10.1038/s41591-019-0668-z)

Eukaryotic cell lines

Policy information about cell lines

Cell line source(s)	HEK293T (293T, Cat. No. ACS-4500), Hela (Cat. No. CCL-2), Kasumi-1 (Cat. No. CRL-2724) cells were recently purchased from American Type Culture Collection (ATCC, Manassas, VA, USA). Human platelet-rich plasma (PRP) was purchased from Zenbio (cat. no. SER-PRP-SDS).
Authentication	The cell lines have been validated by the suppliers. The cell lines purchased from ATCC were purchased as 'Certified Reference Material' stocks. ATCC authenticates cell lines using morphology, karyotyping and STR profiling
Mycoplasma contamination	Cell lines were recently purchased from ATCC and were not further tested for mycoplasma contamination in our laboratory, but cell proliferation rate and morphology of all the cell lines were continuously monitored.
Commonly misidentified lines (See ICLAC register)	none

Flow Cytometry

Plots

Confirm that:

- [x] The axis labels state the marker and fluorochrome used (e.g. CD4-FITC).
- [x] The axis scales are clearly visible. Include numbers along axes only for bottom left plot of group (a 'group' is an analysis of identical markers).
- [x] All plots are contour plots with outliers or pseudocolor plots.
- [x] A numerical value for number of cells or percentage (with statistics) is provided.

Methodology

Sample preparation	Kasumi-1 cells were plated in 12 well plates at 0.2 × 106 cells/well and treated with DMSO, 0.1 or 1 µM DT2216, 753b, ABT-199, or ABT263 for 24 h. After treatment, cells were stained with Annexin V-FLUOS (1: 200, Cat. No. 11828681001, Roche, Little Falls, NJ, USA) at room temperature for 15 min, washed with PBS and then added DAPI (0.2 µg/mL, Cat. No. D1306, Invitrogen, Waltham, MA, USA) for apoptosis flow cytometry on Gallios (Beckman, Indianapolis, IN, USA).
Instrument	Gallios, Beckman Coulter
Software	FlowJo, v10
Cell population abundance	All samples were collected at least 50,000 events of live cells.
Gating strategy	FSC/SSC was used to distinguish the cell size. PI was used to gate the live cells and all live cells were analyzed.

- [x] Tick this box to confirm that a figure exemplifying the gating strategy is provided in the Supplementary Information.
